# PCBP1 binding to single-stranded poly-cytosine motifs enhances cGAS sensing and impairs breast cancer development

**DOI:** 10.1038/s42003-025-09456-z

**Published:** 2026-01-07

**Authors:** Cécile Fréreux, Joseph A. Q. Karam, Breege V. Howley, Bryan Granger, Paramita Chakraborty, Silvia Vaena, Martin Romeo, Annamarie C. Dalton, Bidyut K. Mohanty, Shikhar Mehrotra, Philip H. Howe

**Affiliations:** 1 Department of Biochemistry and Molecular Biology, Medical University of South Carolina, Charleston, SC USA; 2 Bioinformatics Core, Medical University of South Carolina, Charleston, SC USA; 3 Department of Surgery, Hollings Cancer Center, Medical University of South Carolina, Charleston, SC USA; 4 Hollings Cancer Center, Medical University of South Carolina, Charleston, SC USA; 5 Department of Cell Biology and Physiology, Edward Via College of Osteopathic Medicine, Spartanburg, SC USA

**Keywords:** Breast cancer, DNA, Cell signalling, Tumour-suppressor proteins

## Abstract

The cGAS-STING pathway plays a central role in controlling tumor progression through nucleic acid sensing and type I Interferon production. Here, we identify Poly(rC) Binding Protein 1 as a tumor suppressor that amplifies cGAS-STING signaling in breast cancer. Using patient datasets and a transgenic mouse model with conditional PCBP1 knockout in mammary epithelial cells, we show that PCBP1 expression correlates with improved survival, reduced tumor burden, increased type I Interferon and Interferon Stimulated Gene expression, and elevated cytotoxic T cell infiltration. Mechanistically, PCBP1 binds cytosine-rich single-stranded motifs via its KH domains and increases cGAS affinity to these nucleic acids. Mutation of PCBP1’s conserved GXXG loops impairs nucleic acid binding and cGAS activation. Although cGAS is a double-stranded DNA sensor with no intrinsic sequence specificity, we uncover that the single-stranded nucleic-acid binding protein PCBP1 enhances cGAS sensing by engaging sequence-specific motifs, acting as a nucleic acid co-sensor that impairs tumorigenesis.

## Abstract (web summary)

Sequence-specific binding of PCBP1 to poly-cytosine DNA enhances cGAS-STING activation and interferon-driven immune responses, revealing a new co-sensing mechanism that restrains breast tumor growth.

## Introduction

Cyclic GMP-AMP synthase (cGAS) is a Pattern Recognition Receptor, primarily known for sensing cytosolic double-stranded DNA (dsDNA) to trigger type I IFN responses^1–3^ and promote antiviral defense or anti-tumor responses. In cancer cells, cytosolic nucleic acids accumulate due to genomic instability, DNA damage, defective DNA repair mechanisms, and mitochondrial stress^4–6^, acting as danger-associated molecular patterns (DAMPs) that activate cGAS.

Upon binding dsDNA, cGAS undergoes conformational changes, catalysing the production of the second messenger cyclic GMP-AMP (2’3’-cGAMP), which binds and activates the adaptor protein STING (stimulator of interferon genes)^7–9^. This activation initiates a cascade of downstream signalling events, including the recruitment and activation of TANK-binding kinase 1 (TBK1). Phosphorylated TBK1 activates and phosphorylates STING as well as the transcription factor interferon regulatory factor 3 (IRF3). Phosphorylated IRF3 dimerizes and translocates into the nucleus to induce type I IFN expression. Secreted IFNs then act in both autocrine and paracrine fashion by binding to the IFNAR1/2 receptor complex, activating the JAK–STAT pathway. This leads to STAT1 and STAT2 phosphorylation, dimerization, and complex formation with IRF9, which drives the expression of interferon-stimulated genes (ISGs)^10–12^. In the tumor microenvironment, this pathway enhances cancer cell immune detection by promoting antigen presentation^4,13–15^ and the secretion of chemokines such as CXCL9/10, which recruit cytotoxic T cells to the tumor^16–18^. In addition, STING signaling contributes to cellular senescence^19^ and its epigenetic silencing in proliferating disseminated tumor cells promotes immune evasion from NK and cytotoxic T cells, thereby enabling the reawakening of dormant cells and metastatic outgrowth at secondary sites^20^.

To prevent aberrant activation by self-DNA and over-inflammation, cGAS is spatially restricted: it resides predominantly in the cytosol or is sequestered in an inactive form in the nucleus^21–24^, while genomic and mitochondrial DNA are normally confined to their respective compartments. However, in the context of DNA damage or cellular stress, self-DNA may accumulate in the cytoplasm. Despite this, cGAS does not display intrinsic sequence specificity, binding DNA primarily through electrostatic interactions with the phosphate backbone^2,25^. Its activation is strongly influenced by DNA structure and length: it senses most efficiently dsDNA over 50 base pairs^26,27^, or Y-form DNA composed of short duplexes with single-stranded overhangs^28^. Despite these structural preferences, the question of whether cGAS activity is regulated by DNA sequence composition remains largely unknown.

Poly(rC)-binding protein 1 (PCBP1), or hnRNP E1, is also a nucleic acid-binding protein, but, unlike cGAS, it recognizes specifically poly-cytosine tracts on single-stranded DNA or RNA via its three KH domains, which cannot be involved in dsDNA binding^29–32^. PCBP1 regulates mRNA translation, alternative splicing, and transcription, and acts as a tumor suppressor by inhibiting epithelial-to-mesenchymal transition (EMT)^33–35^. These functions are negatively regulated by non-canonical TGF-β signaling, which promotes PCBP1 phosphorylation and dissociation from its RNA targets^33,36^. Loss or inactivation of PCBP1 promotes EMT and tumor progression. Beyond these roles, one recent study has shown that PCBP1 also contributes to anti-tumor immunity in T cells by preventing their conversion into regulatory T cells^37^. However, its role in nucleic acid sensing in tumor cells has never been explored, and its influence on the tumor immune microenvironment remains poorly understood.

In this study, we show that the single-stranded nucleic acid-binding protein PCBP1 facilitates cGAS activation through the recognition of poly-cytosine single-stranded DNA in mammary epithelial cells. PCBP1 acts as a co-sensor enhancing cGAS affinity for these nucleic acids, inducing a more efficient 2’3’-cGAMP production at sub-saturating nucleic acid concentrations. This further enhances type I Interferon and Interferon-Stimulated Gene responses, promotes CD8 + T cells infiltration and impairs tumor growth. The discovery that PCBP1 selectively promotes the sensing of single-stranded polyC motifs highlights a previously unrecognized mechanism of cGAS regulation, amplifying the cGAS-STING response when nucleic acids harboring these motifs are present in the cytoplasm and thereby influencing tumor immune surveillance and tumorigenesis.

## Results

### PCBP1 expression impairs mouse mammary tumor formation and increases survival probability in human breast cancer patients

**Fig. 1:**
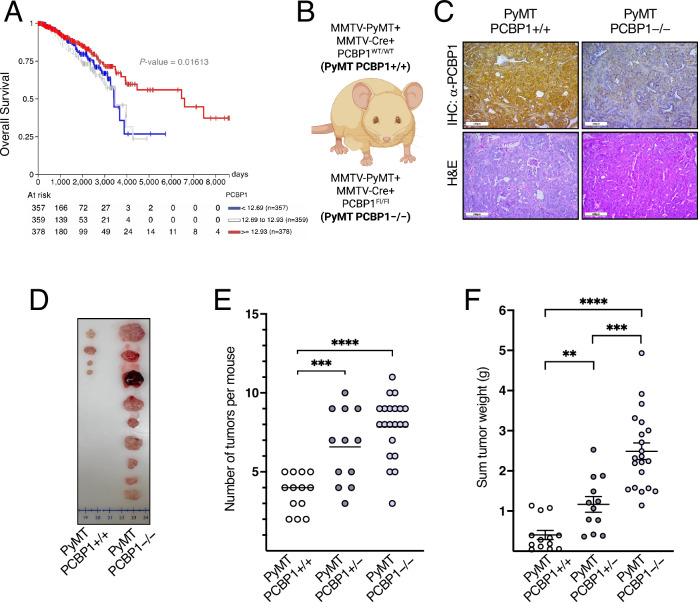
PCBP1 impairs mouse mammary tumor formation and increases survival probability in human breast cancer patients. **A** Kaplan–Meier analysis for TCGA breast cancer patients representing survival probability over time for low (blue), medium (white) and high (red) expression of PCBP1 mRNA. **B** Representation of the murine PyMT Pcbp1+/+ vs. PyMT Pcbp1–/– breast cancer models. Created in BioRender. Frereux, C. (2025) https://BioRender.com/gnssy36**C** Immunohistochemical analysis of PCBP1 and hematoxylin/eosin staining of PyMT *Pcbp1*+/+ and PyMT *Pcbp1*–/– tumors (scale bars: 100 µm). **D** Representative images of all the tumors collected in one PyMT *Pcbp1*+/+ mouse vs. one PyMT *Pcbp1*–/– mouse. **E** Number of tumors, and **F** average total tumor burden per PyMT *Pcbp1*+/+ (*n* = 13), PyMT *Pcbp1*+/− (*n* = 12) and PyMT *Pcbp1*–/– (*n* = 21) mouse. (Mean ± SEM, two-tailed unpaired *t* test for two-by-two analysis and one-way ANOVA, ****P value < 0.0001 for all three groups analyzed.

We and others have previously shown that PCBP1 exerts tumor-suppressive functions and is frequently downregulated in multiple cancer types, including colon, ovarian, and lung adenocarcinomas, as well as in peritoneal metastases of gastric cancer^38–40^. To test its relevance in breast cancer, we analyzed TCGA (The Cancer Genome Atlas) breast cancer patient data using the UCSC Xena platform^41^, which revealed that high *PCBP1* mRNA expression is significantly associated with improved overall survival (Fig. 1A).

To further investigate the anti-tumorigenic role of PCBP1, we generated a C57BL/6 transgenic immunocompetent mouse model expressing the MMTV-PyMT (polyomavirus middle T antigen) oncogene, which drives mammary tumorigenesis. The MMTV-PyMT model is widely used in breast cancer research as it recapitulates the histological and molecular progression of human breast cancer patients with the development of multifocal adenocarcinoma and metastatic lesions to the lungs. As tumors progress, they lose estrogen and progesterone receptor and often gain androgen receptor expression, making this model suitable for studying triple-negative breast cancer (TNBC), particularly the luminal AR (androgen receptor–positive) TNBC subtype^42^. In addition to the MMTV-PyMT construct, our mice expressed an MMTV-Cre *Pcbp1*(fl/fl) system that selectively knocks out PCBP1 in mammary epithelial cells. These mice, hereafter referred to as PyMT *Pcbp1*–/–, were compared to control animals expressing the MMTV-PyMT and MMTV-Cre constructs along with the non-floxed Pcbp1(wt/wt) alleles, referred to as PyMT *Pcbp1*+/+ (Fig. 1B). The loss of PCBP1 in PyMT-driven mammary tumors, as confirmed by immunohistochemistry staining (Fig. 1C), resulted in a dramatic increase in both tumor number (Fig. 1D, E) and total tumor burden (Fig. 1D, F) compared to *Pcbp1*-proficient controls. Heterozygous PyMT *Pcbp1*+/− tumors displayed an intermediate phenotype, with a partial increase in tumor number (Fig. 1E) and burden (Fig. 1F) compared to PyMT *Pcbp1*+/+ controls, suggesting a dose-dependent effect of PCBP1 on tumor suppression.

Overall, these results demonstrate that PCBP1 exerts tumor-suppressive effects in our C57BL/6 immunocompetent mouse model of breast cancer.

### PCBP1 induces type I interferons and interferon-stimulated genes signaling in mammary cells

**Fig. 2:**
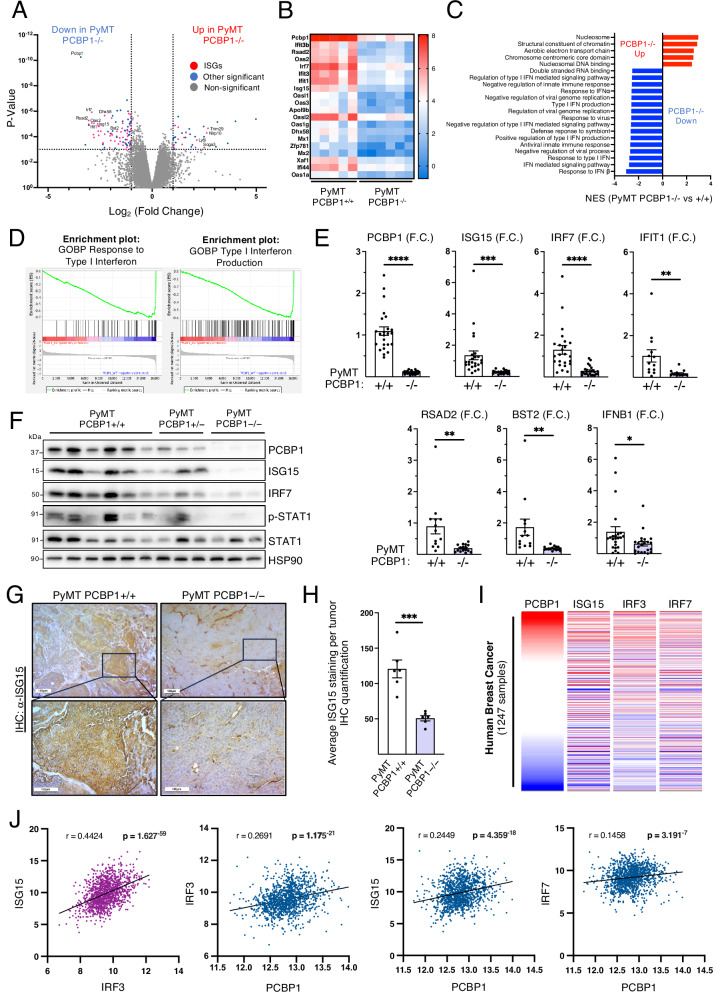
PCBP1 promotes type I IFNs and ISG’s signaling in mammary tumors and breast cancer patient datasets. **A** Volcano plot of the RNA-sequencing analysis representing the genes differentially expressed between PyMT *Pcbp1*–/– (*n* = 6) and PyMT *Pcbp1*+/+ (*n* = 5) mammary tumors. **B** Heat map showing the relative expression value (Log2 normalized counts CPM) of top 20 downregulated protein-coding genes (*p* < 0.001) in PyMT *Pcbp1*–/– versus PyMT *Pcbp1*+/+ tumors ranked by fold change. **C** GSEA analysis with MSigDB M5 (ontology), based on RNA-sequencing results, representing the normalized enrichment score of significantly upregulated and downregulated gene sets in PyMT *Pcbp1*–/– versus PyMT *Pcbp1*+/+ tumors. **D** Enrichment plots of ‘Response to Type I Interferon’ (NES = −2.76, FDR *q* value < 0.0001) and ‘Type I Interferon Production’ (NES = −2.60, FDR *q* value < 0.0001) gene sets based on GSEA analysis (M5 ontology). **E** RT-qPCR analysis of *Pcbp1* and several ISGs’ expression: *Isg15, Irf7, Ifit11, Rsad2* and *Bst2*, and of *Ifnb1* in PyMT *Pcbp1*–/– (*n* = 23 except for *Ifit1, Bst2, Rsad2* *n* = 16) and PyMT *Pcbp1*+/+ (*n* = 24, except for *Ifit1, Bst2, Rsad2* *n* = 13) tumors (mean ± SEM, two-tailed unpaired *t* test). **F** Western blot analysis of HSP90, ISG15, IRF7 and a downstream activation marker of IFN signaling: phospho-STAT1 as well as total STAT1 in PyMT *Pcbp1*+/+, +/–, and –/– tumors. **G** Immunohistochemical analysis of ISG15 in PyMT *Pcbp1*+/+ and PyMT *Pcbp1*–/– tumors (scale bars: 100 µm) and **H** its quantification (mean ± SEM, two-tailed unpaired *t* test). **I** Cross-correlation between the expression of *PCBP1* and the interferon-stimulated genes *ISG15, IRF3* and *IRF7* in 1247 human breast tumor samples from the Cancer Genome Atlas BRCA RNA-sequencing database. **J** Correlation analysis between *PCBP1* and ISG expression. *ISG15* over *IRF3* expression is provided as a positive correlation control (left panel). Gene expression is represented in Log2 (normalized count + 1), and Pearson’s rho (*r* = ) and *P* value (*p* = ) are provided for each correlation.

To investigate the molecular mechanisms underlying the increased tumor burden observed upon *Pcbp1* silencing, we performed high-throughput RNA sequencing on PyMT-driven mammary tumors from 18-week-old *Pcbp1*+/+ and *Pcbp1*–/– mice. Among the 58 most significantly downregulated genes (*p* < 0.001, fold change <–2) in *Pcbp1*-deficient tumors, 34 were annotated as interferon-stimulated genes (ISGs) according to the Interferome database^43^ (Fig. 2A). Of the top 20 most significantly downregulated protein-coding genes, 18 were ISGs, with the exception of *Apol9b* and *Zfp781* (Fig. 2B). Gene set enrichment analysis (GSEA) further confirmed that PCBP1 depletion impairs type I IFN-related gene sets, including those associated with IFN production and downstream response (Fig. 2C, D).

The downregulation of ISG expression, including *Isg15*, *Irf7*, *Ifit1*, *Rsad2*, and *Bst2*, as well as *Ifnb1*, was validated by qPCR in PyMT *Pcbp1*–/– tumors (Fig. 2E). Immunoblotting further confirmed the decreased expression of ISG15 and IRF7 proteins, and phosphorylated STAT1 (p-STAT1) levels, indicating attenuated type I IFN signaling since p-STAT1 is a direct downstream effector of IFNAR activation by type I IFNs (Fig. 2F). Consistently, ISG15 protein expression was significantly diminished in tumor cells by immunohistochemistry (Fig. 2G, H).

To evaluate the relevance of our findings in human breast cancer, we performed correlation analyses using the RNA-sequencing data from 1247 primary breast tumors in The Cancer Genome Atlas (TCGA BRCA) dataset that previously served to generate the Kaplan–Meier curves (Fig. 1A). *ISG15* and *IRF3* expression showed a strong positive correlation (*r* = 0.4424, *p* = 1.63 × 10^−59^), serving as a positive control given that IRF3 promotes the transcription of ISGs (Fig. 2I, J). *PCBP1* expression positively correlated with several key ISGs, including *IRF3* (*r* = 0.2691, *p* = 1.17 × 10^−21^), *ISG15* (*r* = 0.2449, *p* = 4.36 × 10^−18^), and, to a lesser extent, *IRF7* (*r* = 0.1458, *p* = 3.19 × 10^−7^) (Fig. 2I, J), suggesting a conserved relationship between PCBP1 and type I IFN signaling in human breast tumors. These results underscore the clinical relevance of our mouse model findings and support the hypothesis that PCBP1 promotes type I IFN signaling in both murine and human breast cancer.

**Fig. 3:**
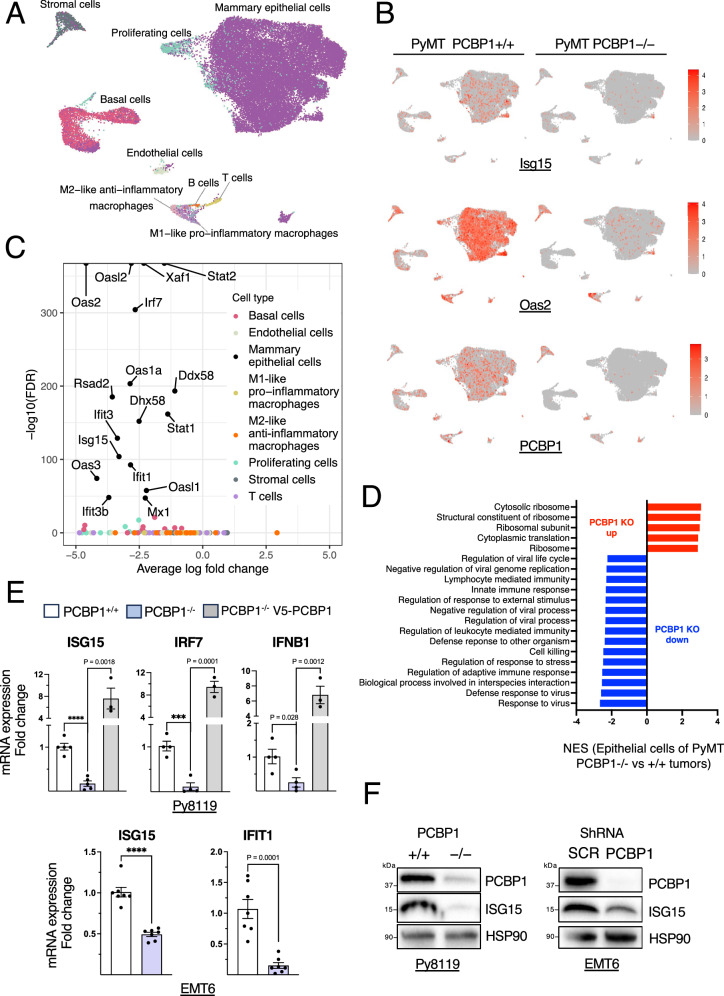
PCBP1 enhances type I IFNs signaling in breast cancer cells. **A** UMAP plot from snRNA-seq analysis showing the different annotated cell type clusters in PyMT *Pcbp1*+/+ (*n* = 2) and PyMT *Pcbp1*–/– (*n* = 2) mammary tumors. **B** UMAP plot representing the expression of *Pcbp1, Isg15* and *Oas2* in the annotated cell types in pooled PyMT *Pcbp1*+/+ and PyMT *Pcbp1–/–* groups. **C** Volcano plot of the average log fold change versus –log10(FDR) for interferon-stimulated genes differentially expressed across all the annotated cell types from the snRNA-seq analysis. **D** GSEA analysis with M5 (ontology), based on single nuclei RNA-sequencing results, representing the normalized enrichment score of significantly upregulated and downregulated gene sets specifically in the mammary epithelial cells of PyMT *Pcbp1*–/– versus PyMT *Pcbp1*+/+ tumors. **E** RT-qPCR analysis of *Isg15, Irf7, Ifit1* and *Ifnb1* expression in Py8119 *Pcbp1*+/+ (*n* = 5), *Pcbp1*–/– (*n* = 5) and *Pcbp1*–/– rescued with V5-tagged *PCBP1* (*n* = 3), as well as in EMT6 shScramble (*n* = 7) versus sh*PCBP1* cells (*n* = 7). **F** Immunoblot analysis of PCBP1, ISG15 and HSP90 levels in Py8119 and EMT6 shScramble versus sh*PCBP1* cell lines. Data are represented as mean ± SEM, two-tailed unpaired *t* test.

We then determined the specific cell type(s) responsible for the reduced type I IFN response in *Pcbp1*-deficient tumors by performing a single-nuclei RNA-sequencing (snRNA-seq). Clustering analysis and cell-type annotation based on marker gene expression (Supplementary Fig. 1A) revealed distinct tumor cell populations (Fig. 3A, Supplementary Table 2). We next assessed ISG expression within these populations. A UMAP representation showed impaired expression of *Oas2*, the most significantly downregulated ISG in PyMT *Pcbp1*−/− tumors by snRNA-seq, and of *Isg15*, both predominantly within mammary epithelial cells (Fig. 3B). Additional bioinformatics analysis confirmed that a set of ISGs was significantly downregulated only in *Pcbp1*-silenced mammary epithelial cells, and not in other cell types, based on FDR and fold change (Fig. 3C). This is largely because these tumor cells are by far the most abundant population (~80% of the samples; Supplementary Table 2). By contrast, cell-type–resolved analysis shows that ISGs are also modulated in additional, less abundant lineages, such as macrophages (Supplementary Fig. 1B), consistent with paracrine type I IFN signaling in the tumor microenvironment. These effects are masked in bulk analysis due to the rarity of non-epithelial cells.

GSEA of differentially expressed genes in epithelial cells further supported the impairment of innate immune responses and viral defense programs in *Pcbp1*-deficient cancer cells (Fig. 3D), consistent with the bulk RNA-seq results (Fig. 2C, D).

We next validated these findings in vitro. In Py8119 mesenchymal-like mouse triple-negative breast cancer cells, CRISPR-Cas9-mediated knockout of *Pcbp1* led to a marked reduction in *Isg15, Irf7*, and *Ifnb1* mRNA levels compared to control cells (Fig. 3E). Overexpression of *Pcbp1* in knockout cells (Supplementary Fig. 1C) restored and even enhanced their expression relative to wild-type levels, confirming PCBP1 as a strong inducer of interferon-stimulated genes and *Ifnb1* transcription. Similar results were obtained in the EMT6 breast cancer cell line using shRNA-mediated *Pcbp1* silencing, with significant downregulation of *Isg15 and Ifit1* (Fig. 3E). Decreased ISG15 protein levels upon PCBP1 loss was also confirmed across both cell lines (Fig. 3F). Notably, *Pcbp1* knockout in Py8119 cells is only partial (Fig. 3F), as a complete loss may impair cell survival, consistent with the embryonic lethality observed in *Pcbp1*-null mice^44^. Nevertheless, even partial loss was sufficient to significantly reduce ISG expression (Fig. 3E, F).

### PCBP1 induces CXCL9/10, MHC-I expression in mammary epithelial cells and intratumoral cytotoxic T cell infiltration

**Fig. 4:**
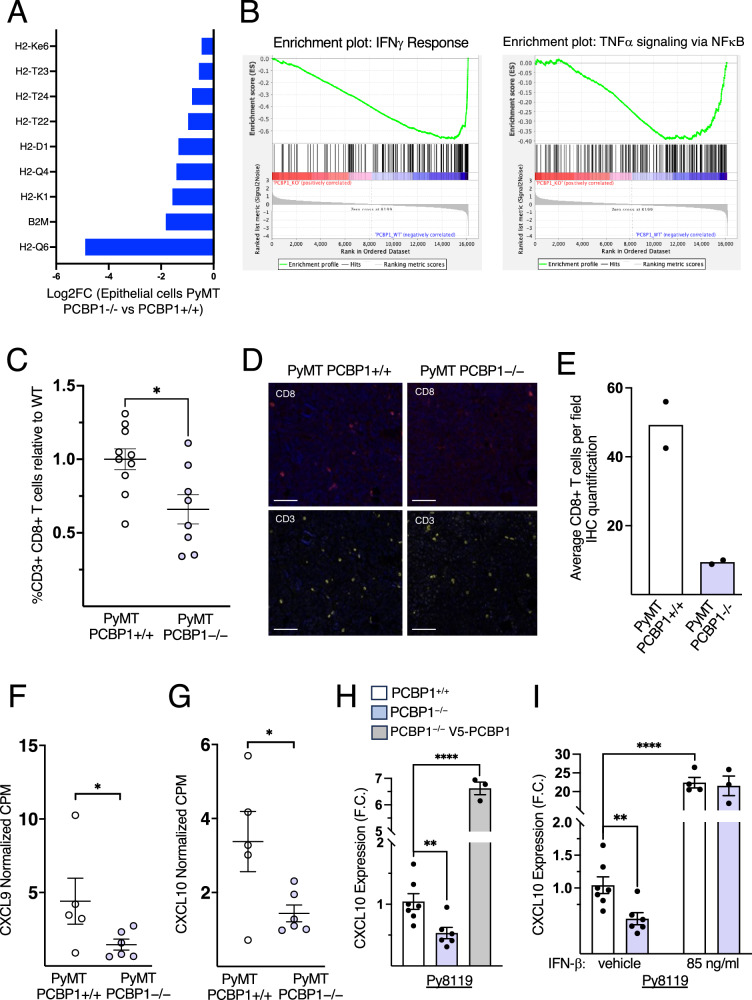
PCBP1 induces *Cxcl9, Cxcl10* and MHC class I expression in cancer cells and CD3+CD8+ cytotoxic T-cell infiltration in mammary tumors. **A** Single-nuclei RNA-seq analysis representing the Log2FC expression of significantly regulated MHC class I molecules and beta-2 microglobulin (*B2m*) in PyMT *Pcbp1*–/– mammary epithelial cells (*n* = 2) compared to PyMT *Pcbp1*+/+ (*n* = 2). **B** Enrichment plots of ‘Interferon Gamma Response’ (NES = −3.06, FDR *q* value < 0.0001) and ‘TNFalpha Signaling via NFkappaB’ (NES = −1.82, FDR *q* value = 0.0011) gene sets from GSEA analysis with MSigDB Hallmark (**H**) based on bulk RNA-seq data from PyMT *Pcbp1*–/– vs PyMT *Pcbp1*+/+ mammary tumors. **C** Cytotoxic T-cell infiltration analysis in PyMT *Pcbp1*+/+ (*n* = 10) versus PyMT *Pcbp1*–/– (*n* = 8) tumors by flow cytometry using the CD3 and CD8 markers. **D** Dual immunohistofluorescence of CD3 and CD8 markers in *Pcbp1*+/+ (*n* = 2) vs. PyMT *PCBP1*–/– (*n* = 2) tumors with (**E**). Its quantification (three fields counted per sample, scale bars: 100 µm). **F** *Cxcl9* and **G** *Cxcl10* expression in normalized count per million from bulk RNA-sequencing analysis in PyMT *Pcbp1*+/+ (*n* = 5) vs PyMT *Pcbp1*–/– (*n* = 6) tumors. **H** RT-qPCR analysis of *Cxcl10* expression (fold change) in Py8119 *Pcbp1*−/− (*n* = 6) and *Pcbp1*−/− rescued with V5-*PCBP1* WT (*n* = 3) relative to Py8119 *Pcbp1*+/+ (*n* = 7). **I** RT-qPCR analysis of *Cxcl10* in Py8119 *Pcbp1*−/− (*n* = 6) vs Py8119 *Pcbp1* + /+ (*n* = 7) following vehicle or 85 ng/mL IFN-β for 12 h. Data are represented as mean ± SEM, two-tailed unpaired *t* test.

Type I interferons in the tumor microenvironment are well known to upregulate MHC class I expression on cancer cells, facilitating the presentation of tumor-specific antigens and the activation of cytotoxic T cells. This can occur either directly or via cross-priming by dendritic cells, which are themselves activated by type I IFNs and further support CD8 + T-cell infiltration and cytotoxic responses against tumor cells^4,45–48^. Consistent with impaired antigen presentation upon *Pcbp1* loss, snRNA-seq showed decreased expression of classical and non-classical MHC class I genes, as well as *B2m*, which encodes beta-2 microglobulin, a structural component required for MHC-I surface stability, in mammary epithelial cells of *Pcbp1*-silenced tumors (Fig. 4A).

Given PCBP1’s role in promoting type I IFN signaling, we hypothesized that its loss could reshape the tumor immune microenvironment, particularly T-cell infiltration. GSEA revealed significant negative enrichment of the IFN-γ response and TNF-α signaling via NF-κB (Fig. 4B), two pathways commonly driven by inflammatory cells, especially activated CD8⁺ T cells. Moreover, snRNA-seq indicated reduced T-cell populations in Pcbp1-deficient tumors (Supplementary Table 2). These findings supported the hypothesis that PCBP1 loss compromises antigen presentation and immune cell infiltration, particularly that of cytotoxic T cells. Strikingly, both flow cytometry and dual immunofluorescence confirmed a significant reduction in tumor-infiltrating CD3⁺CD8⁺ cytotoxic T cells upon *Pcbp1* silencing (Fig. 4C–E; Supplementary Fig. 2).

This decrease in cytotoxic T cells was associated with reduced *Cxcl9 and Cxcl10 t*ranscripts in *Pcbp1*-deficient tumors by bulk RNA-seq (Fig. 4F–G), two chemokines that promote intratumoral CD8⁺ T-cell recruitment. In vitro, RT-qPCR in Py8119 cells confirmed that partial *Pcbp1* knockout lowers *Cxcl10*, whereas re-expression of V5-PCBP1 (Supplementary Fig. 1C) restores, and exceeds, wild-type levels (Fig. 4H). Treatment of *Pcbp1* knockout cells with IFN-β rescued *Cxcl10* expression, indicating an IFN-β-mediated effect (Fig. 4I).

Together, these data indicate that *Pcbp1* silencing diminishes MHC class I and IFN-β–driven *Cxcl10* expression in tumor cells and impairs CD8⁺ cytotoxic T-cell infiltration, contributing to a colder, less immunogenic tumor microenvironment.

### PCBP1 promotes cGAS-STING pathway activation in mammary epithelial cells via its single-stranded nucleic acid-binding activity

**Fig. 5:**
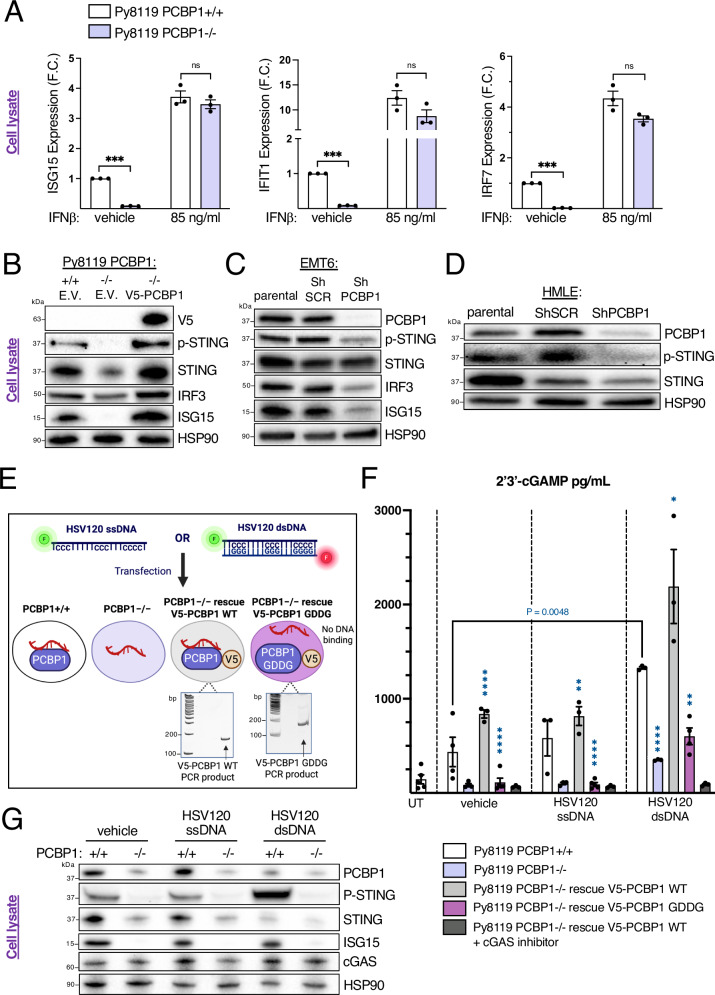
PCBP1’s single-stranded nucleic acid-binding abilities increase cGAS activity and downstream STING signaling activation in mouse and human mammary cells. **A** RT-qPCR analysis of *Isg15, Irf7* and *Ifit1* in Py8119 *Pcbp1*+/+ and *Pcbp1*–/– cells after vehicle or 85 ng/mL IFN-β treatment (mean ± SEM, two-tailed unpaired *t* test). **B** Immunoblot of cGAS-STING-type I IFN activation marker: p-STING, total STING, two ISGs: IRF3, ISG15, as well as HSP90 and V5, in Py8119 *Pcbp1*+/+ cells vs *Pcbp1*–/– or *Pcbp1*–/– rescued with V5-tagged WT *PCBP1*. **C** Immunoblot of the indicated markers in parental, shScramble and sh*PCBP1* EMT6 mouse breast cancer cell line or (**D**) in HMLE human mammary epithelial cells. **E** Representation of four cell lines generated: Py8119 *Pcbp1*+/+, *Pcbp1*–/–, and *Pcbp1*–/– cells rescued with either V5-tagged wild-type *PCBP1* or a V5-tagged *PCBP1* mutant in which all KH domain GxxG loops were mutated to GDDG to impair single-stranded nucleic acid binding. Expression of the V5-PCBP1 WT and GDDG-mutant constructs was confirmed by RT-PCR using primers targeting the wild-type or GDDG-mutant KH3 domain of *Pcbp1* in forward and the V5 tag in reverse. Cells were then transfected with either a fluorescently labeled single-stranded (ssDNA) or double-stranded (dsDNA) HSV120 oligonucleotide containing poly-cytosine motifs. **F** cGAS activity assay measuring 2’3’-cGAMP production in cell lysates by ELISA (mean ± SEM, two-tailed unpaired *t* test, asterisks indicate statistical comparisons between each bar and the immediately preceding condition). **G** as well as its associated western blot on these same samples for the indicated markers after treatment with vehicle (lipofectamine 3000 only) or 1.5 µg of HSV120 ssDNA or 1.5 µg of HSV120 dsDNA +/− cGAS inhibitor (TDI-6570) for 20 hours.

To better define the molecular mechanism through which PCBP1 exerts its effects, we investigated whether it acts upstream of IFNAR signaling in mammary epithelial cells by treating Py8119 *Pcbp1*+/+ and *Pcbp1*–/– cells with IFN-β. Exogenous IFN-β rescued ISG expression in *Pcbp1*–/– cells, indicating that PCBP1 functions upstream of IFNAR (Fig. 5A). A previous study by Liao et al.^49^ reported that PCBP1 can be found associated with cGAS following viral infections; however, this had not been demonstrated in the context of cancer or validated in vivo, and the regulation mechanism remains unclear. We therefore examined cGAS-STING activation markers. Immunoblot analysis revealed that PCBP1 loss reduced levels of p-STING, IRF3, ISG15 (Fig. 5B), and p-TBK1 (Supplementary Fig. 4A) in both mouse Py8119 and EMT6 cells (Fig. 5C), without altering cGAS expression (Supplementary Fig. 4B, Fig. 5G). Consistently, decreased p-STING was also observed in human mammary epithelial HMLE cells (Fig. 5D), suggesting impaired basal cGAS-STING activation. Restoring V5-tagged WT PCBP1 in *Pcbp1*–/– Py8119 cells fully rescued pathway activation, as indicated by restored p-STING levels, whereas total STING abundance was inconsistently affected by PCBP1 loss across cell lines, suggesting that PCBP1 primarily regulates STING activation rather than its expression (Fig. 5B).

Because both PCBP1 and cGAS are nucleic acid-binding proteins, we investigated whether PCBP1 could promote cGAS activation and 2′3′-cGAMP production and whether this function depends on PCBP1’s ability to bind single-stranded nucleic acids. To do so, we generated a PCBP1 mutant in which the conserved GxxG motif in all three KH domains was mutated to GDDG. This motif forms part of the KH domain’s single-stranded DNA/RNA-binding cleft by accommodating the phosphate backbone; introducing aspartates disrupts binding without affecting protein folding^50^. Both WT and GDDG constructs were expressed in Py8119 *Pcbp1*–/– cells (Fig. 5E). Cells were then transfected with fluorescently labeled HSV120 ssDNA or dsDNA containing poly-cytosine motifs (Fig. 5E). The same transfection efficiency was observed between *Pcbp1*-modulated cell lines (Supplementary Fig. 3).

While dsDNA strongly induced 2’3’-cGAMP production (Fig. 5F) and cGAS-STING activation as observed by increased P-STING levels (Fig. 5G) in Pcbp1-proficient cells, ssDNA failed to do so, consistent with its limited ability to activate cGAS^51^. Importantly, PCBP1 loss impaired 2’3’-cGAMP production (Fig. 5F) and STING activation (Fig. 5G) not only at baseline but also after dsDNA transfection. Additionally, while re-expression of V5-PCBP1 WT significantly increased 2′3′-cGAMP production in *Pcbp1*–/– cells, even upon dsDNA transfection that PCBP1’s KH domains were not expected to bind, the GDDG-mutant or treatment with a cGAS inhibitor abolished the rescue by V5-PCBP1 WT (Fig. 5F). This confirms that the increase in 2’3’-cGAMP production is dependent on both PCBP1’s single stranded nucleic acid-binding ability and cGAS enzymatic activity (Fig. 5F). However, a dsDNA dose–response revealed that the PCBP1-dependent increase in 2′3′-cGAMP occurs only at basal and low DNA inputs and is absent at higher dsDNA doses (Supplementary Fig. 4C), suggesting that PCBP1 may influence cGAS activity when lower amount of DNA is present in the cytoplasm.

*Pcbp1* silencing also reduced 2’3’-cGAMP levels in HMLE cells (Supplementary Fig. 4D). In addition, decitabine, a DNMT inhibitor known as a cytoplasmic nucleic acids inducer and activator of the pathway^52–54^, triggered p-STING and ISG15 upregulation in *Pcbp1*-proficient Py8119 and EMT6 cells, but this response was blunted in *Pcbp1*-deficient cells (Supplementary Fig. 4E–H).

These findings show that PCBP1 enhances cGAS activation through its single-stranded DNA-binding KH domains, thereby promoting STING-dependent type I IFN signaling in mammary epithelial cells.

### PCBP1 and cGAS simultaneously bind poly-cytosine single-stranded nucleic acids

Given that PCBP1’s ability to bind single-stranded nucleic acids is critical for cGAS activation in mammary cells and that this effect persists even with dsDNA administration, we investigated whether PCBP1 and cGAS can simultaneously interact with the same single-stranded molecule, and whether PCBP1 binds dsDNA.

**Fig. 6:**
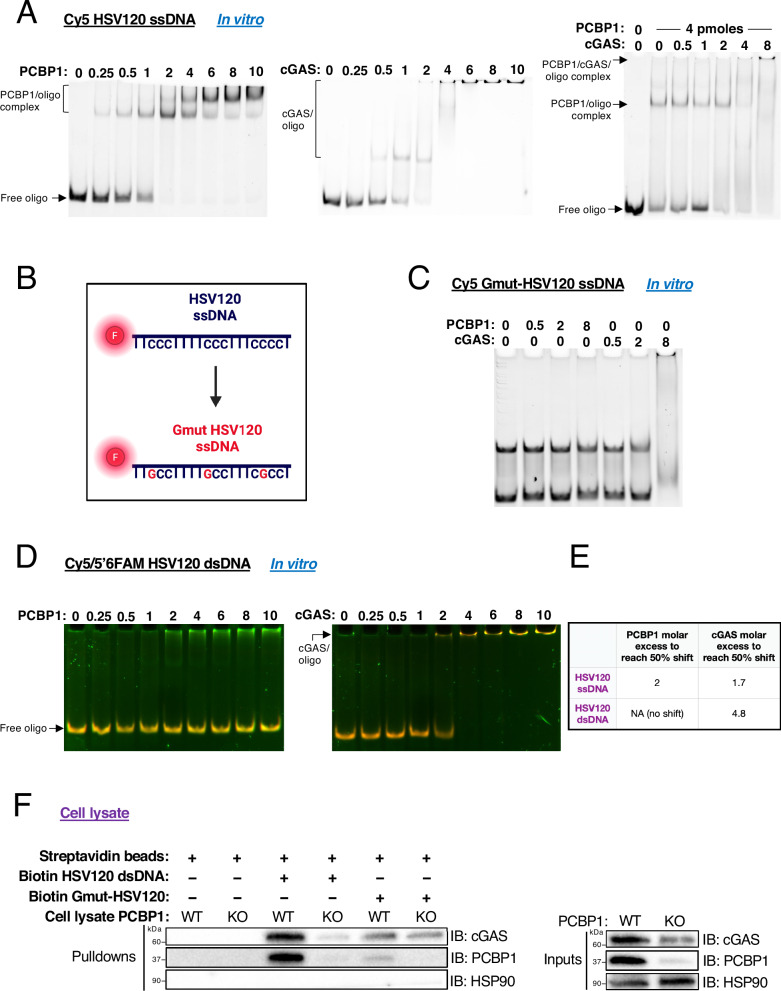
PCBP1 and cGAS simultaneously bind poly-cytosine tracts. **A** EMSA showing human PCBP1 (left) or human cGAS (center) or both proteins (right) binding to the single-stranded poly-cytosine HSV120 ssDNA tagged with Cyanine 5 (units are in pmoles). **B** Representation of the Cyanine 5-tagged Gmut-HSV120 ssDNA oligo used in the following EMSA, where the first cytosines of the polyC tracts have been mutated to guanines. **C** EMSA showing human PCBP1 or human cGAS binding to the Gmut-HSV120 ssDNA tagged with Cyanine 5. **D** EMSA showing human PCBP1 (left) or human cGAS binding (right) to the double-stranded poly-cytosine HSV120 dsDNA tagged with Cyanine 5 in forward and 5’6’FAM in reverse. **E** EMSA quantification **F** Streptavidin pulldown of biotinylated HSV120 dsDNA versus Gmut-HSV120 dsDNA oligos in *Pcbp1*+/+ and –/– lysates followed by immunoblotting for cGAS, PCBP1 and HSP90 as a negative control in the pulldown and loading control in the input.

We first performed electrophoretic mobility shift assays (EMSAs) using Cyanine 5-labeled HSV120 single-stranded DNA (Fig. 6B). PCBP1 bound HSV120 ssDNA with 50% shift estimated at 2× molar excess (Fig. 6A left panel, 6E), and cGAS bound similarly with a 50% shift at 1.7× excess (Fig. 6A middle panel, 6E). When cGAS was titrated in the presence of a constant amount of PCBP1, the PCBP1/oligo complex disappeared, and a super-shifted complex appeared, consistent with simultaneous binding of both proteins to the same ssDNA molecule (Fig. 6A right panel).

To assess sequence specificity, we mutated the first cytosine in each polyC tract of HSV120 to generate Gmut-HSV120 ssDNA (Fig. 6B). PCBP1 lost binding to the mutant, whereas cGAS showed no sequence specificity (Fig. 6C), consistent with its known interaction with the phosphate-sugar backbone rather than specific bases^1–3^. We further tested a single-stranded RNA control, the BAT element (BAT RNA), a 3′-UTR motif containing consecutive cytidine (rCrCrC) repeats previously shown to bind PCBP1 and regulate mRNA translation^33^. The same results were obtained for PCBP1 and cGAS binding using this BAT RNA sequence and its G-mutant counterpart (Supplementary Fig. 5A–C).

We next tested binding to HSV120 dsDNA formed by annealing Cy5-labeled forward and 5’6-FAM-labeled reverse strands. cGAS bound dsDNA with a 50% shift estimated at ~4.8× molar excess. In contrast, PCBP1 showed no binding (Fig. 6D, E). Gradient PAGE confirmed complete annealing, with no residual ssDNA (Supplementary Fig. 5D) and the same dsDNA shift was observed in the presence of cGAS, whereas PCBP1 had no effect (Supplementary Fig. 5E).

To reconcile these findings with the observed PCBP1-dependent effects on cGAS activation upon dsDNA transfection (Fig. 5F), we performed pull-down assays using biotinylated HSV120 dsDNA and whole-cell lysates from Py8119 *Pcbp1* WT or KO cells. Surprisingly, PCBP1 was robustly pulled down with HSV120 dsDNA, but not with its G-mutant counterpart (Fig. 6F), suggesting that, in a cellular context, PCBP1 may access polyC-containing single-stranded regions within dsDNA, possibly through interactions with protein partners. Consistently, cGAS binding to WT HSV120 dsDNA was reduced in *Pcbp1*-deficient lysates, indicating that PCBP1 facilitates cGAS recruitment. While cGAS could bind both the WT and mutant oligos, its association with the WT was enhanced in the presence of PCBP1, whereas binding to the mutant remained low regardless of PCBP1 status, indicating that PCBP1 promotes cGAS recruitment in a sequence-dependent manner (Fig. 6F). Similar results were obtained using BAT RNA and its G-mutant variant (Supplementary Fig. 6A).

Together, these findings show that PCBP1 specifically binds polyC-containing single-stranded nucleic acids and can co-bind these sequences with cGAS in vitro. In cells, PCBP1 may facilitate cGAS association with DNA by recognizing transiently exposed single-stranded polyC motifs within dsDNA, explaining the increased 2’3’-cGAMP production observed in the presence of PCBP1.

### PCBP1 binding to single-stranded poly-cytosine motifs increases cGAS affinity for nucleic acids and promotes 2’3’-cGAMP production

**Fig. 7:**
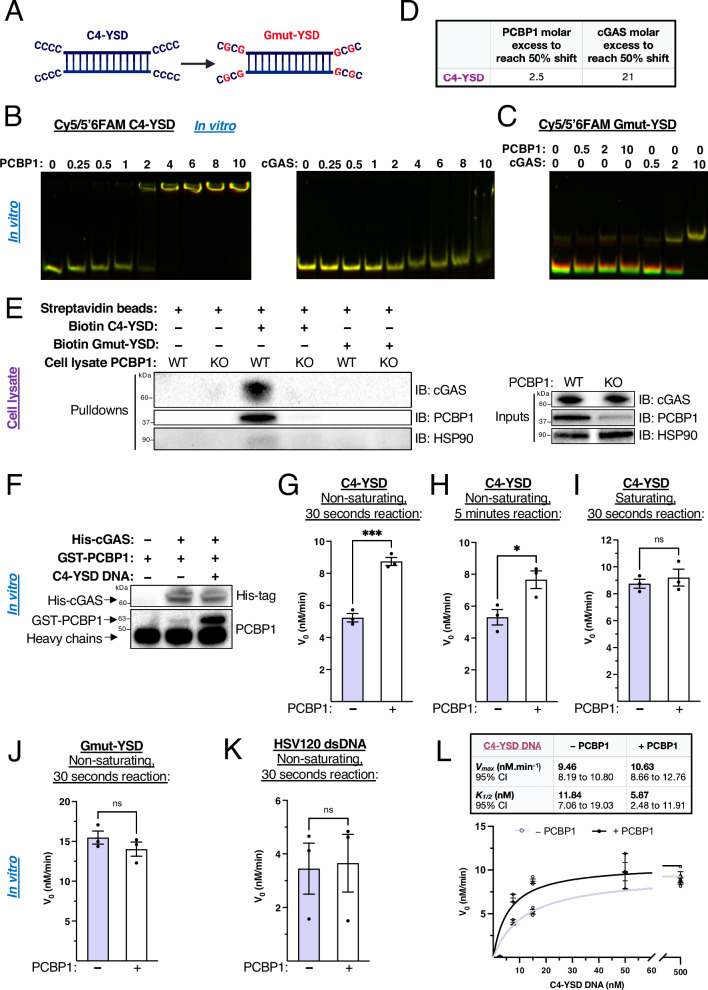
PCBP1 binding to poly-cytosine single-stranded motifs enhances cGAS affinity for these nucleic acids and increases 2’3’-cGAMP production efficiency under sub-saturating conditions in vitro. **A** Schematic representation of C4-YSD (poly-C-rich) and Gmut-YSD (cytosines mutated to guanines) DNA duplexes. **B** Electrophoretic mobility shift assays showing in vitro binding of increasing concentrations of recombinant human PCBP1 (left) or human cGAS (right) to Cy5/5′6-FAM-labeled C4-YSD DNA (units are in pmoles). **C** EMSA showing in vitro binding of increasing concentrations of recombinant human PCBP1 or human cGAS to Cy5/5′6-FAM-labeled Gmut-YSD DNA. **D** EMSA quantification. **E** Streptavidin pulldown assays from Py8119 Pcbp1 WT or Pcbp1 KO cell lysates incubated with biotin-labeled C4-YSD or Gmut-YSD followed by immunoblotting for cGAS, PCBP1 and HSP90 as a negative control in the pulldown and loading control in the input. **F** In vitro co-immunoprecipitation of recombinant human His-cGAS in the presence of recombinant human GST-PCBP1 and with or without C4-YSD followed by His-tag and PCBP1 immunoblotting. **G**–**I** Human cGAS (30 nM) enzymatic activity (initial velocity, *v*₀) measured in 30 seconds or 5 minutes reactions using sub-saturating (15 nM) or saturating (500 nM) C4-YSD concentrations in the absence or presence of human PCBP1 (60 nM). **J** Human cGAS (30 nM) enzymatic activity (initial velocity, *v*₀) measured in 30 seconds using sub-saturating Gmut-YSD concentrations (15 nM, **K**) or using sub-saturating HSV120 dsDNA concentrations (15 nM) in the absence or presence of human PCBP1 (60 nM). **L** Michaelis–Menten kinetics of human cGAS activity (30 nM) using increasing concentrations of C4-YSD DNA in the absence or presence of human PCBP1 (60 nM). Data are represented as mean ± SEM, two-tailed unpaired *t* test.

To better understand the mechanism by which PCBP1 modulates cGAS affinity for polyC-containing single-stranded DNA, we engineered a variant of the G3-YSD oligo, a Y-shaped cGAS agonist^28^, with poly-cytosine single-stranded overhangs (C4-YSD) by replacing G-triplets with C4 motifs (Fig. 7A). A control sequence (Gmut-YSD) with disrupted polyC tracts was also generated.

EMSAs revealed that PCBP1 binds C4-YSD with higher affinity than cGAS (50% shift estimated at 2.5× vs 21× molar excess, Fig. 7B, D). While cGAS bound both C4-YSD and Gmut-YSD similarly, PCBP1 binding was abolished by polyC mutations (Fig. 7C), confirming its strict sequence specificity.

Using pull-down assays in whole-cell lysates, we observed enhanced cGAS binding to C4-YSD in the presence of PCBP1, which was lost when using the PCBP1 GDDG-mutant or the Gmut-YSD oligo (Fig. 7E, Supplementary Fig. 6B). These results confirm that PCBP1 increases cGAS association with polyC-containing DNA through its KH domain-dependent RNA/DNA-binding.

Co-immunoprecipitation of recombinant PCBP1 and cGAS showed complex formation only in the presence of the single-stranded polyC DNA in vitro (Fig. 7F), indicating that their association is mediated through co-binding to nucleic acids rather than direct protein-protein interaction. Thus, PCBP1 is not a classical allosteric activator of cGAS but a sequence-specific DNA co-sensor.

To functionally test this model, we performed cGAS enzyme kinetics measured by a 2’3’-cGAMP ELISA-based assay using C4-YSD, HSV120 dsDNA, or their respective G-mutant variants under both saturating (DNA in excess) and non-saturating conditions (cGAS in twofold excess over DNA), in the presence or absence of PCBP1. Under non-saturating conditions, PCBP1 significantly increased the initial rate of 2’3’-cGAMP production with C4-YSD after both 30 seconds (Fig. 7G, Supplementary Tables 3) and 5 minutes (Fig. 7H, Supplementary Tables 3). This effect was lost under saturating conditions (Fig. 7I, Supplementary Tables 3), indicating that PCBP1 enhances the binding of cGAS to nucleic acids rather than increasing its catalytic turnover. When DNA is abundant, cGAS can be fully bound, and PCBP1 has no further effect.

Consistent with its sequence specificity, PCBP1 had no effect when using the Gmut-YSD (Fig. 7J, Supplementary Tables 3) or polyC HSV120 blunt-ended dsDNA (Fig. 7K, Supplementary Tables 3), both of which it cannot bind in vitro. Finally, Michaelis-Menten analysis^55,56^ using increasing concentrations of C4-YSD revealed that PCBP1 decreases by twofold the *K*_*1/2*_ of cGAS, which corresponds to the substrate concentration required to reach half of the maximal enzymatic velocity and reflects the apparent DNA-binding affinity. However, PCBP1 does not alter *V*_max_, confirming that it increases the apparent affinity of cGAS for single-stranded polyC-containing DNA without affecting its intrinsic catalytic rate (*k*_*cat*_) (Fig. 7L).

This also confirms the results observed in Py8119 cells where PCBP1 increased 2’3’-cGAMP production only at basal or lower amount of transfected polyC HSV120 blunt-ended dsDNA (Supplementary Fig. 4A). PCBP1 is able to bind this type of polyC dsDNA only in a cellular context and its activity is not required when cytoplasmic dsDNA is too abundant in the cytoplasm, mimicking saturating concentrations in our enzyme kinetics assays (Fig. 7L).

**Fig. 8:**
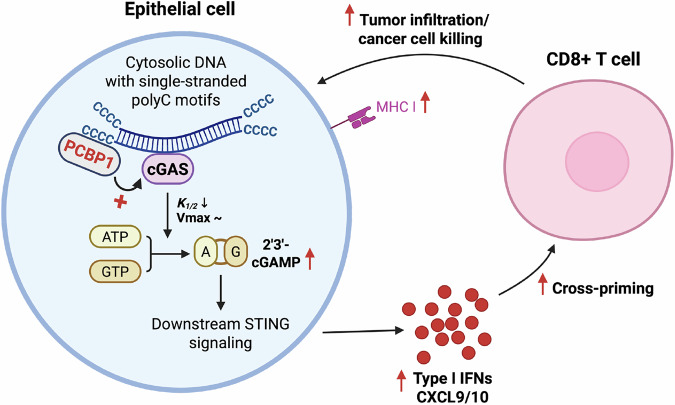
Schematic model of PCBP1-mediated amplification of cGAS-STING signaling in mammary epithelial cells. PCBP1 binds cytosolic DNA containing accessible single-stranded poly-cytosine motifs and increases cGAS affinity for these nucleic acids, resulting in reduced *K*_1/2_ and enhanced production of 2’3’-cGAMP at sub-saturating DNA concentrations. Elevated 2’3’-cGAMP drives downstream STING activation, leading to increased type I interferon and chemokine (CXCL9/10) secretion. This promotes CD8⁺ T-cell infiltration, enhances MHC-I expression on tumor cells, and facilitates tumor cell killing, thereby impairing tumorigenesis. Created in BioRender. Frereux, C. (2025) https://BioRender.com/hvrezkp.

Together, these results demonstrate that PCBP1, by binding to single-stranded poly-cytosine motifs, increases the affinity of cGAS for these nucleic acids and makes 2’3’-cGAMP production more efficient under sub-saturating conditions. This positions PCBP1 as a DNA co-sensor, providing the base-sequence specificity that cGAS lacks to amplify the cGAS-STING/type I IFN/ISG response when poly-cytosine nucleic acids are present in the cytoplasm. In turn, this amplifies type I IFN and chemokine secretion in the tumor microenvironment, increases MHC-I expression on cancer cells, recruits cytotoxic T cells to the tumor site, and impairs tumorigenesis (Fig. 8).

## Discussion

In this study, we demonstrate that PCBP1 acts as a molecular enhancer of cGAS-STING pathway activation in both human and mouse mammary epithelial cells, promoting type I interferon production and downstream interferon-stimulated gene (ISG) expression. Mechanistically, PCBP1 increases the affinity of cGAS for nucleic acids containing single-stranded poly-cytosine motifs, resulting in enhanced 2′3′-cGAMP production under sub-saturating conditions. This leads to increased type I IFN, ISG and chemokines expression that ultimately supports CD8 + T cell infiltration in the tumor microenvironment, contributing to reduced tumor burden.

The increased CD8+ cytotoxic T cell infiltration observed in PyMT *Pcbp1*-proficient tumors seems to be a downstream consequence of PCBP1-mediated activation of the cGAS-STING pathway and subsequent type I IFN, CXCL9/10 production. It is well established that type I IFNs enhance MHC class I presentation and CXCL9/10 secretion to recruit CD8 + T cells to the tumor microenvironment^4,13–15^. However, other immune-regulatory mechanisms may also contribute. For instance, our group previously showed that PCBP1 silencing induces EMT and upregulates *IGSF11/VSIG3*^57^, a ligand for the checkpoint receptor VISTA, which transmits inhibitory signals to MDSCs and T cells, dampening their proliferation and cytokine production^58^. This suggests that PCBP1 may orchestrate a broader immune-regulatory program, simultaneously promoting antigen presentation and relieving T cell suppression.

Interestingly, snRNA-seq also revealed an increased proportion of basal cells in *Pcbp1*-deficient tumors (10.7% vs 4.2% in controls; Supplementary Table 2). Such a basal expansion can be linked to an enrichment of basal-like/EMT programs and lineage malignant plasticity (luminal-to-basal transition), features repeatedly tied to increased aggressiveness and progression^59–61^.

On the mechanistic aspect, although our assays focused on specific substrates, PCBP1 likely enhances cGAS sensing of a broader range of endogenous and exogenous nucleic acids, provided that single-stranded poly-cytosine tracts are present or exposed. Given that PCBP1 fails to bind dsDNA in vitro but does so in whole-cell lysates, we propose that additional cellular factors facilitate local DNA unwinding to reveal polyC-rich single-stranded regions. This is particularly relevant in cancer cells, where cytoplasmic DNA is unlikely to be uniformly blunt-ended and may instead harbor single-stranded overhangs or undergo structural remodeling. We previously identified PCBP1’s top interactors to be a group of helicases from the DExD/H box family, some of which are already known as regulators of the cGAS-STING pathway^62,63^. This includes the helicase DHX9, whose interaction with PCBP1 was confirmed by co-immunoprecipitation in A549 cells^64^ and in this study in Py8119 cells (Supplementary Fig. 5D). DHX9 was also part of the complex with PCBP1, cGAS and the HSV120 dsDNA (Supplementary Fig. 5C). These interacting partners could participate in the remodeling of DNA to reveal accessible single-stranded polyC motifs, but this remains hypothetic and requires further investigation.

PCBP1 increased the initial rate of 2’3’-cGAMP production by ~3.51 nM/min under sub-saturating single-stranded polyC substrate conditions (Fig. 7G, Supplementary Table 2). Given the reported *Kd* of STING for 2’3’-cGAMP is 3.79nM^65^, which represents a high-affinity interaction, even small increases in 2’3’-cGAMP can trigger strong downstream signaling. Thus, PCBP1 is expected to rapidly raise 2’3’-cGAMP levels beyond the STING activation threshold, enhancing type I IFN signaling under low substrate availability. These findings highlight PCBP1 as a key co-regulator of efficient DNA sensing and cGAS-STING activation.

Importantly, human and mouse PCBP1 have the same protein sequence, and PCBP1 alone is sufficient to increase human cGAS affinity for polyC-containing nucleic acids in a fully reconstituted in vitro system. This suggests that this mechanism may be conserved across different cell types expressing both PCBP1 and cGAS, and likely not limited to human and mouse mammary cells. This broadens the relevance of our findings to various physiological and pathological contexts.

From a functional standpoint, enhancing the efficiency of cGAS in sensing polyC-rich nucleic acids could have important implications in cancer. In tumor cells undergoing alternative lengthening of telomeres (ALT), extrachromosomal telomeric repeat (ECTR) DNA can accumulate in the cytoplasm and has been shown to trigger cGAS-STING^66^. These telomeric sequences are G-rich on one strand and C-rich on the complementary strand, which could potentially allow PCBP1 to recognize and amplify cGAS activation. Additionally, in the context of viral infections, several viral genomes contain polyC tracts^67–69^, and PCBP1 may function to enhance immune detection of these pathogens by facilitating cGAS binding and activation.

In our study, we used transfected DNA oligos and an engineered C4-YSD construct as defined experimental tools to dissect the molecular mechanism of PCBP1-mediated cGAS activation. However, future work will be needed to identify the endogenous sources and types of polyC-containing DNA on which PCBP1 may enhance cGAS sensing in a physiological cellular context.

In summary, our findings reveal that PCBP1 functions as a sequence-specific co-sensor that guides cGAS toward single-stranded poly-cytosine–containing nucleic acids. To our knowledge, this is the first demonstration that a single-stranded nucleic acid-binding protein can confer base composition–dependent specificity to cGAS sensing, unveiling a previously unrecognized layer of regulation in innate DNA recognition with important implications for tumor suppression.

## Materials and methods

### Animal studies

All animal procedures have been approved by the Animal Care and Use Committees of the Medical University of South Carolina. We have complied with all relevant ethical regulations for animal use.

Mice were maintained under standard specific-pathogen-free conditions and group-housed (maximum five per cage) with *ad libitum* access to chow and water and standard environmental enrichment. The C57BL/6 MMTV-PyMT MMTV-Cre *Pcbp1*(fl/fl) triple transgenic mouse model, as well as the primers used for genotyping, were previously described^70^.

Briefly, this line was generated by crossing C57BL/6 J (strain: 000664), B6.FVB-Tg(MMTV-PyVT)634Mul/LellJ (strain: 022974), Tg(MMTV-cre)4Mam/J (strain: 003553) mice from the Jackson Laboratory and C57BL/6 PCBP1 floxed mice (gifted).

C57BL/6 MMTV-PyMT+ MMTV-Cre+ Pcbp1(fl/fl) (abbreviated PyMT *Pcbp1*–/–) or MMTV-PyMT+ MMTV-Cre+ *Pcbp1*(wt/wt) (abbreviated PyMT Pcbp1 + /+) males were then crossed with pure C57BL/6 *Pcbp1*(fl/fl) or (wt/wt) females to generate C57BL/6 MMTV-PyMT+ MMTV-Cre+ *Pcbp1*(fl/fl) or (wt/wt) litters. Exclusively, females developed spontaneous mammary tumors. MMTV-PyMT+ MMTV-Cre+ Pcbp1(fl/fl) and *Pcbp1*(wt/wt) mice were sacrificed at the age of 18 weeks or by size-matching the tumors from the control and knockout groups, depending on experimental needs.

In accordance with the Animal Care and Use Committees of the Medical University of South Carolina, the maximal tumor burden permitted per mouse was defined as a total tumor diameter of 20 mm or a caliper-estimated tumor volume of 4000 mm³. This caliper-based limit was not exceeded in any of the experiments described. Any animal showing signs of stress, discomfort, or pain was euthanized immediately.

### Cell culture

Py8119 (cat. no. CRL-3278, ATCC), and EMT6 (gifted) cells were cultured in Dulbecco’s modified Eagle’s medium high glucose (cat. no. SH30081.01, GE Healthcare Life Sciences) supplemented with 10% fetal bovine serum (cat. no. SH30071.03, Cytiva). HMLE cells (gifted) were cultured in DMEM-F12 supplemented (cat. no. 11320-033, Gibco) with 5% fetal bovine serum, 0.5 μg/ml hydrocortisone (cat. no. DLW354203, Corning), 10 µg/ml insulin (cat. no. 25-800-CR, Corning), and 20 ng/ml epidermal growth factor (cat. no. 354052, Corning). All cell lines were supplemented with 1% antibiotic/antimycotic solution (penicillin G, streptomycin, amphotericin B - cat. no 15240-062, Gibco) and kept in an incubator at 37 °C, 5% CO_2_. Cell cultures were frequently tested to confirm the absence of mycoplasma contamination using the MycoStrip detection kit (cat. no. rep-mysnc-50, Invivogen) according to the manufacturer’s instructions.

### Cell treatments

Fluorescent single and dsDNA oligos were transfected into the cells using the Lipofectamine 3000 reagents (cat. no. 3000015, Invitrogen) according to the manufacturer’s instructions. The transfection solution was incubated with the cells for 20 hours at 37°C, 5% CO_2_, before harvesting. Transfection efficiency of the oligos was controlled by assessing fluorescence intensity using a ZOE cell imager (Bio-Rad).

For IFN-β treatments, 85 ng/mL of recombinant mouse IFN-β (cat. no. 50708-MCCH, Sino Biological) was added to the cell culture media for 12 hours after seeding at ~80% confluence.

For decitabine treatments, cells were cultured with 350 nM decitabine (cat. no. 11166, Cayman Chemical) for 24 hours after seeding at 70–80% confluence.

For cGAS inhibitor treatments, cells at ~80% confluence in 10 cm dishes were pre-cultured with 1 µM of TDI-6570 (cat. no. inh-tdi6570-1, Invivogen) for 3 hours before addition of the transfection reagent containing DNA (see related figures for precise DNA amounts). Cells were transfected in the presence of the inhibitor for 20 hours.

### Lentiviral transduction of small hairpin RNA and overexpression constructs

Stable knockdown cells were generated by lentiviral transduction of pLKO.1 puro vectors containing *PCBP1* shRNA or a scrambled control sequence. Stable cells overexpressing V5-tagged *PCBP1* were generated by lentiviral transduction of plx304 blast vectors. LentiX 293 T cells (cat. no. 632180, Takara Bio) were grown to 70% confluence and transfected with the indicated ShRNA or overexpression plasmids as well as the envelope (pMD2.G) and packaging (psPAX2) plasmids using Lipofectamine 3000 (cat. no. 3000015, Invitrogen) in OPTI-MEM (cat. no. 31985-062, Gibco) according to the manufacturer’s instructions. The medium was changed after overnight incubation. Viruses were then collected after 24 and 48 h by passing through a 0.45-μm sterile filter. For transduction, virus-containing media diluted 1:5 in fresh media were incubated on the target cells with 8 μg/ml polybrene (cat. no. TR-1003, Sigma-Aldrich) overnight. After 48 h, knockdown cells were stably selected in media containing 10 µg/mL puromycin (cat. no. ant-pr-1, Invivogen). V5-tagged *PCBP1* overexpressing cells were stably selected in media containing 10 µg/mL blasticidin (cat. no. Ant-bl-1, Invivogen).

### CRISPR/Cas9 knock-out of PCBP1

*Pcbp1* KO in Py8119 cells was performed using the AltR CRISPR/Cas9 system (IDT). Nucleofection of 2 guides (GATGCCGGTGTGACTGAAAG and CTCCATGACCAACAGTACCG) and AltR Cas9 enzyme into cells was performed using an Amaxa nucleofector and protocol X-013. Edited clones were identified by PCR (primers detailed in Supplementary Table 1) and further characterized by western blot and Sanger sequencing using ICE Synthego software.

### Site-directed mutagenesis

The GXXG motifs in the three KH domains of PCBP1 were mutated to GDDG in the plx304-*PCBP1* overexpression plasmid using a Q5 site-directed mutagenesis kit (cat. no. E0554S, New England Biolabs). Each motif was mutated sequentially through PCR amplification, followed by plasmid propagation, mutation verification by sequencing, and subsequent rounds of mutagenesis for the remaining KH domains. Primer sequences used for each mutation step are listed in Supplementary Table 1.

### Bulk RNA-sequencing

PyMT *Pcbp1*+/+ and PyMT *Pcbp1*–/– mammary tumors were collected from 18-week-old animals and flash frozen in liquid nitrogen. RNA was extracted by Trizol (cat. no. 15596018, Ambion by Life Technologies)/phenol chloroform and analyzed for quality (RIN) by a Bioanalyzer (Agilent 2100). After library preparation by the HCC Translational Science Laboratory (MUSC), paired-end sequencing runs were performed on an Illumina NovaSeq instrument. Read quality was assessed with FastQC, and reads were aligned to GRCm38 with Bowtie2. Differential expression analysis was performed in Partek Flow using the Gene Specific Analysis algorithm. GSEA^71^ was used to identify gene signatures that are upregulated and downregulated in PyMT *Pcbp1*–/– versus PyMT *Pcbp1*+/+ tumors. Interferon-Stimulated Genes were identified using the Interferome database^43^.

### Single-nuclei RNA-sequencing

Sized-matched mammary tumors from PyMT *Pcbp1*+/+ and PyMT *Pcbp1*–/– females were collected and flash frozen in liquid nitrogen. Tissues were chopped, and cell membranes were lysed in NP40 lysis buffer (10 mM Tris-HCl pH 7.4, 10 mM NaCl, 3 mM MgCl2, 1 mM DTT, 0.1% NP40, 1U/µL RNAse inhibitor) using a mini bead mill and crude nuclei suspensions were filtered through 70 µm strainers. Nuclei were spun, resuspended in pre-chilled 1% BSA PBS (cat. no. A7030, Sigma-Aldrich), stained with 10 µg/mL 7AAD (cat. no. A1310, Invitrogen), and positive nuclei were collected by FACS in 1% BSA PBS. Nuclei were processed by the HCC Translational Science Laboratory (MUSC); fluorescently labeled nuclei counts and lack of debris were evaluated with an automated counter (Cellometer, Nexelom Bioscience), and the concentration was adjusted to 1000 nuclei per microliter input for single-nuclei RNA-seq using the Chromium Single Cell 3’ Library v3.1 kit (10X Genomics). Single-nuclei libraries were constructed according to the CG000315 User Guide (10X Genomics), with three QC steps and shipped for sequencing at Vantage (VUMC) on a NovaSeq 6000 (S4 Flow Cell) to a depth of ~300 million paired-end 150 bp reads per library. Data analyses were performed at the Bioinformatics Core (MUSC).

Following experimental procedures respected the established techniques using the Chromium Single Cell 3’ Library v3.1 Kit (10× Genomics). Single-nuclei suspensions were individually loaded onto a 10X Genomics Next GEM Chip G and emulsified with 3’ Single Cell Next GEM beads using a Chromium™ Controller (10× Genomics). From barcoded cDNAs, gene expression libraries were constructed using Chromium™ Next GEM Single Cell 3ʹ Library kit at the Translational Science Laboratory (Medical University of South Carolina).

Next-generation sequencing was performed on each sample using an Illumina NovaSeq S4 flow cell at the VANTAGE facility (Vanderbilt University Medical Center)

The analysis was performed by the bioinformatics core of the Medical University of South Carolina. Raw sequencing data were processed with Cell Ranger (v7.0.0)^72^. Cellranger mkfastq command was used to demultiplex the different samples, and the cellranger count command was used to generate gene–cell expression matrices. The sequences were aligned to the GRCm39 reference genome. Cellranger mkref was used to create a new mm39 reference transcriptome that includes the transgene PyMT. Ambient RNA contamination was inferred and removed using CellBender (v0.232) with standard parameters.

Downstream analysis was performed in R with Seurat (v4.1.0)^73^ and customized R scripts. Data from four different samples were merged into a single dataset. For quality control, cells with >200 genes, <10,000 UMIs, and <5% mitochondrial transcripts were retained for downstream analysis. Genes located in the mitochondrial genome were removed. Doublets were removed using scDblFinder (v1.8.0)^74^ for a resultant expression matrix with 16,568 genes and 37,801 cells.

Prior to integration, the data were normalized and scaled using Seurat’s implementation of SCTransform^75^, regressing out variables of number of UMIs, percent of mitochondrial expression, and cell cycling scores (determined using Seurat’s CellCycleScoring function). The data was integrated via Seurat, using canonical correlation analysis (CCA) to find anchors for integration of the samples.

After integration, PCA was used to cluster all cells, using 30 principal components. Batch correction was also performed using Harmony (v0.1.1)^76^. Cluster markers were identified via the package Presto (https://github.com/immunogenomics/presto) using a Wilcoxon Rank Sum test.

Cell types were annotated using the R package scPred (v.1.9.2)^77^. The Tabula Muris mammary gland dataset was used as a reference ^78^.

To identify differentially expressed genes between conditions, the R package LIBRA (v1.0.0) with option MAST was used to perform zero-inflated regression analysis^79^. Genes were defined as significantly differentially expressed at Benjamini–Hochberg correction FDR < 0.05 and abs(log2(Fold Change)) >0.3.

### Fluorescent DNA and RNA electromobility shift assays

DNA and RNA oligonucleotides fluorescently labeled with Cyanine 5 or 5’6’FAM were ordered from Integrated DNA Technologies. For dsDNA, a 1:1 ratio of Cy5 forward and 5’6’FAM reverse oligos was annealed in boiling water and cooled down at room temperature overnight. Oligos and human His-tagged cGAS recombinant protein (cat. no. 22810, Cayman chemical) or GST-tagged PCBP1 (made in-house) were mixed in a 50 μl reaction buffer that contained 25 mM Tris-HCl (pH 7.6), 1 mM EDTA, 2% glycerol, 10 mM βME and 50 μg/ml BSA. The reaction mix was incubated at room temperature for 15 min before adding a final concentration of 5% glycerol. Samples were resolved on 6% or 20% mini native PAGE in 1× Tris-Borate-EDTA (TBE) buffer at 100 V for ~45 min. Fluorescence was detected by a CCD camera (Bio-Rad ChemiDoc system). EMSA bands were analyzed and quantified using ImageJ.

### Biotinylated nucleic acids pulldowns

Cells were lysed in lysis buffer (0.5% NP40, 1% Triton X-100, 150 mM NaCl, 50 mM Tris-HCl, pH 7.4, 1 mM EDTA, 1 mM EGTA). Protease phosphatase inhibitor cocktail (cat. no. 78425, Thermo Fisher Scientific) was added before protein quantification using the Pierce Micro BCA Protein Assay kit (cat. no. 23235, Thermo Fisher Scientific). 500 µg of lysates were incubated with 4 µg of biotinylated oligos for 2 hours at 4 degrees Celsius. Agarose streptavidin beads (cat. no. 15942-050, Invitrogen) previously blocked in 5% BSA (cat. no. A7030, Sigma-Aldrich) in lysis buffer for 1 hour at room temperature were added to the lysate/oligo mixture and incubated for 1 hour at room temperature. Three washes were performed in lysis buffer before the addition of Laemmli buffer and denaturation. Samples were analyzed by immunoblotting with anti-PCBP1 (RN024P, MBL Life Science) antibody, HSP90 (sc-13119 HRP, Santa Cruz Biotechnology) antibody and mouse cGAS (31659S, Cell Signaling Technology) and Chemiluminescence was detected by CCD camera (Bio-Rad ChemiDoc system).

### Co-immunoprecipitation of recombinant proteins

3 µg of recombinant GST-PCBP1 (made in-house, 2 µg of His-cGAS (cat. no. 22810, Cayman Chemical) and 2 µg of C4-YSD unlabeled oligo were added to an IP buffer composed of 0.05% NP40, 150 mM NaCl and 50 mM Tris-HCl 7.4 and incubated at 4 °C for 1 h on a rotator. 3 µg of anti-His tag antibody (cat. no. 2365S, Cell Signaling Technology) was added to the mixture and incubated at 4 °C for 2 h on a rotator. 50 µL of protein A agarose beads, previously washed with IP buffer, were added per condition and incubated at 4 °C for 2  h on a rotator. Samples were washed three times with IP buffer before denaturation of the protein/bead complexes with Laemmli buffer and incubation at 95 degrees Celsius for 5 min. Samples were analyzed by immunoblotting with anti-His (cat. no. 2365S, Cell Signaling Technology) and anti-PCBP1 (cat. no. RN024P, MBL Life Science) antibodies, before washes and incubation with an HRP-conjugated conformation-specific anti-rabbit antibody (cat. no. 5127S, Cell Signaling Technology). Chemiluminescence was detected by a CCD camera (Bio-Rad ChemiDoc system).

### 2′,3′-cyclic GAMP enzyme-linked immunosorbent assay

Adherent cells were washed with PBS and lysed using the M-PER Mammalian Protein Extraction Reagent (cat. no. 78503, Thermo Fisher Scientific). Protein concentrations were determined for loading normalization using the Pierce Micro BCA Protein Assay kit (cat. no. 23235, Thermo Fisher Scientific) before performing the 2′,3′-Cyclic GAMP ELISA kit (cat. no. 501700, Cayman Chemical) according to the manufacturer’s instructions.

### Dual immunofluorescence

For the CD8 and CD3 immunohistofluorescence staining, mouse mammary tumors were formalin-fixed, paraffin-embedded sections that were then deparaffinized in xylene and rehydrated in decreasing alcohol concentration baths. Heat-induced antigen Retrieval was performed in EDTA pH8.5 retrieval buffer (1 mM EDTA, 0.05% Tween20, pH8.5) at 95 °C for 15 minutes before getting blocked with 1% bovine calf serum in PBS for 30 min at room temperature. Anti-CD8 (cat. no. D4W2Z, CST, 1:200 dilution) and anti-CD3 (cat. no. AB11089, Abcam, 1:100 dilution) antibodies were diluted in blocking buffer and incubated overnight at 4 °C in a humid chamber. After three washes of PBS, AF488 (cat. no. A11034, Invitrogen, 1:250 dilution) and AF594 (cat. no. A11037, Invitrogen, 1:250 dilution) secondary antibodies were added for 1 h at room temperature, in the dark, followed by three PBS washes and mounting of slides. Multiplex IHC was analyzed using an Akoya Vectra Polaris automated imaging system (Akoya Biosciences). Primary analyses were conducted on blinded datasets; group labels were disclosed only after the analysis plan was finalized.

### Tumor-infiltrating lymphocytes analysis by flow cytometry

Sized-matched mammary tumors were excised and chopped with a razor blade. Tumors were then digested using the gentleMACS tissue dissociator (Miltenyi biotec, cat. no. 130-096-427) and the mouse tumor dissociation kit (Miltenyi biotec, cat. no. 130-096-730) according to the manufacturer’s instructions, followed by filtration through 40µm cell strainers to obtain single cell suspensions. Staining for cell surface markers was performed by incubating cells with the antibody at 1:200 dilutions (anti-CD8a-PE-Cy7, cat. no. 100722, Biolegend, and anti-CD3-A700, cat. no. 5613388, BD Pharmingen) and with LIVE/DEAD Fixable Yellow Dead Cell Stain Kit (Invitrogen, cat. no. L34959) in PBS 2% FBS for 30 min at 4 °C. Cells were then fixed with 4% paraformaldehyde for 20 min at 4°C before resuspending in PBS. Tumor samples, spleens and fluorescence minus one as control samples were acquired on LSRFortessa and analyzed with FlowJo software (Tree Star, OR). Primary analyses were conducted on blinded datasets; group labels were disclosed only after the analysis plan was finalized.

### Reverse transcription and quantitative PCR

Total RNA from cell lines was isolated using the quick RNA mini-prep kit (cat. no. R1055, Zymo Research). Total RNA was extracted from age-matched PyMT P*cbp1*+/+ and PyMT P*cbp1*–/– tumors by placing the tumor samples in tubes containing 2.8 mm ceramic beads (cat. no. 10158-612, VWR) and homogenizing them in TRIzol reagent using a mini bead mill homogenizer (VWR) for four cycles of 30 seconds at maximum speed. Chloroform was then added before carrying out the rest of the extraction according to the manufacturer’s instructions. cDNA synthesis was performed using the qScript cDNA synthesis kit (cat. no. 95047, Quantabio) with 1 µg of total RNA. Real-time quantitative PCR was conducted using iQ SYBR Green Supermix (cat. no. 1708880, Bio-Rad) on the CFX384 Real-Time System (Bio-Rad). Relative gene expression was calculated through RFX Manager software, normalizing expression to 18S or PSMD4 internal control.

### Western blotting

Cells cultured in vitro were lysed in RIPA buffer with protease phosphatase inhibitor cocktail (cat. no. 78425, Thermo Fisher Scientific). Age-matched PyMT *Pcbp1*+/+ and PyMT *Pcbp1*–/– tumors were placed in tubes containing 2.8 mm ceramic beads (cat. no. 10158-612, VWR) and homogenized in the same RIPA buffer with inhibitors using a mini bead mill homogenizer (VWR). Homogenization was performed for four cycles of 30 seconds at maximum speed, with samples kept on ice between cycles. Protein concentrations were determined using the Pierce Micro BCA Protein Assay kit (cat. no. 23235, Thermo Fisher Scientific) prior to Laemmli sample buffer addition and heat denaturation at 95°C for 5 min. Samples were resolved on SDS–PAGE, transferred to PVDF membrane and blocked in 5% milk TBST. Transferred proteins were immunoblotted. Chemiluminescence was detected by CCD camera (Bio-Rad ChemiDoc system).

List of antibodies used for western blotting:

**Table:** 

Antibody	Manufacturer	Catalog number
Anti-human cGAS	Cell Signaling Technology	cat. no. 15102S
Anti-mouse cGAS	Cell Signaling Technology	cat. no. 31659S
Anti-His tag	Cell Signaling Technology	cat. no. 2365S
Anti-mouse/human HSP90 HRP-conjugate	Santa Cruz Biotechnology	cat. no. sc-13119 HRP
Anti-mouse/human IRF3	Santa Cruz Biotechnology	cat. no. 4302S
Anti-mouse IRF7	Cell Signaling Technology	cat. no. 72073S
Anti-mouse/human ISG15	Santa Cruz Biotechnology	cat. no. sc-166755
Anti-mouse/human PCBP1	MBL Life Science	cat. no. RN024P
Anti-mouse phospho-IRF3	Cell Signaling Technology	cat. no. 29047S
Anti-mouse phospho-STING	Cell Signaling Technology	cat. no. 72971S
Anti-human phospho-STING	Cell Signaling Technology	cat. no. D7C3S
Anti-mouse phospho-TBK1	Cell Signaling Technology	cat. no. 5483S
Anti-mouse STING	Cell Signaling Technology	cat. no. 50494S
Anti-mouse TBK1	Cell Signaling Technology	cat. no. 3504S
Anti-V5-tag	Invitrogen	cat. no. R96025

### Enzyme kinetics

A master reaction mix was prepared before adding the PCBP1 protein to the + PCBP1 condition or water to the—PCBP1 reaction. The final reaction was composed of 100 µM ATP, 100 µM GTP, 30 nM human His-tagged recombinant cGAS (cat. no. 22810, Cayman Chemical), 10 mM Tris-HCl pH 7.5, 2.5 mM MgCl_2_ and 0.005% Tween20 and with or without 60 nM of His-tagged recombinant PCBP1 (cat. no. 11628-H07E, Sino Biological). 15 nM or 500 nM substrate (unlabeled HSV120 dsDNA or C4-YSD or G4-YSD, purchased from Eurofins) was then added for non-saturating and saturating conditions, respectively, or with increasing C4-YSD concentrations for the Michaelis-Menten kinetics to start the reaction. The enzymatic reaction was carried out for 30 seconds or 5 minutes before being stopped by the addition of 5 mM EDTA. 2’3’-cGAMP levels were measured by ELISA according to the manufacturer’s instructions without any prior dilution of the samples.

### Statistics and reproducibility

All data are presented as means ± SEM unless stated otherwise. Representative experiments were independently repeated at least three times, including all EMSAs, immunoblots, and pulldown assays presented in this study. Analysis of continuous measures was performed using *t* tests or ANOVA for two- or multi-group comparisons, respectively. Statistical analysis was performed using GraphPad Prism 10. *P* < 0.05 was considered statistically significant (*P < 0.05, **P < 0.01, ***P < 0.001, ****P < 0.0001, ns: not statistically significant).

## Supplementary information

Transparent Peer Review file

Supplementary Information

Description of Additional Supplementary File

Supplementary data

## Data Availability

Bulk and single nuclei RNA-sequencing datasets generated during this study are deposited in the Gene Expression Omnibus (GEO) under accession numbers GSE307616 and GSE307750, respectively. Source data are provided with this paper in the supplementary data. The TCGA BRCA dataset is available online at https://www.cancer.gov/tcga. The Tabula Muris dataset is available online at http://tabula-muris.sf.czbiohub.org. Unedited Western blot images are available in the Supplementary Information.
