# Supplementary material for: PCBP1 binding to single-stranded poly-cytosine motifs enhances cGAS sensing and impairs breast cancer development
Source: Commun Biol. 2026 Jan 7;9:179. doi: 10.1038/s42003-025-09456-z (PMC12881503; doi:10.1038/s42003-025-09456-z)

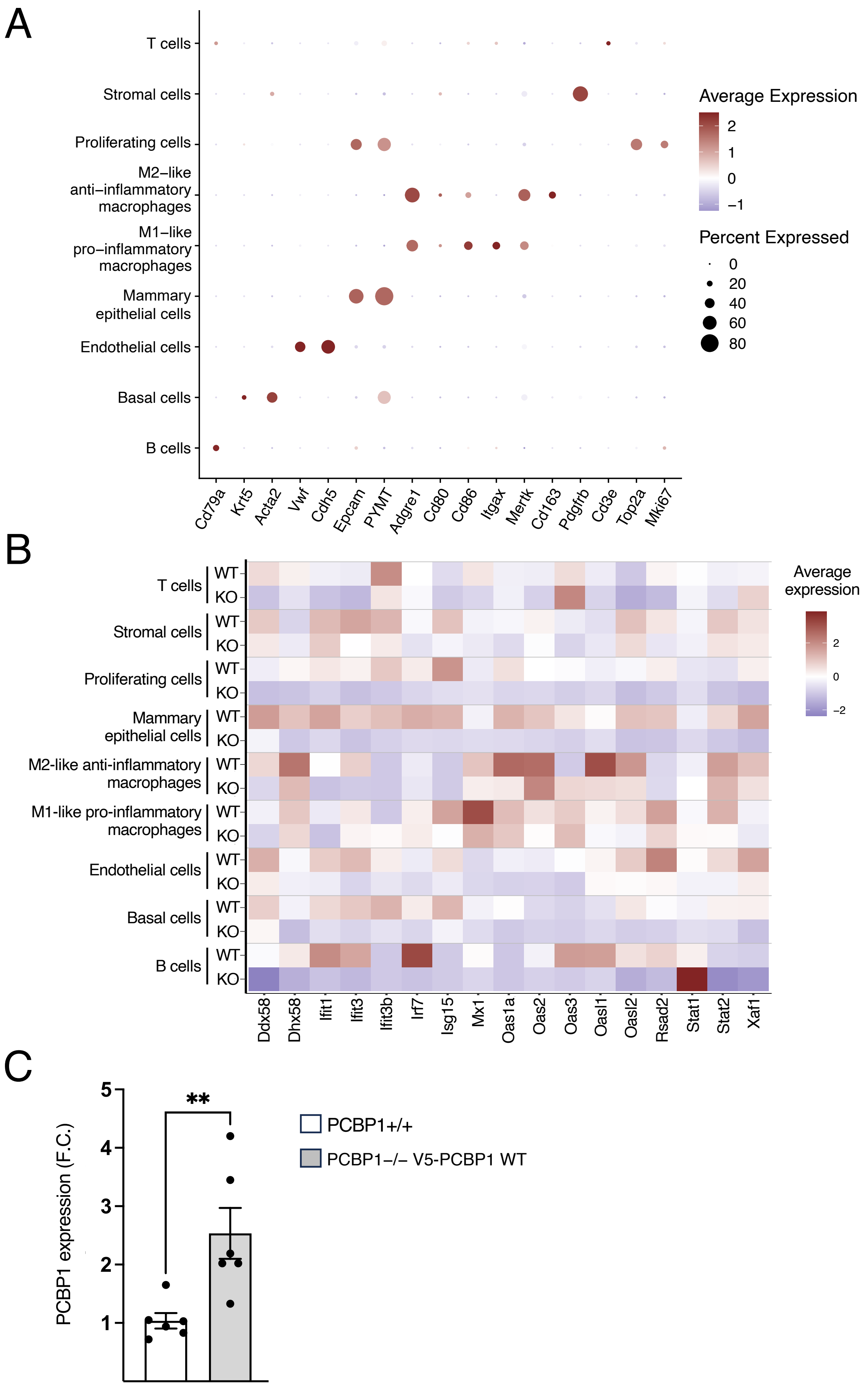
A
T cells
Stromal cells
Proliferating cells
M2–like anti–inflammatory macrophages
M1–like pro–inflammatory macrophages
Mammary epithelial cells
Endothelial cells
Basal cells
B cells
Cd79a
Krt5
Acta2
Vwf
Cdh5
Epcam
PYMT
Adgre1
Cd80
Cd86
Itgax
Mertk
Cd163
Pdgfrb
Cd3e
Top2a
Mki67
Average Expression
2
1
0
−1
Percent Expressed
0
20
40
60
80
B
T cells
Stromal cells
Proliferating cells
Mammary epithelial cells
M2-like anti-inflammatory macrophages
M1-like pro-inflammatory macrophages
Endothelial cells
Basal cells
B cells
WT
KO
Ddx58
Dhx58
Ifit1
Ifit3
Ifit3b
Irf7
Isg15
Mx1
Oas1a
Oas2
Oas3
Oasl1
Oasl2
Rsad2
Stat1
Stat2
Xaf1
Average expression
2
0
−2
C
PCBP1 expression (F.C.)
5
4
3
2
1
**
PCBP1+/+
PCBP1−/− V5-PCBP1 WT

Supplementary Figure 1: Characterization of mammary tumor cell populations and validation of *Pcbp1* rescue expression *in vitro*. **A**. Plot showing the expression of commonly used markers to identify the different cell types. **B**. Heatmap representing ISG expression in the annotated cell types of pooled PyMT *Pcbp1*+/+ (n=2) and PyMT Pcbp1–/– (n=2) mammary tumor samples. **C.** RT-qPCR analysis of *Pcbp1* mRNA levels in Py8119 *Pcbp1*–/– cells rescued with V5-*PCBP1* WT (n=6) relative to Py8119 *Pcbp1*+/+ cells (n=6), (Mean ± SEM, two-tailed unpaired t.test).

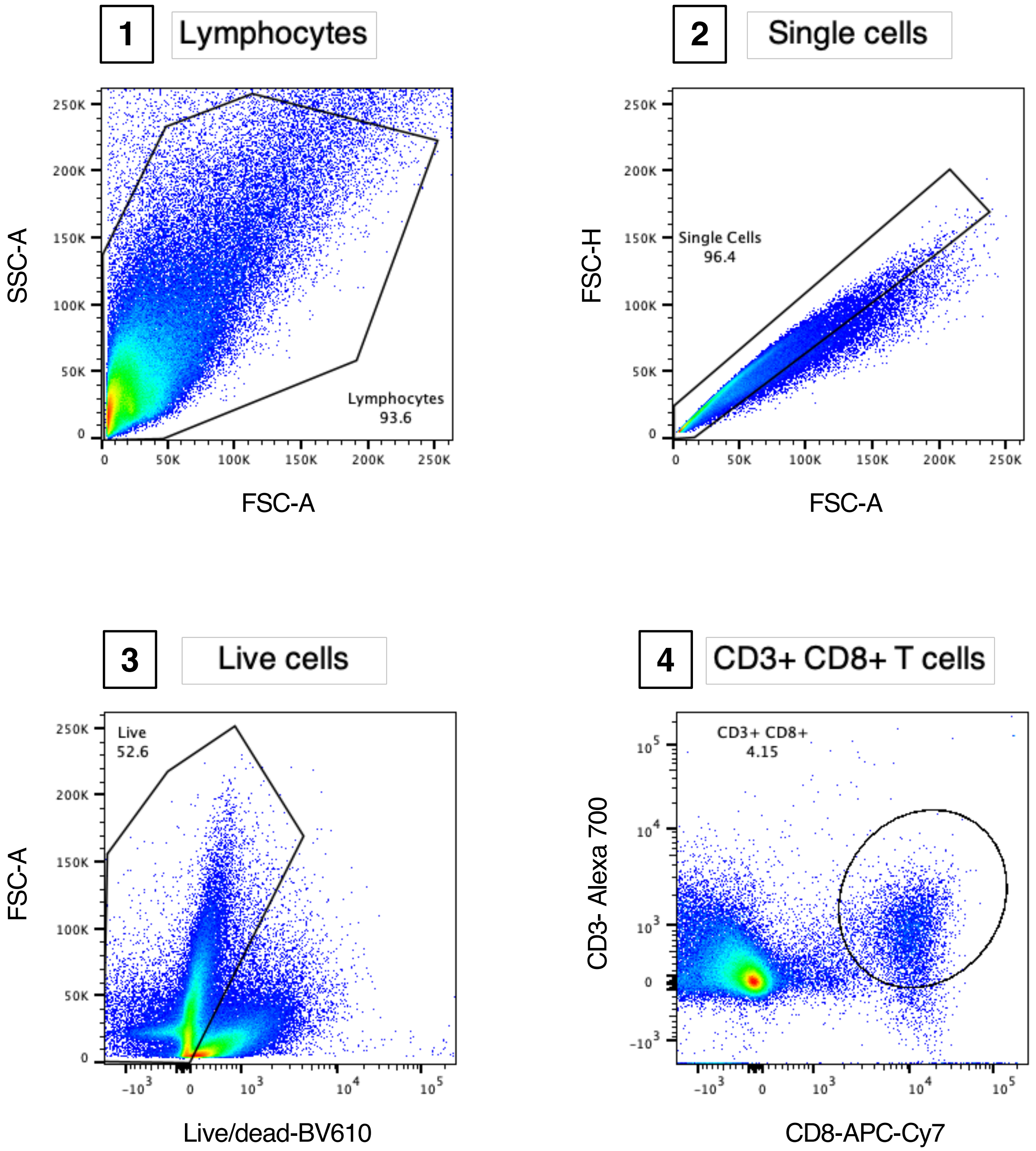

Supplementary Figure 2: Gating strategy for analysis of intra-tumoral $CD3^{+}CD8^{+}$ cytotoxic T cell infiltration by flow cytometry. Gating was confirmed using fluorescence minus one (FMO) controls and validated with splenic tissues, which contains a high frequency of $CD3^{+}CD8^{+}$ T cells.

Py8119 PCBP1+/+ vehicle (lipofectamine 3000)

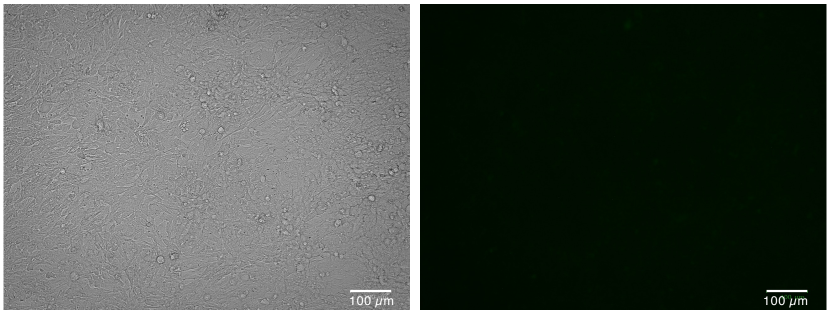

Py8119 PCBP1+/+ HSV120 ssDNA

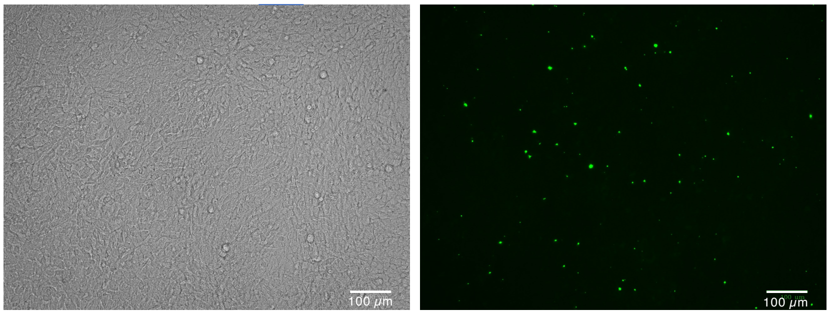

Py8119 PCBP1–/– HSV120 ssDNA

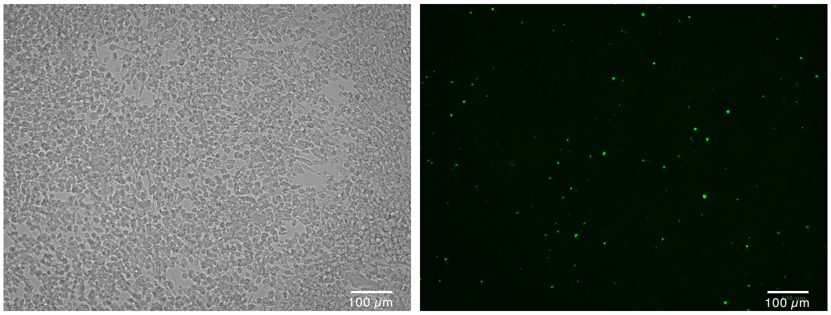

Py8119 PCBP1–/– V5-PCBP1 WT HSV120 ssDNA

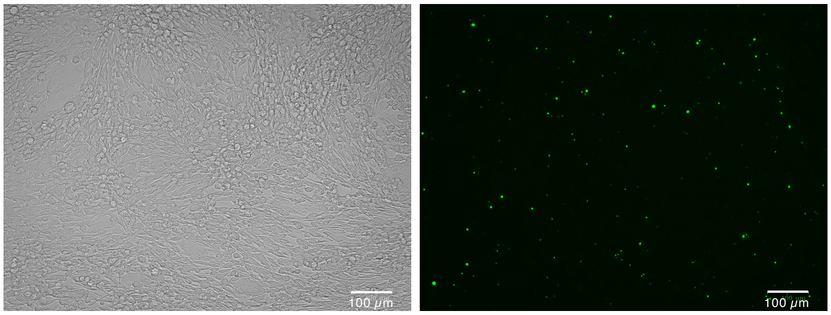

Py8119 PCBP1–/– V5-PCBP1 GDDG HSV120 ssDNA

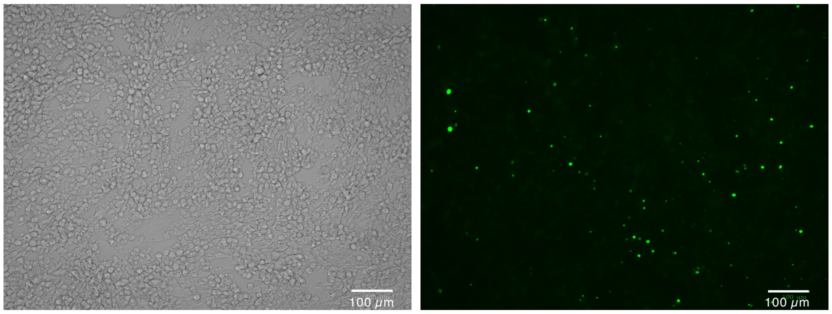

Supplementary Figure 3: Fluorescence microscopy images of Py8119 *Pcbp1*+/+, *Pcbp1*–/–, *Pcbp1*–/– V5-*PCBP1* WT and *Pcbp1*–/– V5-*PCBP1* GDDG mutant cells transfected with vehicle (lipofectamine 3000 only) or fluorescently labeled HSV120 single-stranded (ssDNA) DNA. Left panels: brightfield; right panels: green fluorescence (scale bars: 100 µm).

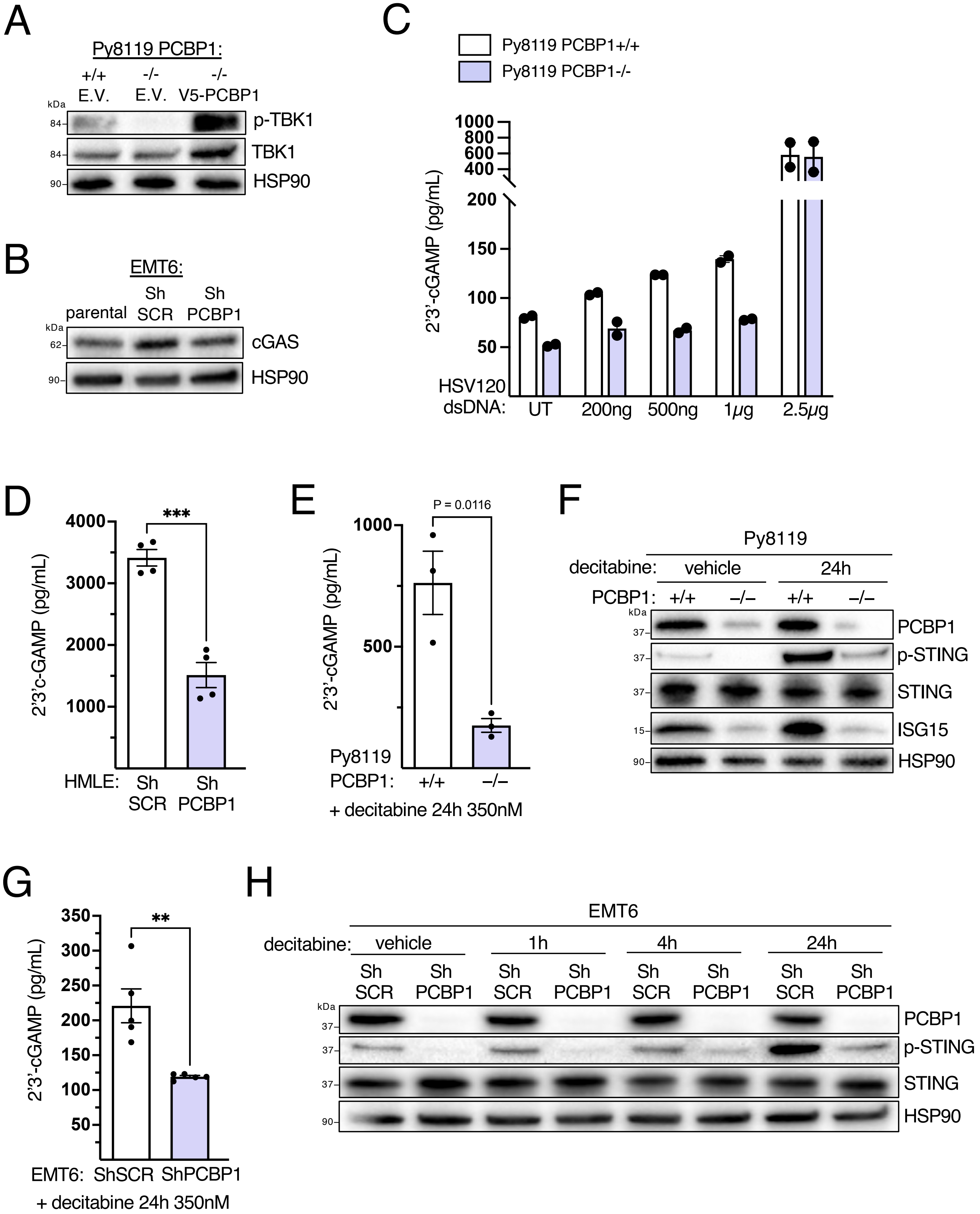
A
Py8119 PCBP1:
+/+ E.V.
-/- E.V.
-/- V5-PCBP1
kDa
84
84
90
p-TBK1
TBK1
HSP90
B
EMT6:
parental
Sh SCR
Sh PCBP1
kDa
62
90
cGAS
HSP90
C
Py8119 PCBP1+/+
Py8119 PCBP1-/-
2'3'-cGAMP (pg/mL)
1000
800
600
400
200
150
100
50
HSV120 dsDNA:
UT
200ng
500ng
1μg
2.5μg
D
2'3'c-GAMP (pg/mL)
4000
3000
2000
1000
***
HMLE:
Sh SCR
Sh PCBP1
E
P = 0.0116
2'3'-cGAMP (pg/mL)
1000
500
Py8119 PCBP1:
+/+
–/–
+ decitabine 24h 350nM
F
Py8119
decitabine:
vehicle
24h
PCBP1:
+/+
–/–
+/+
–/–
kDa
37
37
37
15
90
PCBP1
p-STING
STING
ISG15
HSP90
G
2'3'-cGAMP (pg/mL)
350
300
250
200
150
100
50
**
EMT6:
ShSCR
ShPCBP1
+ decitabine 24h 350nM
H
EMT6
decitabine:
vehicle
1h
4h
24h
Sh SCR
Sh PCBP1
Sh SCR
Sh PCBP1
Sh SCR
Sh PCBP1
Sh SCR
Sh PCBP1
kDa
37
37
37
90
PCBP1
p-STING
STING
HSP90

Supplementary Figure 4: PCBP1 promotes cGAS-STING pathway activation in response to decitabine treatment and under low amount of cytoplasmic polyC dsDNA. **A.** Immunoblot of cGAS-STING-type I IFN activation marker: p-TBK1 as well as total TBK1 and HSP90 in Py8119 *Pcbp1*+/+ cells vs *Pcbp1*–/– or *Pcbp1*–/– rescued with V5-tagged WT *PCBP1*. **B**. Immunoblot of cGAS and HSP90 in parental, shScramble and sh*PCBP1* EMT6 mouse breast cancer cell line. **C.** Quantification of intracellular 2′3′-cGAMP quantification in Py8119 *Pcbp1*+/+ (n=2) and *Pcbp1*–/– cells (n=2) untreated or transfected with increasing amounts of polyC HSV120 blunt-ended dsDNA (200ng, 500ng, 1µg or 2.5µg per 10cm dish) for 20h. Data are represented as mean ± SEM, two-tailed unpaired t.test). **D.** 2′3′-cGAMP quantification in untreated HMLE and **E.** in Py8119 cells with stable *Pcbp1* knockdown (Sh*PCBP1*) or scramble control (ShSCR), treated with vehicle (DMSO) or 350nM decitabine for 24h. **F.** Immunoblot analysis of the indicated markers in Py8119 *Pcbp1*+/+ or *Pcbp1*–/– cells treated with vehicle (DMSO) or decitabine 350nM decitabine for 24h. **G.** 2′3′-cGAMP quantification in EMT6 ShSCR or Sh*PCBP1* cells treated with vehicle (DMSO) or 350nM decitabine for 24h. **H.** Immunoblot of the indicated markers in EMT6 cells with stable *Pcbp1* knockdown (Sh*PCBP1*) or scramble control (ShSCR) after 24h of vehicle (DMSO) treatment or decitabine treatment for 1, 4, or 24h.

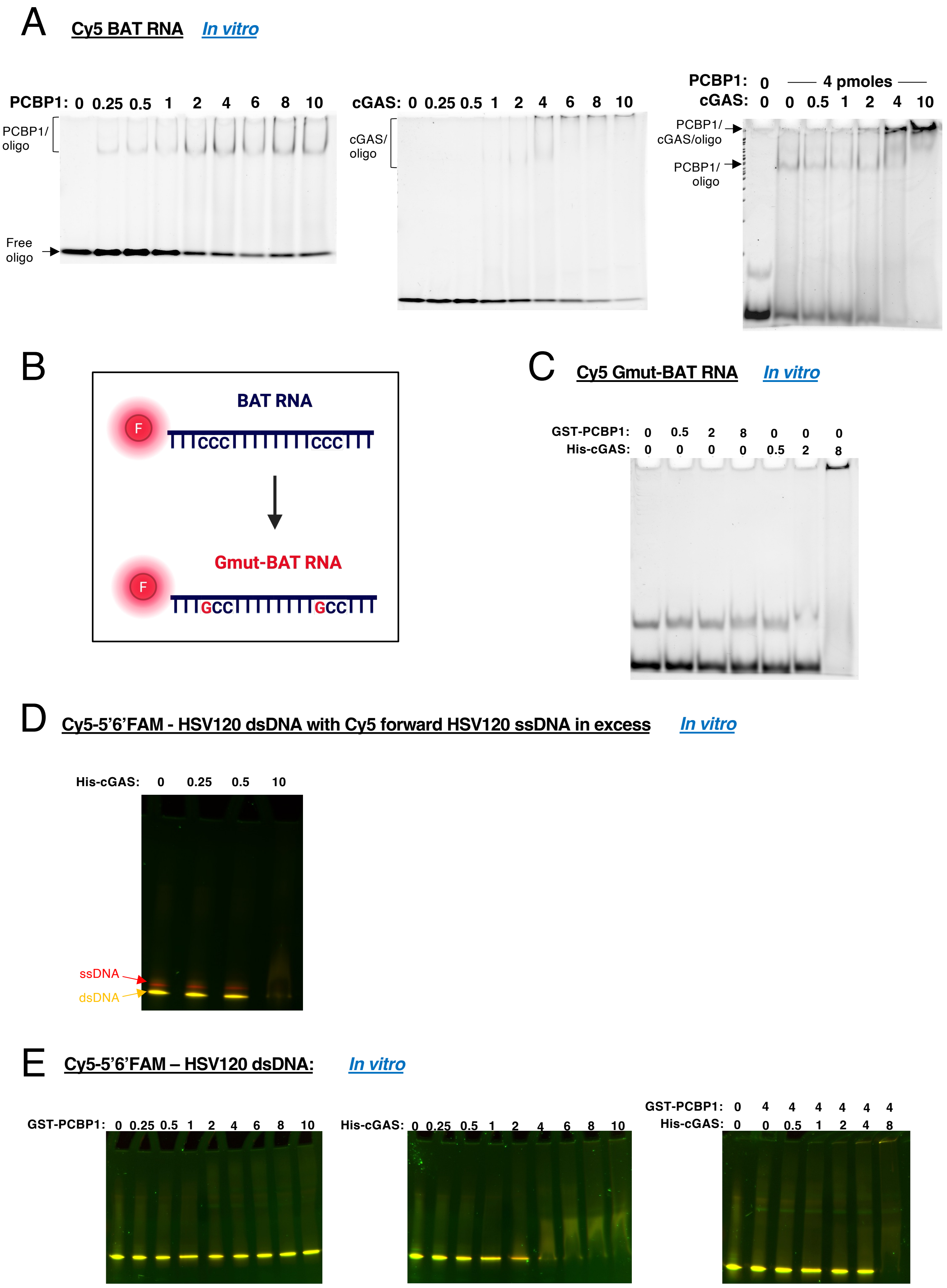
A
Cy5 BAT RNA
In vitro
PCBP1: 0 0.25 0.5 1 2 4 6 8 10
PCBP1/ oligo
Free oligo
cGAS: 0 0.25 0.5 1 2 4 6 8 10
cGAS/ oligo
PCBP1: 0 —— 4 pmoles ——
cGAS: 0 0 0.5 1 2 4 10
PCBP1/ cGAS/oligo
PCBP1/ oligo
B
BAT RNA
F
CCC CCC
Gmut-BAT RNA
F
GCC GCC
C
Cy5 Gmut-BAT RNA
In vitro
GST-PCBP1: 0 0.5 2 8 0 0 0
His-cGAS: 0 0 0 0 0.5 2 8
D
Cy5-5'6'FAM - HSV120 dsDNA with Cy5 forward HSV120 ssDNA in excess
In vitro
His-cGAS: 0 0.25 0.5 10
ssDNA
dsDNA
E
Cy5-5'6'FAM – HSV120 dsDNA:
In vitro
GST-PCBP1: 0 0.25 0.5 1 2 4 6 8 10
His-cGAS: 0 0.25 0.5 1 2 4 6 8 10
GST-PCBP1: 0 4 4 4 4 4 4
His-cGAS: 0 0 0.5 1 2 4 8

Supplementary Figure 5: PCBP1 and cGAS binding to the BAT RNA and confirmation of double-stranded HSV120 annealing**. A.** EMSA showing *in vitro* binding of recombinant human PCBP1 (left), human cGAS (middle), or both (right) to Cy5-labeled BAT RNA (units are in pmoles). **B.** Representation of Cy5-labeled wild-type BAT RNA and Gmut-BAT RNA with mutated polyC motifs. **C.** EMSA using Cy5-labeled Gmut-BAT RNA with human GST-PCBP1 or human His-cGAS. **D.** EMSA on gradient Native PAGE with Cy5/5′6-FAM HSV120 dsDNA and excess Cy5-labeled ssDNA (forward strand). **E.** EMSA on gradient Native PAGE of Cy5/5′6-FAM HSV120 dsDNA incubated with increasing human GST-PCBP1 (left), human His-cGAS (middle), or both proteins (right).

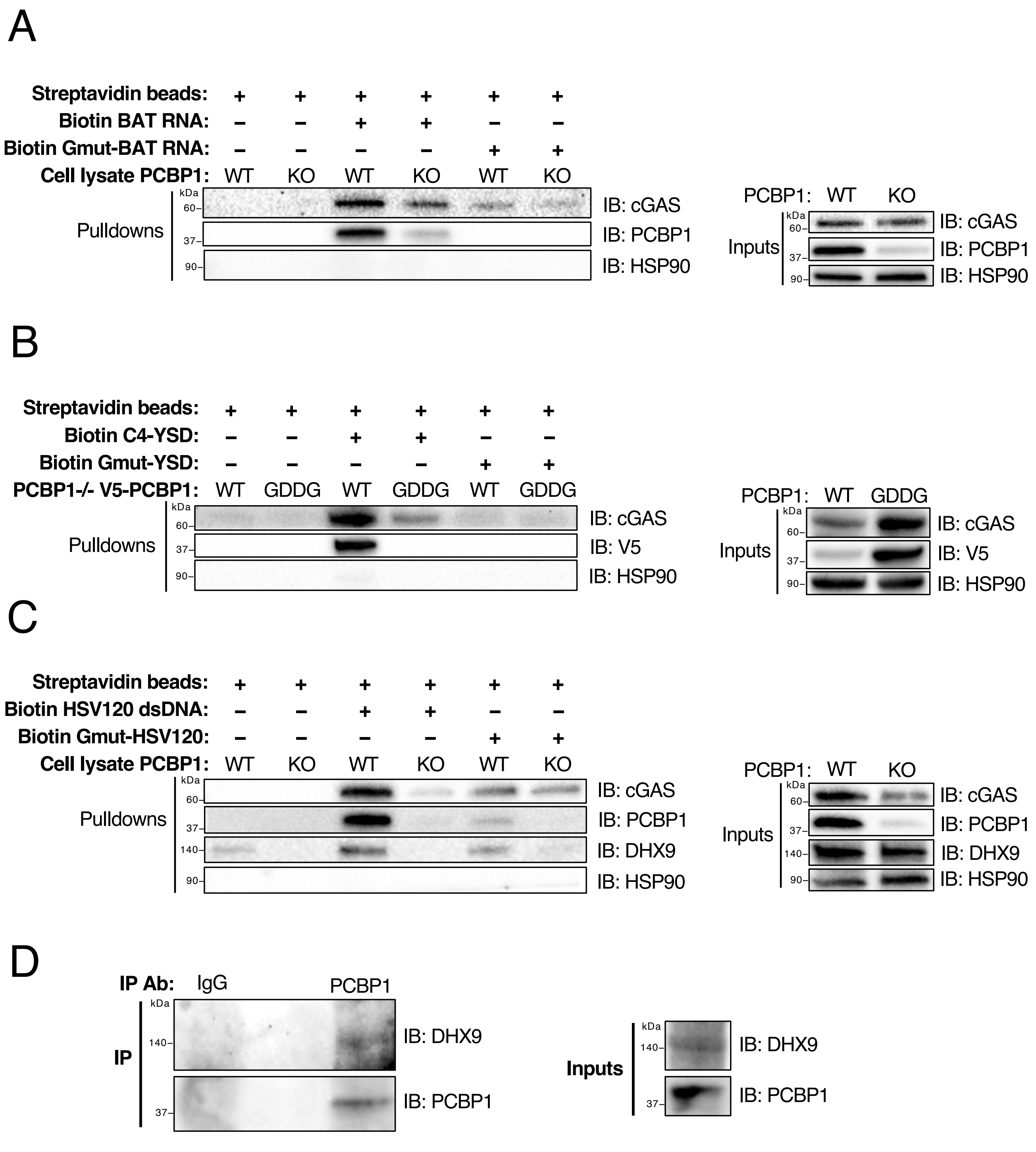

Supplementary Figure 6: PCBP1 and cGAS binding to polyC versus Gmutant oligos. **A.** Streptavidin pulldown from Py8119 WT or *Pcbp1* KO cell lysates incubated with biotin-labeled BAT RNA or Gmut-BAT RNA followed by immunoblotting for cGAS, PCBP1 and HSP90 as a negative control in the pulldown and loading control in the input. **B.** Streptavidin pulldown from Py8119 *Pcbp1* KO rescued with V5-*PCBP1* WT or GDDG mutant cell lysates incubated with biotin-C4-YSD or Gmut-YSD followed by immunoblotting for cGAS, PCBP1 and HSP90. **C.** Streptavidin pulldown from Py8119 *PCBP1* WT or *PCBP1* KO cell lysates incubated with biotin-labeled HSV120 dsDNA or Gmut-HSV120 dsDNA followed by immunoblotting for cGAS, PCBP1, DHX9 and HSP90. **D.** Co-immunoprecipitation of DHX9 with PCBP1 from Py8119 *Pcbp1*+/+ cells using anti-PCBP1 or control IgG antibody and analyzed by immunoblotting for DHX9 and PCBP1.

Supplementary Table 1: List of oligonucleotides and PCR primers used in the study.

Capitalized bold underlined black text indicates poly-cytosine sequences.
Capitalized bold underlined red text indicates guanine residues replacing cytosines in the mutant constructs.
Lowercase underlined red text indicates the mutated residues introduced to convert *PCBP1* GXXG loops into GDDG motifs.

**Oligonucleotides**	
**HSV120 Forward**	TTTTTGCGTTATCACTGT**CCC**GGATTGGACACGGTCTTGTGGGATAGGCATG**CCC**AGAAGGCATATTGGGTTAA**CCCC**TTTTTATTTGTGGCGGGTTTTT
**HSV120 Reverse**	AAAAA**CCC**GCCACAAATAAAAAGGGGTTAA**CCC**AATATGCCTTCTGGGCATGCCTAT**CCC**ACAAGACCGTGTCCAATCCGGGACAGTGATAACGCAAAAA
**Gmut-HSV120 Forward**	TTTTTGCGTTATCACTGT**GCC**GGATTGGACACGGTCTTGTGGGATAGGCATG**GCC**AGAAGGCATATTGGGTTAA**CGCC**TTTTTATTTGTGGCGGGTTTTT
**Gmut-HSV120 Reverse**	AAAAA**GCC**GCCACAAATAAAAAGGGGTTAA**GCC**AATATGCCTTCTGGGCATGCCTAT**GCC**ACAAGACCGTGTCCAATCCGGGACAGTGATAACGCAAAAA
**C4-YSD Forward**	**CCCC**GAACTCCAGCAGGACCATTG**CCCC**
**C4-YSD Reverse**	**CCCC**CAATGGTCCTGCTGGAGTTC**CCCC**
**Gmut-YSD Forward**	**CGCG**GAACTCCAGCAGGACCATTG**GCGC**
**Gmut-YSD Reverse**	**CGCG**CAATGGTCCTGCTGGAGTTC**GCGC**
**BAT RNA**	AAUUGCUA**CCC**AAUGCCUGGAAGGGCAUU**CCC**AAAGCUUA
**Gmut BAT-RNA**	AAUUGCUA**GCC**AAUGCCUGGAAGGGCAUU**GCC**AAAGCUUA
**PCR primers**	
**Mouse 18S Forward**	GTAACCCGTTGAACCCCATTCG
**Mouse 18S Reverse**	CCATCCAATCGGTAGTAGCGAC
**Mouse BST2 Forward**	CACAGGCAAACTCCTGCAAC
**Mouse BST2 Reverse**	TCCTGGTTCAGCTTCGTGAC
**Mouse CXCL10 Forward**	CCACGTGTTGAGATCATTGCC
**Mouse CXCL10 Reverse**	GAGGCTCTCTGCTGTCCATC
**Mouse IFIT1 Forward**	TTACAGCAACCATGGGAGAGAATG
**Mouse IFIT1 Reverse**	TTGGCTGCATAGCGAATGACA
**Mouse IFNB1 Forward**	CCAGCTCCAAGAAAGGACGA
**Mouse IFNB1 Reverse**	CATCCAGGCGTAGCTGTTGT
**Mouse IRF7 Forward**	AGCTTGGATCTACTGTGGGC
**Mouse IRF7 Reverse**	GGGTTCCTCGTAAACACGGT
**Mouse ISG15 Forward**	TCTGACTGTGAGAGCAAGCAG
**Mouse ISG15 Reverse**	ACCTTTAGGTCCCAGGCCATT

**Mouse PCBP1 Forward**	CTGACTGGGCCTACCAATGC
**Mouse PCBP1 Reverse**	GCCGTACTGTTGGTCATGGA
**Mouse PSMD4 Forward**	TCCCAGTGACTTTGAGTTTGGAG
**Mouse PSMD4 Reverse**	GCTGCCGCTGCTCTTCCATA
**Mouse OAS2 Forward**	GGTGGGAGTGTTCACTACAGG
**Mouse OAS2 Reverse**	GGGGGTCTGCATTACCTAGAC
**Mouse PCBP1 CRISPR Forward**	AGTCCGTCACCGAGTGTGTG
**Mouse PCBP1 CRISPR Reverse**	CATGGGCTGGTACGGGATGG
**Human PCBP1 KH1 GDDG Forward**	CATCATCGGGgAcgAtGGGGAGTCGGTG
**Human PCBP1 KH1 GDDG Reverse**	CTGCCTACTTCCTTTCCGTG
**Human PCBP1 KH2 GDDG Forward**	CCTGATCGGCgAcGaCGGCTGCAAGATCAAGGAGATCCG
**Human PCBP1 KH2 GDDG Reverse**	GAGCCACACTGGGTGGCGGG
**Human PCBP1 KH3(1) GDDG Forward**	CATAATCGGGgaCgAtGGCGCCAACATT
**Human PCBP1 KH3(1) GDDG Reverse**	CAGCCGATTAAGTTATTTGG
**Human PCBP1 KH3(2) GDDG Forward**	AGTGGAAGGCgaCgaTGGTAGGCAGGTTAC
**Human PCBP1 KH3(2) GDDG Reverse**	GGGTTGGCAATTTTGATCTG
**Human PCBP1 KH3(2) WT Forward**	GAAGGCTCCTCTGGTAGGCA
**V5 Reverse**	ACCGAGGAGAGGGTTAGGGAT

Supplementary Table 2: Proportions of each cell type between pooled PyMT *Pcbp1*+/+ (n=2) and PyMT *Pcbp1*–/– (n=2) mammary tumor samples.

	**Cell percentage PyMT *Pcbp1*+/+**	**Cell percentage PyMT *Pcbp1*−/−**
**Mammary epithelial cells**	86.9%	78.0%
**Basal cells**	4.2%	10.7%
**Proliferating cells**	3.5%	4.3%
**Stromal cells**	2.4%	3.5%
**Endothelial cells**	0.93%	1.56%
**T cells**	0.82%	0.30%
**B cells**	0.19%	0.00005%
**M1-like pro-inflammatory macrophages**	0.67%	0.95%
**M2-like anti-inflammatory macrophages**	0.34%	0.69%

Supplementary Tables 3: Initial reaction velocities (V_0) measured in presence of different nucleic acids, in non-saturating or saturating conditions, with final concentration of cGAS at 30nM. 2'3'cGAMP formation was quantified at 30 seconds or 5 minutes, and V_0 (nM/min) was calculated based on these time points.

OD = optical density; BR = biological replicate; V_0 = initial velocity; STDEV = standard deviation; SEM = standard error of the mean; FC = fold change.

Non-saturating conditions: C4-YSD 15nM, reaction of 30 seconds:

C4-YSD	OD BR1	OD BR2	OD BR3	V_0 1 (nM/min)	V_0 2 (nM/min)	V_0 3 (nM/min)	V0 average	STDEV	SEM	FC
**- PCBP1**	0.796	0.724	0.704	4.83	5.14	5.23	5.23	0.452	0.261	1.00
**+ PCBP1**	0.683	0.614	0.628	8.41	9.23	8.57	8.74	0.435	0.251	1.67

Non-saturating conditions: C4-YSD 15nM, reaction of 5 minutes:

C4-YSD	OD BR1	OD BR2	OD BR3	V_0 1 (nM/min)	V_0 2 (nM/min)	V_0 3 (nM/min)	V0 average	STDEV	SEM	FC
**- PCBP1**	0.336	0.273	0.295	4.40	6.07	5.43	5.30	0.844	0.487	1.00
**+ PCBP1**	0.258	0.211	0.216	6.56	8.34	8.08	7.66	0.962	0.555	1.45

Saturating conditions: C4-YSD 500nM, reaction of 30 seconds:

C4-YSD	OD BR1	OD BR2	OD BR3	V_0 1 (nM/min)	V_0 2 (nM/min)	V_0 3 (nM/min)	V0 average	STDEV	SEM	FC
**- PCBP1**	0.673	0.646	0.655	8.16	9.33	8.74	8.74	0.585	0.338	1.00
**+ PCBP1**	0.669	0.624	0.652	8.32	10.41	8.87	9.2	1.083	0.625	1.05

Non-saturating conditions: Gmut-YSD 15nM, reaction of 30 seconds:

Gmut-YSD	OD BR1	OD BR2	OD BR3	V_0 1 (nM/min)	V_0 2 (nM/min)	V_0 3 (nM/min)	V0 average	STDEV	SEM	FC
**- PCBP1**	0.563	0.529	0.555	14.37	17.07	14.97	15.47	1.418	0.819	1.00
**+ PCBP1**	0.594	0.560	0.553	12.29	14.59	15.20	14.03	1.536	0.887	0.91

Non-saturating conditions: HSV120 dsDNA 15nM, reaction of 30 seconds:

HSV120 dsDNA	OD BR1	OD BR2	OD BR3	V_0 1 (nM/min)	V_0 2 (nM/min)	V_0 3 (nM/min)	V0 average	STDEV	SEM	FC
**- PCBP1**	0.803	0.999	0.809	4.66	1.57	4.12	5.11	3.589	1.794	1.00
**+ PCBP1**	0.805	1.009	0.777	4.62	1.50	4.84	4.79	2.744	1.372	0.94

Michaelis-Menten kinetic: C4-YSD, reactions of 30 seconds:

C4-YSD		V_0 1 (nM/min)	V_0 2 (nM/min)	V_0 3 (nM/min)	V0 average	STDEV	SEM
**2.5nM**	**- PCBP1**	0.11	0.11		0.11	0.001	0.001
	**+ PCBP1**	0.10	0.13		0.11	0.022	0.015
**7.5nM**	**- PCBP1**	4.32	3.74		4.03	0.412	0.291
	**+ PCBP1**	6.36	7.23		6.80	0.617	0.436
**15nM**	**- PCBP1**	4.83	5.14	5.72	5.23	0.223	0.129
	**+ PCBP1**	8.41	9.23	8.57	8.74	0.580	0.335
**50nM**	**- PCBP1**	7.84	9.71		8.77	1.324	0.937
	**+ PCBP1**	9.82	11.90		10.86	1.476	1.044
**500nM**	**- PCBP1**	8.16	9.33	8.74	8.74	0.827	0.478
	**+ PCBP1**	8.32	10.41	8.87	9.20	1.478	0.853

* Each V_0 is a different biological replicate, enzymatic reaction

Full unedited western blot pictures

Figure 2F

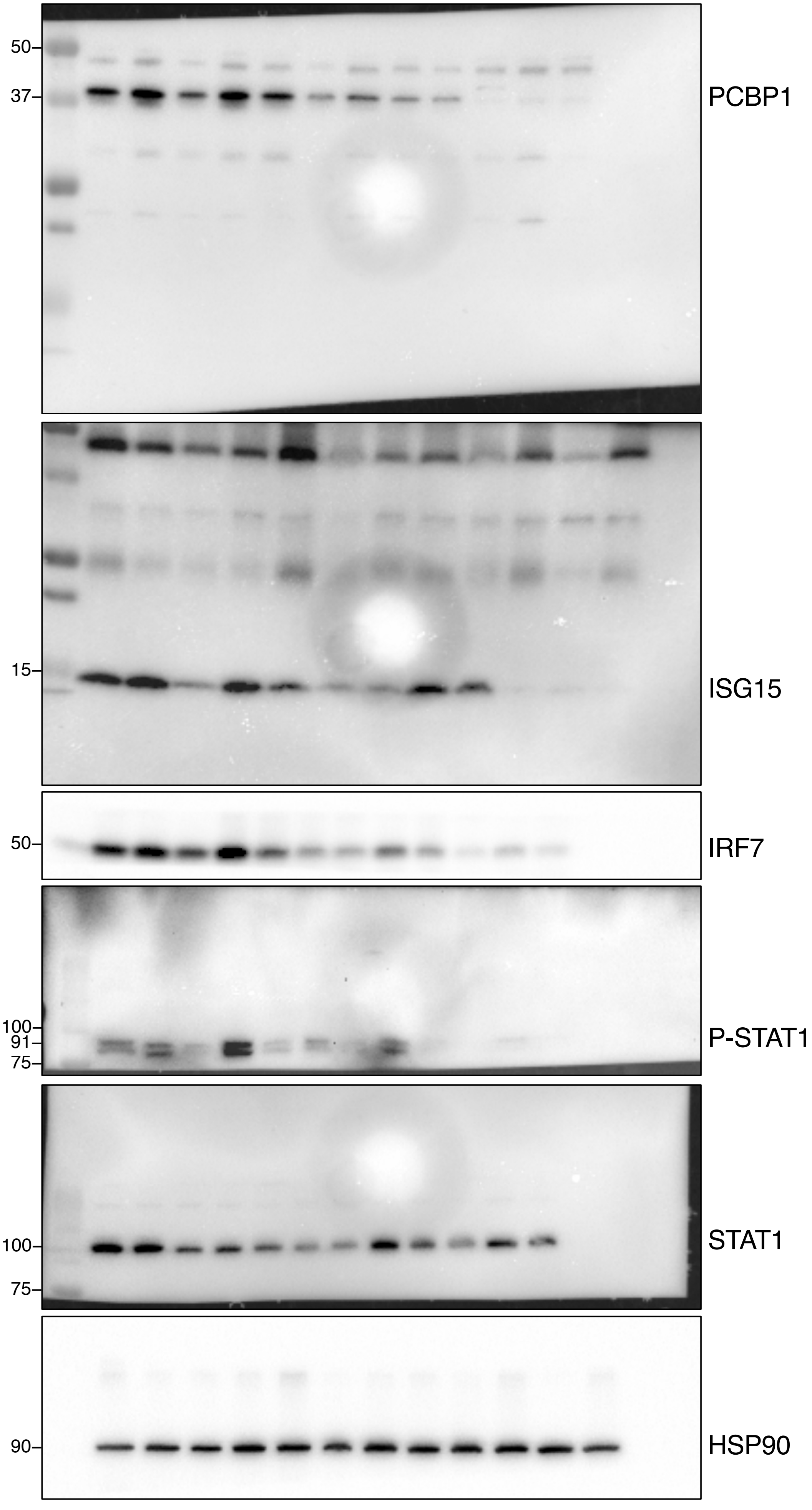

50
37
PCBP1
15
ISG15
50
IRF7
100
91
75
P-STAT1
100
75
STAT1
90
HSP90

Figure 3F

Py8119 cells

PCBP1

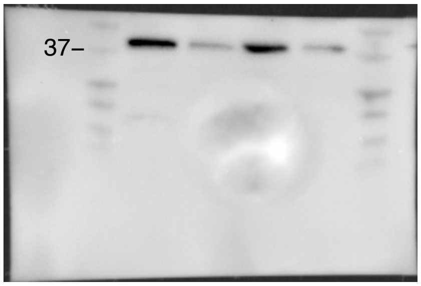

ISG15

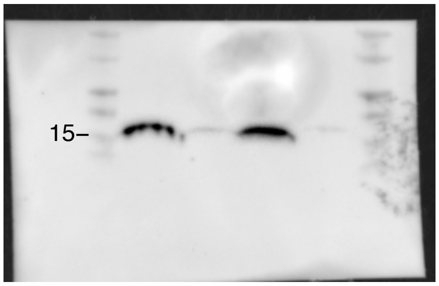

HSP90

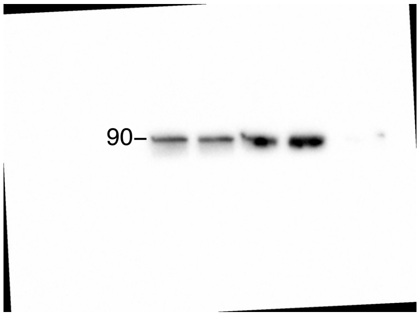

EMT6 cells

PCBP1

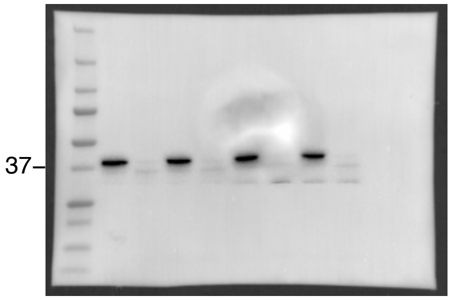

ISG15

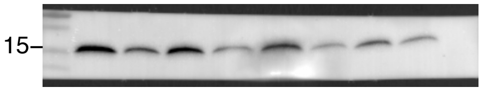

HSP90

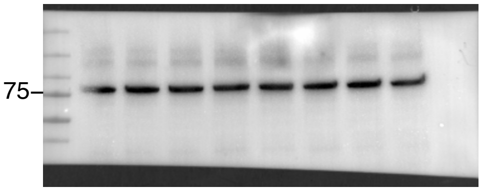

Figure 5B

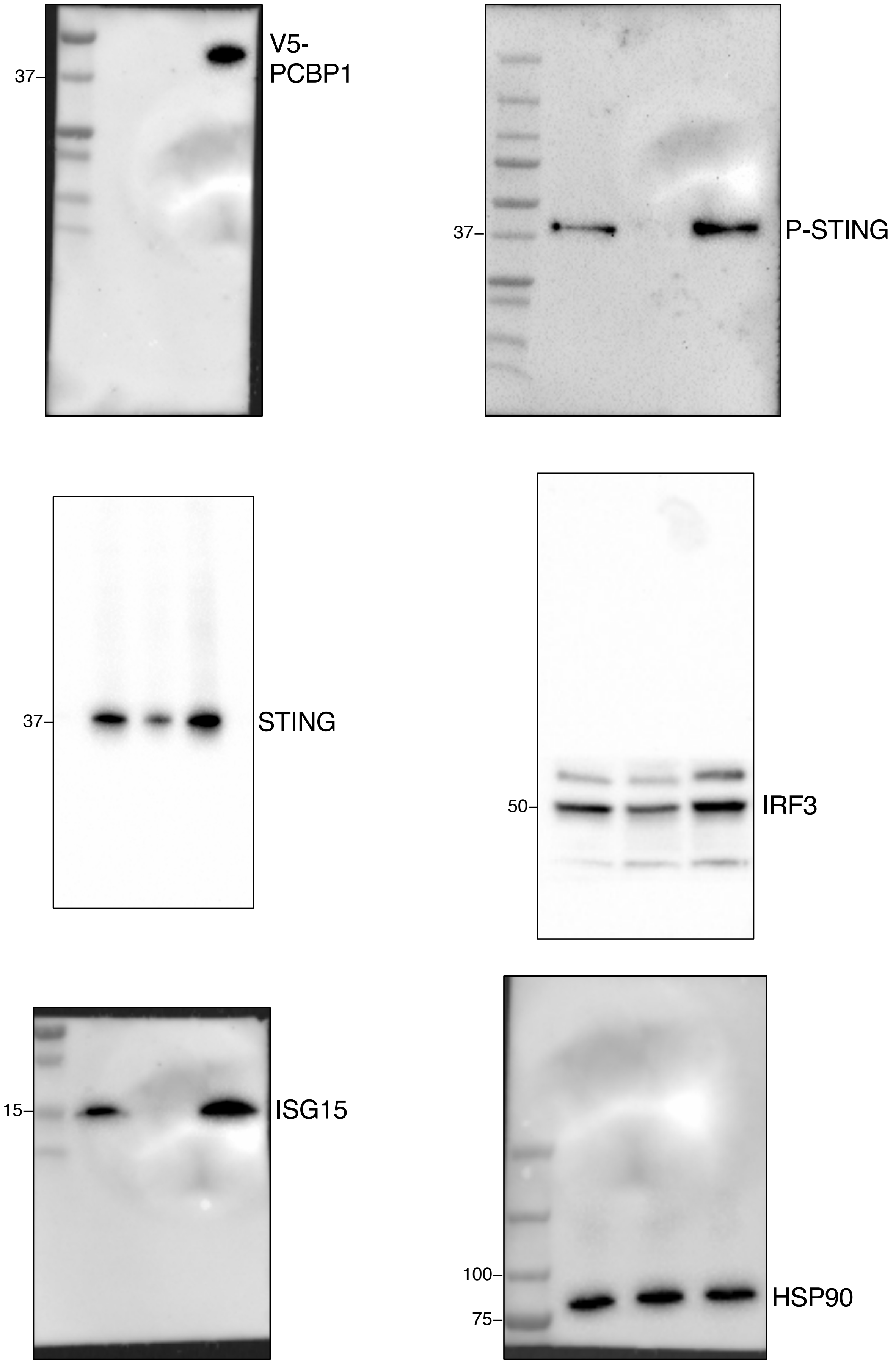

Figure 5C

EMT6 cells

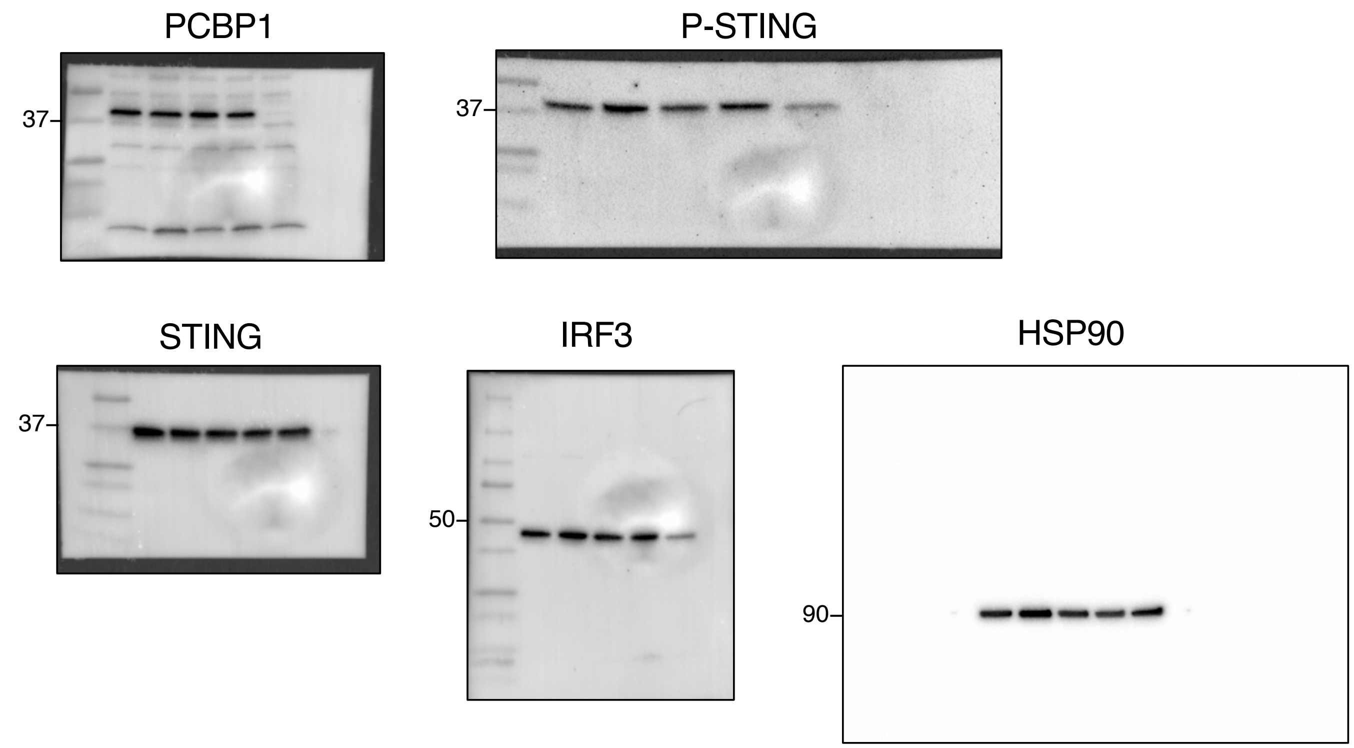

Figure 5D

HMLE cells

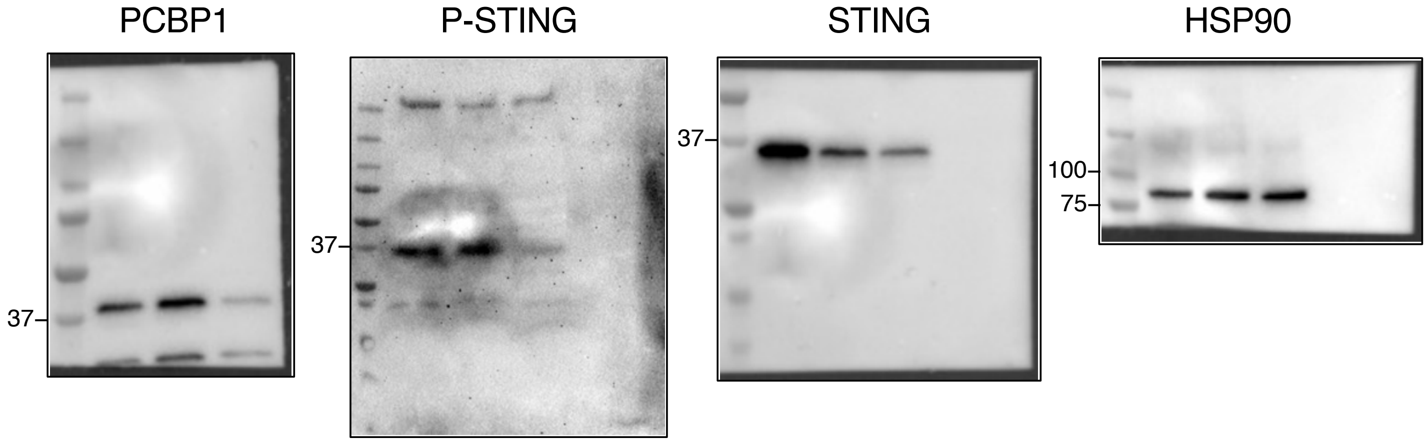

Figure 5G

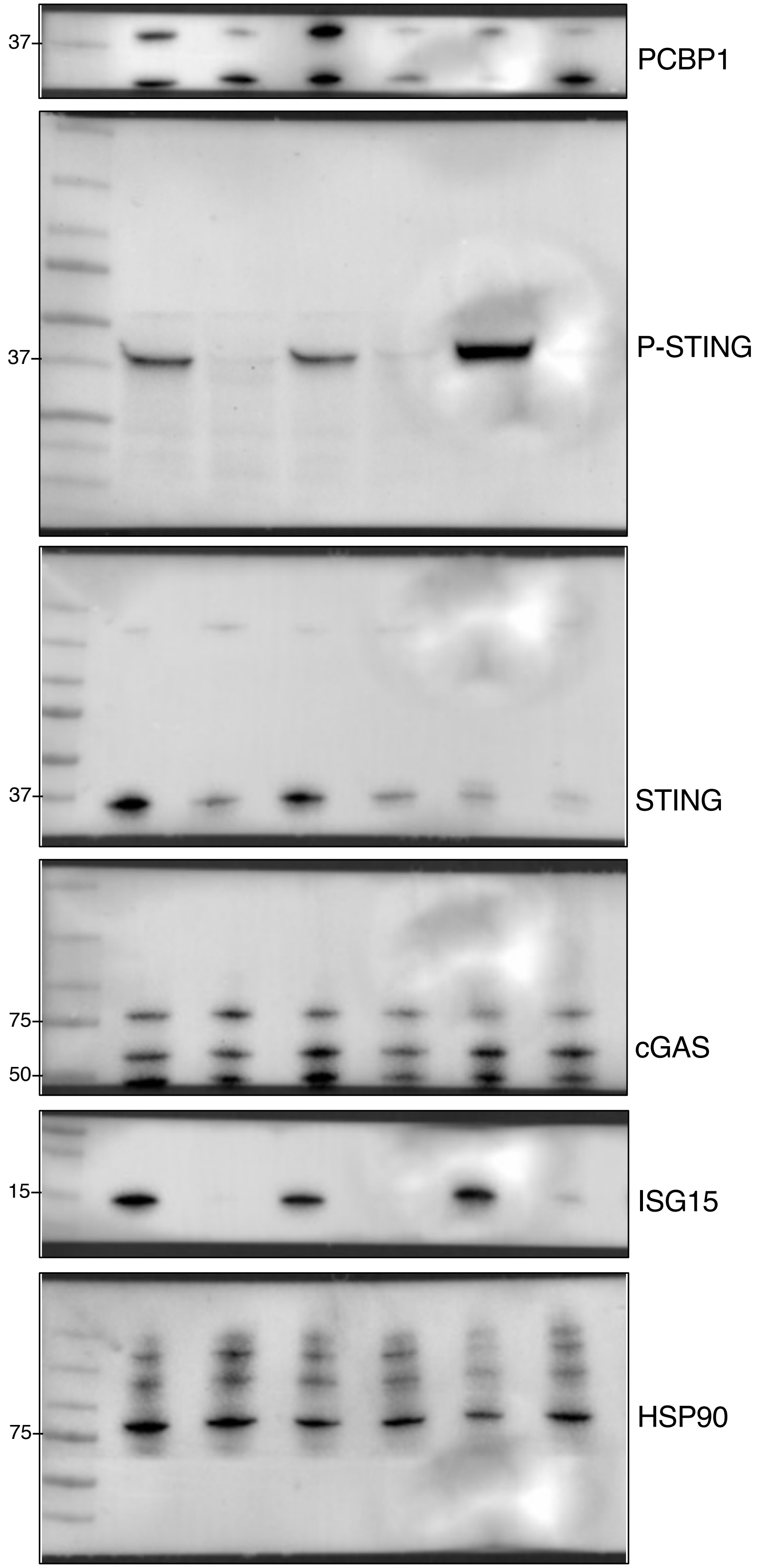

Figure 6

cGAS blot (inputs + pulldown)

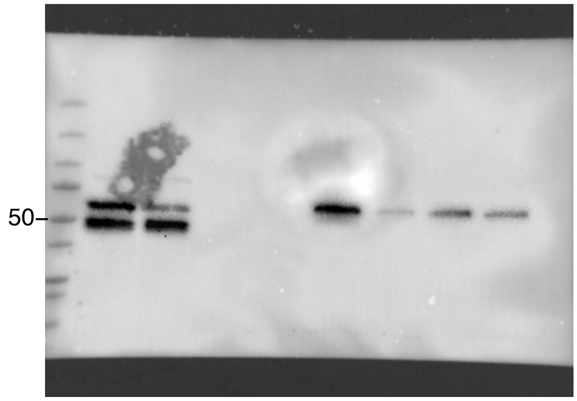

HSP90 blot (inputs + pulldown)

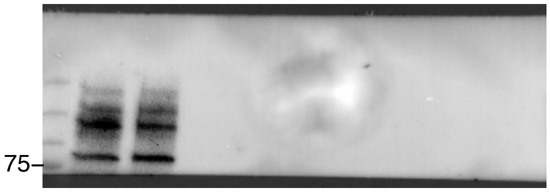

PCBP1 blot (inputs + pulldown)
with ladder

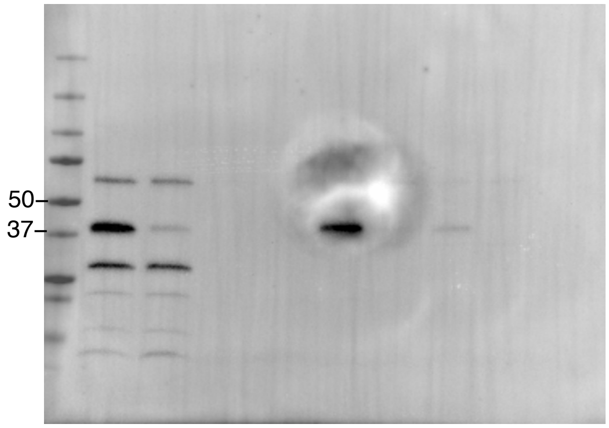

PCBP1 blot (inputs + pulldown)

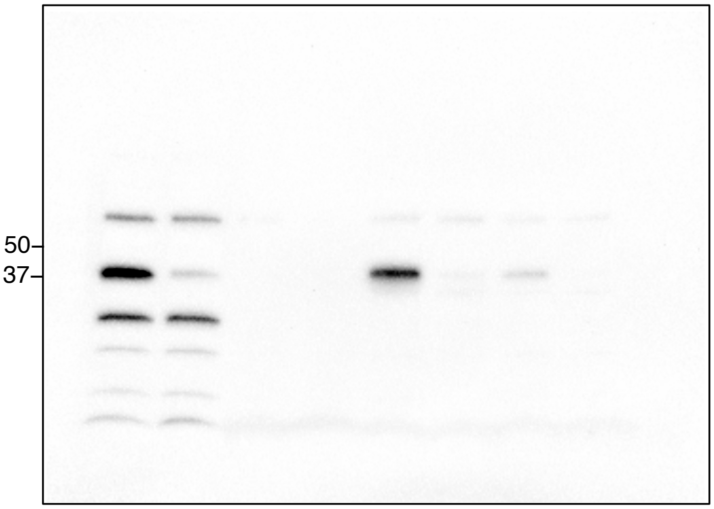

Figure 7

E

PCBP1 blot (inputs only)

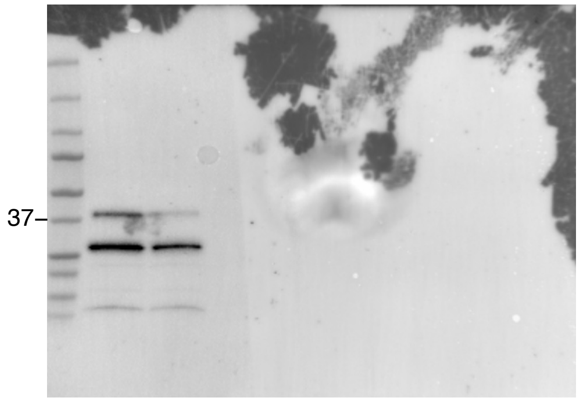

PCBP1 blot (inputs + pulldown)

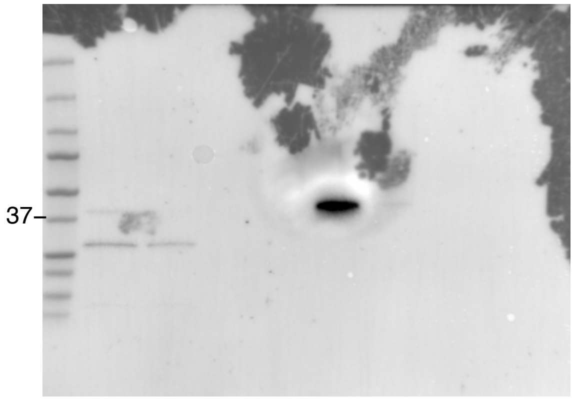

cGAS blot (inputs)

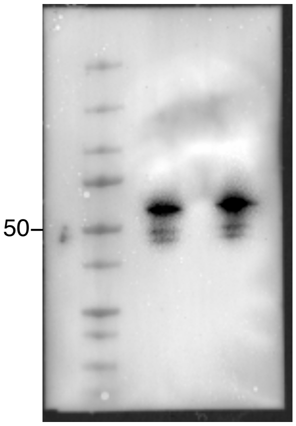

cGAS blot (pulldown)

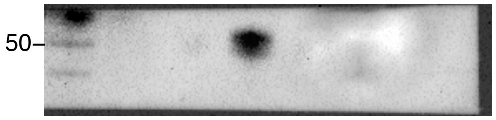

HSP90 blot (inputs + pulldown)

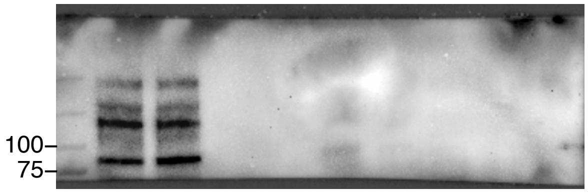

F

His blot (His-cGAS) with ladder

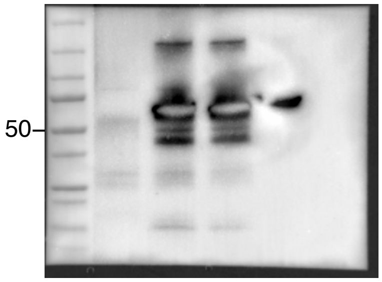

His blot (His-cGAS)

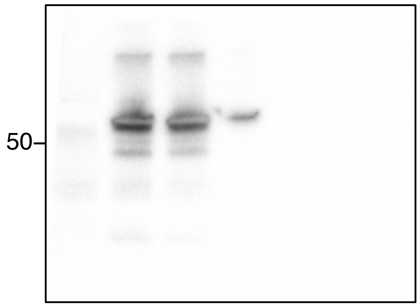

PCBP1 blot (GST-PCBP1) with ladder

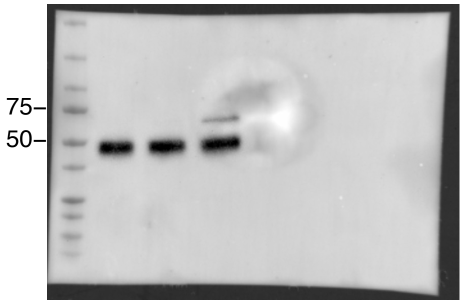

PCBP1 blot (GST-PCBP1) low exposure

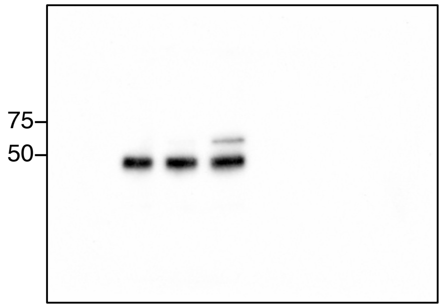

PCBP1 blot (GST-PCBP1) high exposure

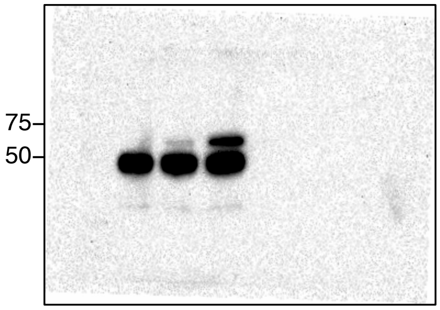

Supplementary Figure 4A

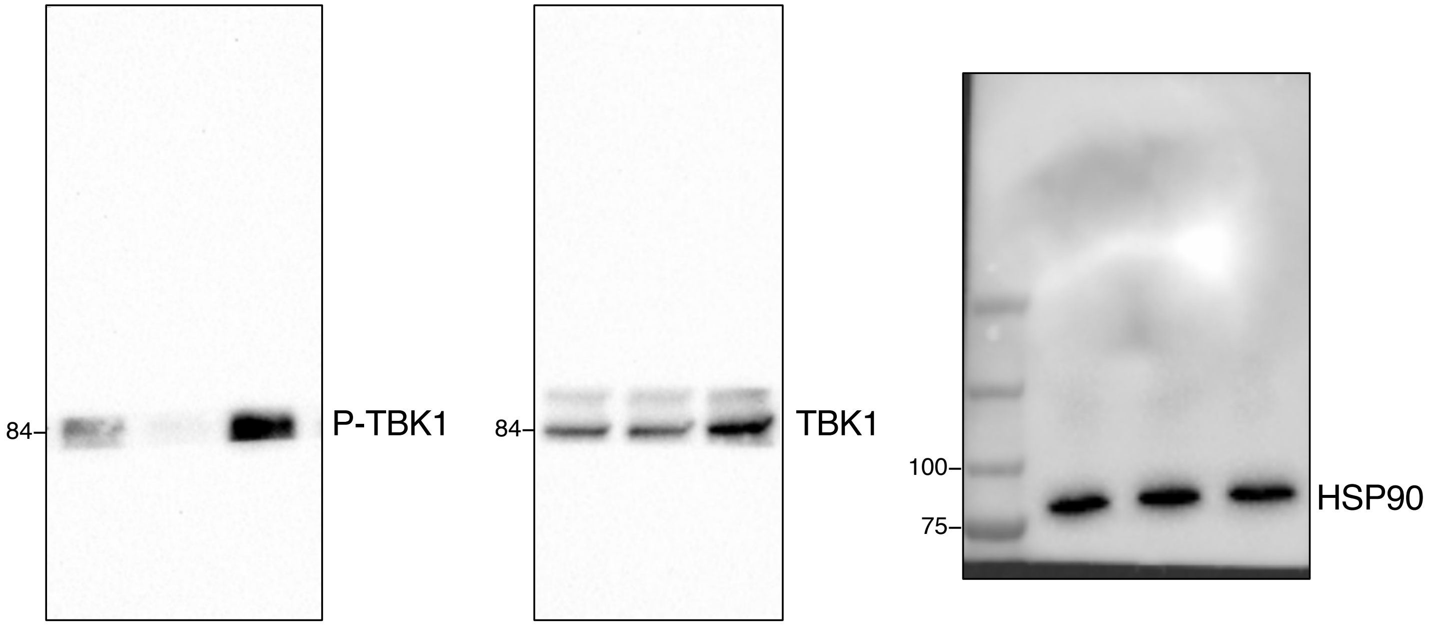

Supplementary Figure 4B

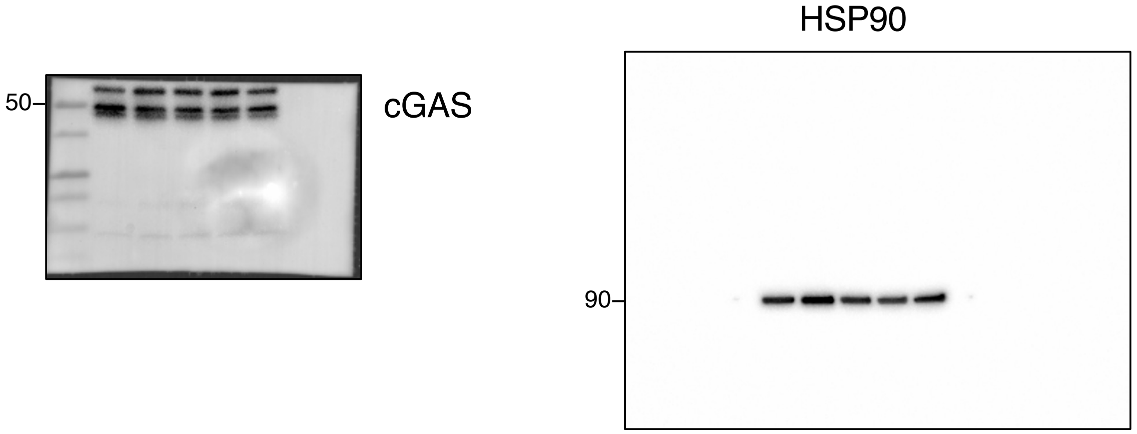

Supplementary Figure 4F

Py8119 cells

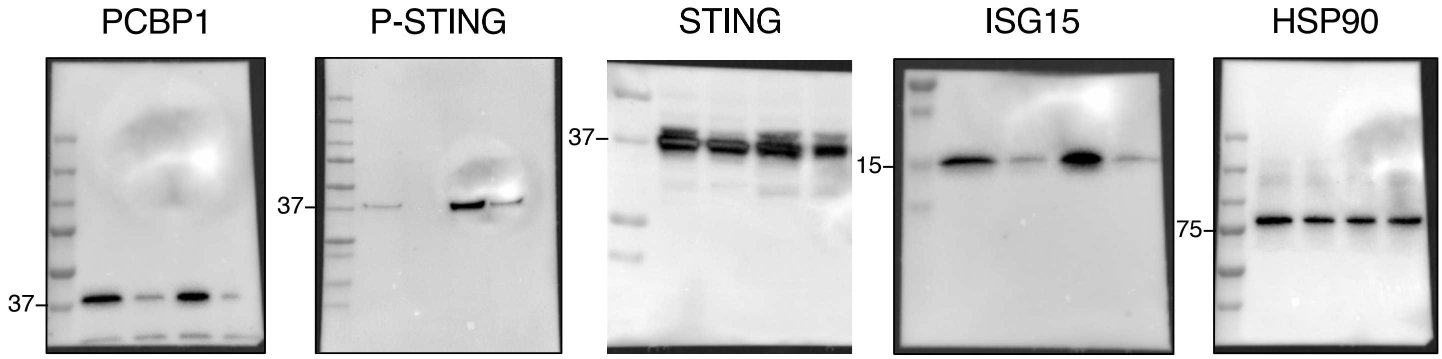

Supplementary Figure 4H

EMT6 cells

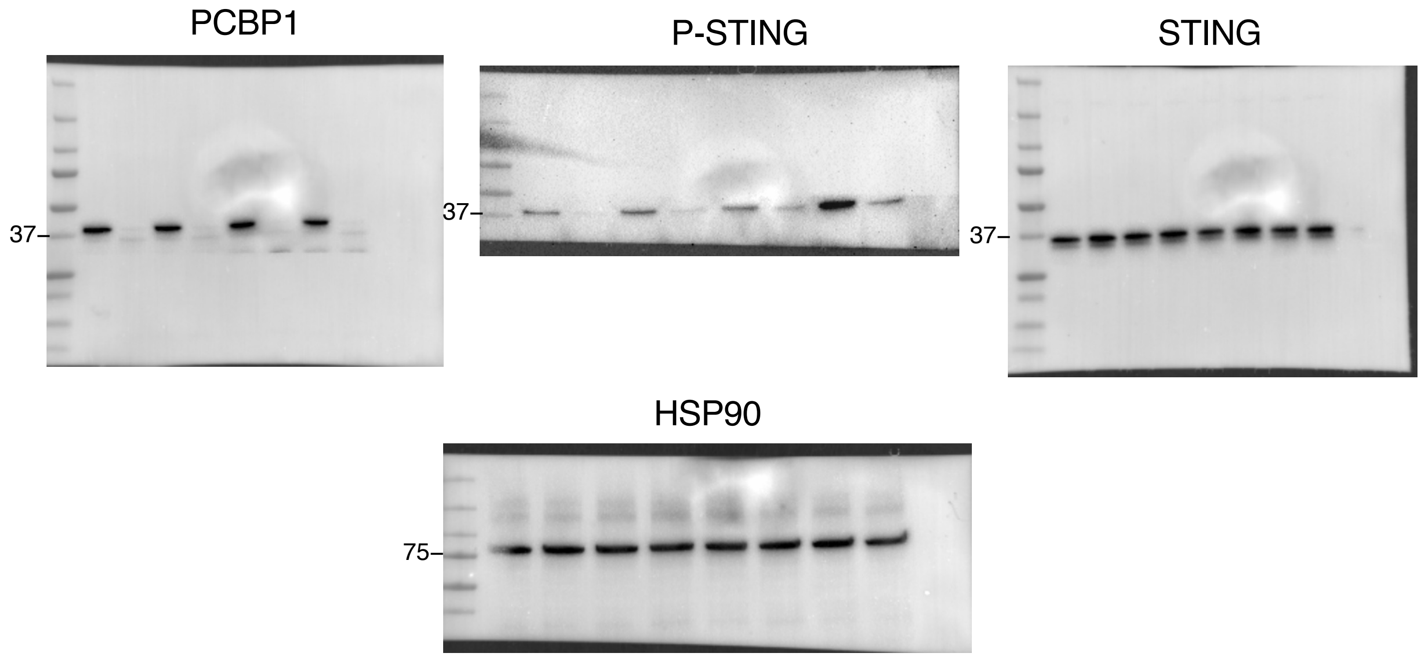

Supplementary Figure 5A

HSP90 blot, inputs + pulldown

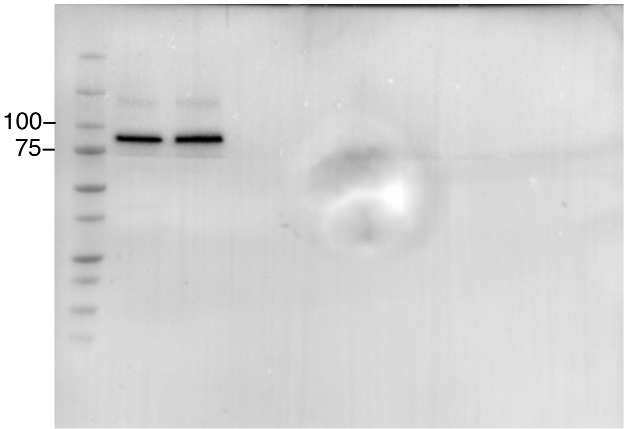

cGAS blot, inputs + pulldown

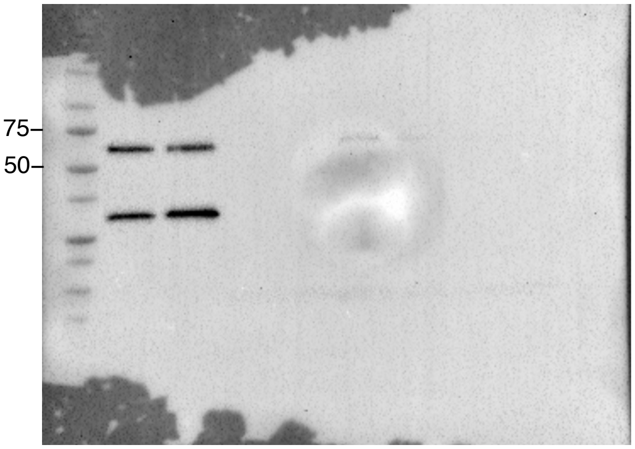

cGAS blot, pulldown (inputs hidden)

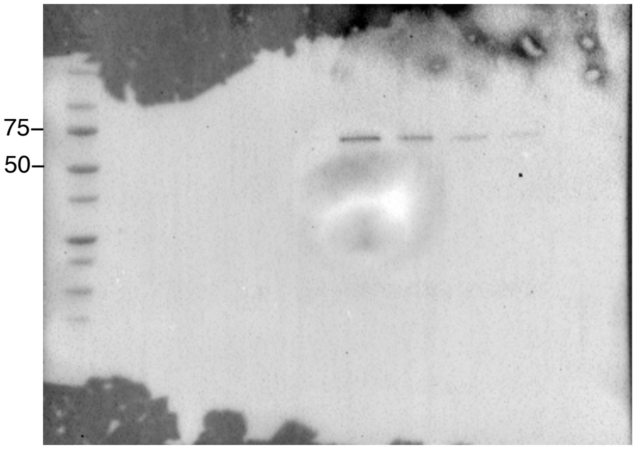

PCBP1 blot, inputs + pulldown

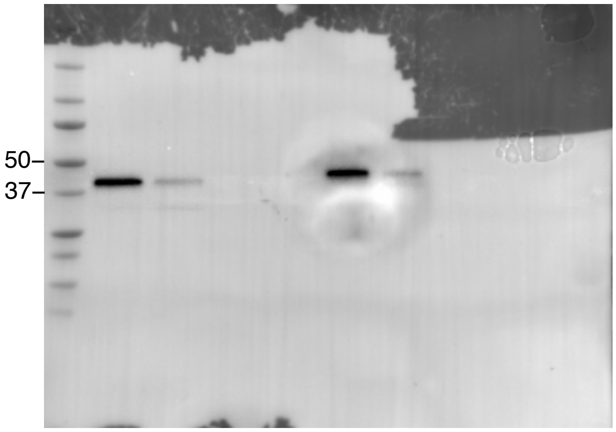

Supplementary Figure 5B-C-D

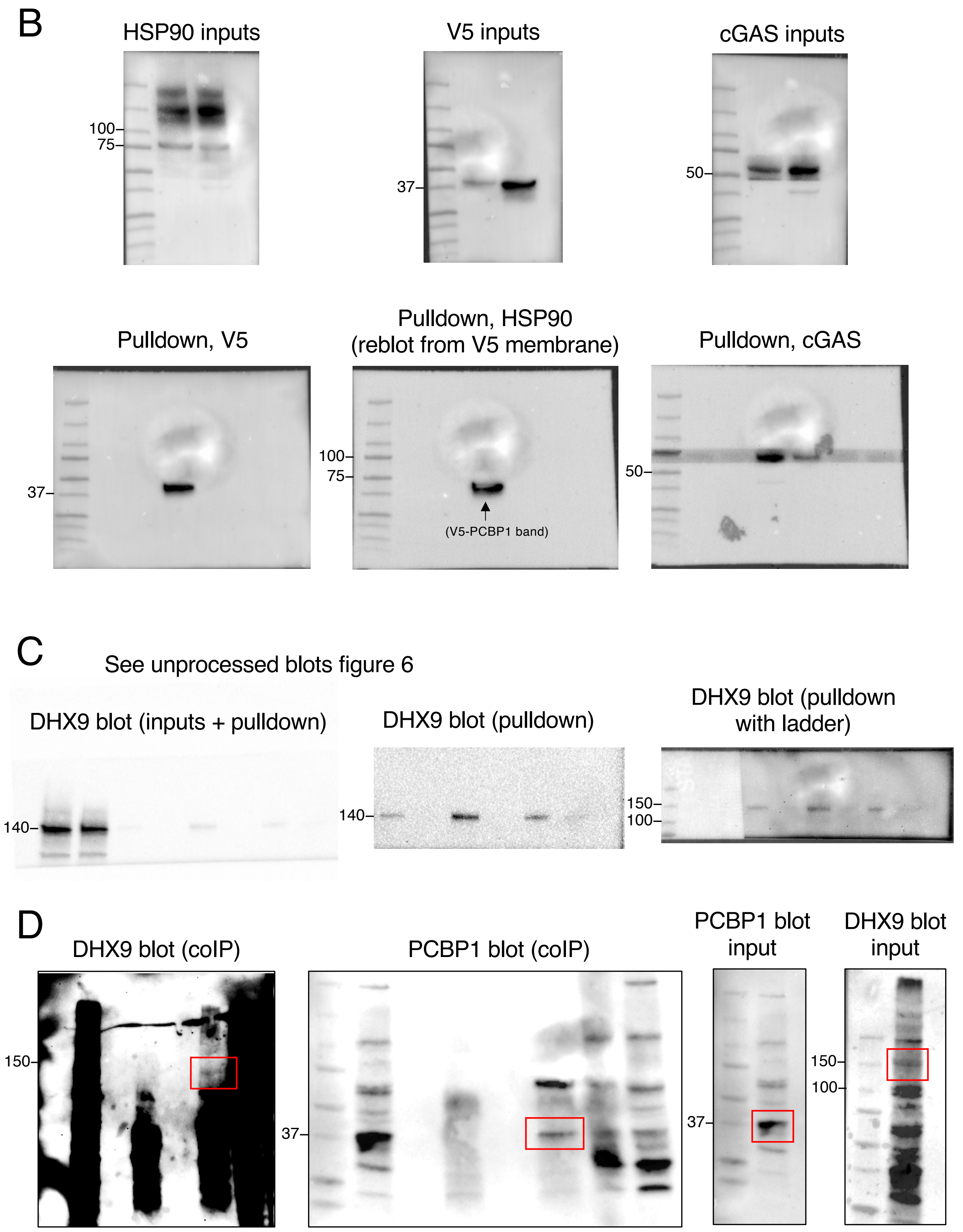

Supplement: Supplementary file 2 — Supplementary Information [file 42003_2025_9456_MOESM2_ESM.pdf]
