# Supplementary material for: PCBP1 binding to single-stranded poly-cytosine motifs enhances cGAS sensing and impairs breast cancer development
Source: Commun Biol. 2026 Jan 7;9:179. doi: 10.1038/s42003-025-09456-z (PMC12881503; doi:10.1038/s42003-025-09456-z)
Supplement: Supplementary file 3 — Description of Additional Supplementary File [file 42003_2025_9456_MOESM3_ESM.pdf]

Description of Additional Supplementary Files

File name: Supplementary Data

Description: Excel file containing all raw data
