# Supplementary material for: PCBP1 binding to single-stranded poly-cytosine motifs enhances cGAS sensing and impairs breast cancer development
Source: Commun Biol. 2026 Jan 7;9:179. doi: 10.1038/s42003-025-09456-z (PMC12881503; doi:10.1038/s42003-025-09456-z)
Supplement: Supplementary file 1 — Transparent Peer Review file [file 42003_2025_9456_MOESM1_ESM.pdf]

nature portfolio

Peer Review File

PCBP1 binding to single-stranded poly-cytosine motifs enhances cGAS sensing and impairs breast cancer development

Corresponding Author: Ms Cécile Fréreux

This file contains all reviewer reports in order by version, followed by all author rebuttals in order by version.

Version 0:

Reviewer comments:

Reviewer #1

(Remarks to the Author)
Title: PCBP1 promotes anti-tumor immunity by mediating single stranded DNA activation of the cGAS-STING pathway in breast cancer cells.

In this study, the authors demonstrated that PCBP1 loss significantly increases mammary tumor burden, impairs type I IFN and ISG expression, and reduces cytotoxic T cell infiltration in a C57BL/6 MMTV-PyMT MMTV-Cre PCBP1(fl/fl) mouse model. Furthermore, they linked these effects to the activation of the cGAS-STING pathway, which appears to be dependent on PCBP1 expression.

Overall, the study provides sufficient evidence for the importance of PCBP1 in tumor development and its role in the regulation of interferon and ISG expression. Additionally, it establishes a link between PCBP1 and the activation of cGAS in cancer cells. However, it remains unclear whether ssDNA, in the presence of PCBP1, can directly activate cGAS. To strengthen this conclusion, additional experimental evidence is needed. Below are specific comments and recommendations for addressing this issue:

1. Lines 78-82: The statement "RNA and various nucleic acid-binding proteins have been implicated in modulating the cGAS-STING pathway" lacks references. Please provide appropriate citations to support this claim, as well as other statements in this paragraph.
2. Clarification of the Mouse Model: For better understanding, please provide more details on the MMTV-PyMT mouse model, including its relevance to the study.
3. Figure 2F: The experiment appears to include 12 conditions/samples, yet the IRF7 blot contains 13 bands. Please clarify and correct this discrepancy. Additionally, the pSTAT1 blot seems to have 11 bands—please verify and adjust accordingly.
4. Figures 3 and 4: The number of experimental replicates is not indicated. Please specify how many independent experiments were performed in the figure legends. In the Materials and Methods section, the authors state that some experiments were conducted twice, but a minimum of three independent replicates is necessary for statistical robustness.
5. Figure 3E-F (PCBP1 KO Validation): The authors present Py8119 PCBP1-/- cells, generated via CRISPR-Cas9. However, in the Western blot, a PCBP1 band is still visible. Please clarify whether the KO is complete, if residual protein expression remains, or if this represents an incomplete knockout.
6. Figure 4D and 4E (Vehicle Control and cGAMP Production):
• The identity of the vehicle control is not specified. Please clarify what substance was used.
• The vehicle appears to induce cGAMP production, which is impaired in KO cells. Why does this occur?
• A negative control is missing: The authors should show cGAMP levels in untreated cells (without vehicle or ssDNA).
• While there is a clear difference in cGAMP production between WT and KO cells, this difference is also observed with the vehicle treatment, not only with ssDNA stimulation. Please clarify this observation.
• The difference between ssDNA and vehicle-induced cGAMP production is minor, which raises concerns about whether ssDNA truly activates cGAS-STING in the presence of PCBP1.
• To strengthen the argument, the authors should measure cytokine production (e.g., IFN-β, ISGs) via ELISA or qPCR to confirm that ssDNA in the presence of PCBP1 can activate the pathway.
• To provide definitive evidence, the authors should perform experiments using either a cGAS knockdown (KD) or knockout

(KO) (or sting). This would confirm whether ssDNA-mediated cGAS activation truly requires PCBP1.
• Please indicate whether cGAMP was measured in the cell supernatant or cell lysates.
7. Figure 5E (dsDNA Results): The manuscript does not discuss the results shown in Figure 5E (dsDNA condition). Please provide an explanation and interpretation of these findings.
8. Figure 5F (Biotin-RNA vs. DNA): The authors use biotinylated RNA, yet the central hypothesis focuses on ssDNA-mediated activation of cGAS. Why was RNA used instead of DNA? The authors should repeat this experiment using biotinylated DNA to align with their hypothesis.
9. Figure 6E (Legend Formatting): The label "E" should be bold in the legend for consistency.
10. Possible Explanation for ssDNA Activation of cGAS: It is possible that ssDNA forms secondary structures, allowing it to interact with cGAS. The authors should consider discussing this as a potential explanation in the Discussion section.

Reviewer #2

(Remarks to the Author)

In this manuscript, Fréreux and colleagues investigate the role of Poly(rC) Binding Protein 1 (PCBP1) in antitumor immunity in breast cancer. Using immunocompetent mammary tumorigenesis mouse models and murine in vitro breast cancer models, they show that PBCP1 depletion curbs baseline expression of interferon (IFN)-stimulated genes (ISGs) within malignant cells, enhances tumor growth and is associated with a broadly immunogenically colder, cytotoxic T cell-depleted tumor microenvironment. Through mechanistic in vitro dissection of PCBP1's mode of ISG activation, the authors propose that PCBP1's function as a poly(C)-rich region-binding protein facilitates single-stranded DNA (ssDNA)-mediated activation of cGAS and the downstream STING–interferon pathway, linking its canonical role to the authors' observed immune phenotypes.
The characterization of in vivo PCBP1-/- immunophenotypes is very well-executed and the authors' conclusions are validated using multiple orthogonal approaches, including bulk and single-nuclei transcriptome sequencing, as well as flow cytometric and immunohistochemical analyses.

The dissection of the mechanistic link between PCBP1 and cGAS activation is weaker and more reliant on circumstantial evidence. Different cell lines, including murine breast cancer and human mammary epithelial cells, are profiled through immunoblotting and/or RT-qPCR to assess (STING pathway-associated) type I IFN activation markers in PCBP1 depletion contexts. However, which cell lines are profiled using which querying method and which markers are used to evaluate activation is inconsistent throughout experiments and the reason for said inconsistencies and why certain lines are chosen over others is not provided.
The evidence for the proposed PCBP1-mediated and selective binding of cGAS to ssDNA is reliant predominantly on REMSA / EMSA and cGAMP ELISA read-outs. This link, especially, would benefit from further strengthening through, for instance, additional profiling of PCBP1-dependent ssDNA-driven cGAS activation or pull-down assays with additional nucleic acid species beyond BAT RNA.

Some comments to consider.

Related to figures:

Fig. 2A. The y-axis cut-off on the volcano plot implies a p-value cut-off of 0.001, whereas the text mentions a cut-off of 0.0001.

Fig. 2B. The text mentions 17/20 shown genes are ISGs, though these are not clearly annotated.

Fig. 2D. Plots are grainy and hard to read.

Fig. 2E, 3E and similar. Add Y-axis label rather than "(F.C.)" above figures.

Fig. 2F. There is no mention of hets in the main text. Densitometric analysis of blots might show a progressive decrease in IFN pathway components across the three genotypes.

Fig. 2G. This could benefit from a zoomed in area of interest to highlight pronounced selective downregulation in mammary cancer cells, which the main text highlights.

Fig. 3B. Whilst this representation shows this quite clearly for the two selected ISGs (Isg15, Oas2), it is not an ideal representation of the data, as it's not clear how many cells of the luminal epithelial mammary gland cluster were contributed by each genotype and, hence, what the relative proportion of selected ISG-expressing cells is for each genotype. A heatmap similar to what the authors use in Figure S1A (showing both average expression and % expression) would be more effective and would allow inclusion of more ISGs beyond (Oas2 and Isg15), as well as better assessment of cell cluster selectivity of ISGs.

Fig. 3C. It is not clear what has led to certain genes being assigned one cell type over another. What analysis was performed to enable the highlighting of ISGs as mammary epithelial cell specific? This needs a more detailed explanation in either text or figure legend.

Fig. 3E. Need to demonstrate PCBP1 O/E (for PCBP1-/- V5-PCBP1) by immunoblot alongside PCBP1-/- and PCBP1+/+ samples. Can the increase in ISGs/IFNB1 in V5 be ascribed to O/E of PCBP1 beyond endogenous levels?

Fig. 4B, C; S2A, B, C. All phospho-protein blots need matched whole-protein blots (e.g. p-STAT1, but no STAT1 in 4B; p-STING but no STING in 4B). Inconsistencies in the order in which blotted proteins are displayed between 4B and 4C affects legibility. Some used markers, such as IRF3, TBK1 and cGAS by themselves are not good indicators of cGAS-STING pathway activation.

Fig. 1C, 2G, 6D. Scale bars needed on all ICC images.

Additional comments:

The n numbers on experiments are often not clear – especially where representative images have been used (e.g. immunoblots) or barplots have been used for data presentation (e.g. RT-qPCRs).

The authors refer twice in the main text to the use of human breast cancer cells in the study. However, HMLE is as far as I'm aware the only human line used and is a non-cancerous epithelial line.

Version 1:

Reviewer comments:

Reviewer #1

(Remarks to the Author)
Comment:
• Page 5: Similar results were obtained in the EMT6 breast cancer cell line using shRNA-mediated PCBP1 silencing, with significant downregulation of IFIT1 and IFNB1 (Fig. 3E).
However, in the figure there are not data shown for IFNB in EMT6 cells.

Reviewer #2

(Remarks to the Author)
In this revised version of their manuscript, Fréreux and colleagues include significant experimental additions addressing, in particular, reviewer concerns centred around the initially proposed mechanism of PCBP1-mediated recruitment of cGAS to ssDNA.
The main approach(es) taken to address reviewer concerns is using various oligonucleotide species (HSV120 ss/dsDNA, BAT RNA, G3/C4-YSD) in in vitro pull-down assays and EMSAs.
These revision experiments significantly clarify the mechanistic part of the manuscript and have resulted in an updating of their previous mechanistic model to a more nuanced one: wherein PCBP1 helps recruitment of cGAS to (ds)DNA comprising poly-cytidine ssDNA tracts (e.g. poly-C overhangs).

In addition to added experimental data, other changes made to the text (points of clarification), figures (replotting to reflect underlying sample sizes; addition of a hypothetical model figure / Fig.8) and figure legends (clarification of sample sizes and stats) have resulted in a substantial improvement of the manuscript.

Remaining comments:

1) Line 110-111: "The downregulation of ISG expression, including ISG15, IRF7, IFIT1, Rsad2, and Bst2, as well as IFNB1, was validated by qPCR in PyMT PCBP1–/– tumors (Fig. 2E)."

The murine naming convention (Isg15, Irf7, …) should apply consistently to all mouse genes.

2) Enrichment plots in Fig. 2D and 4B should show statistical outputs (NES, adjusted P/FDRq)

3) Whilst the used sample sizes for each experiment are now much clearer thanks to the authors' replotting of a lot of the data, it is still not clearly stated how many independent experiments the images of immunoblots and EMSAs are representative of.

4) A repetition of a previous point: All phospho-protein blots need matched whole-protein blots and vice-versa for phospho-indicators of cGAS-STING pathway activation (IRF3, TBK1, STING, STAT1).

For instance:

Fig.S4D – No whole-protein STING to go with p-STING.

Fig. S4F – No p-TBK1 to go with TBK1 whole-protein.

Fig. 5B-C – Whole-proteins without activation marks (IRF3) or activation marks (phospho) without whole protein (e.g. p-STAT1).
In some instances (e.g. Fig.5B), slimming down blots by removing such 'unmatched' and inconsistently used markers would improve clarity across figures.

5) Line 229-231: "cGAS was titrated in the presence of a constant amount of PCBP1, the PCBP1/oligo complex disappeared and a super shifted complex appeared, consistent with simultaneous binding of both proteins to the same ssDNA molecule (Fig. 6A left panel).

Should this not say "Fig. 6A right panel"?

6) Line 236 and other: Should refer to "Fig. S5" not "S4" – ditto for other mentions of 'Fig.S4'.

7) Line 235: Compared to the previous version of the manuscript, this version does not introduce or define 'BAT RNA'. Doing so would help clarify those data.

8) Reference 65 and 66 in the text map to references 66 and 67 in the reference list, respectively. It is worth checking whether this mismatch isn't more pervasive across in-text references.

9) Line 273-277: It is not entirely clear on first pass that the ultimate read-out used for the kinetics assay is cGAMP ELISA-based. It is worth underscoring this in the text.

10) Fig.7E and Fig. S6A-C – It is unclear what cell lines / lysates pull-downs were performed on. Please clarify in the legends.

11) The C4-YSD oligo created by the authors seems to have very low inherent affinity for cGAS (Fig.7B and E). This chimes with results from the original study which derived G3-YSD (doi: 10.1038/ni.3267), showing very little interferon induction upon transfection with a C3-YSD (polyC overhang) variant of the oligo.

Can the authors clarify the use of this oligo species with low cGAS affinity and how it relates to their model? Were any experiments done to test its functionality as a cGAS agonist? Do they suggest PCBP1 mediates binding of cGAS to nucleic acid substrates cGAS would otherwise have low or no affinity to for (increased repertoire), or that it broadly improves affinity to all its substrates?

Version 2:

Reviewer comments:

Reviewer #2

(Remarks to the Author)
I kindly thank the authors for their efforts in revising the manuscript.

Their revised manuscript represents a thorough and multi-disciplinary study of how the ssDNA-binding protein PCBP1 is involved in innate DNA recognition through cGAS.

Their multi-pronged approach, looking at both (1) the mechanistic basis of PCBP1's co-sensor capacity using various in vitro / biochemistry approaches and (2) exploring the broader tumor microenvironmental / biological consequences of PCBP1's functon(using in vivo models) is comprehensive and thorough.

Their results represent important contributions to the cGAS-STING/innate sensing field that further add to our understanding of the numerous complex regulatory programs governing innate sensing, as well as the drivers and outcomes of innate sensing in (sterile inflammatory) cancer settings.

Open Access This Peer Review File is licensed under a Creative Commons Attribution 4.0 International License, which permits use, sharing, adaptation, distribution and reproduction in any medium or format, as long as you give appropriate credit to the original author(s) and the source, provide a link to the Creative Commons license, and indicate if changes were made.
In cases where reviewers are anonymous, credit should be given to 'Anonymous Referee' and the source.
The images or other third party material in this Peer Review File are included in the article's Creative Commons license, unless indicated otherwise in a credit line to the material. If material is not included in the article's Creative Commons license and your intended use is not permitted by statutory regulation or exceeds the permitted use, you will need to obtain permission directly from the copyright holder.
To view a copy of this license, visit https://creativecommons.org/licenses/by/4.0/

Changes made to the manuscript for each figure:

All legends/text callouts updated; n and statistics specified where changed. No datasets removed unless noted.

Title was changed

Figure 1

- **A**: Corrected Kaplan–Meier after removing duplicate samples flagged by UCSC Xena; updated n and P.
- **C**: Updated microscopy; added scale bars.
- **F**: Added individual replicate values.
- **B, D, E**: Unchanged.
- Legend: unchanged.

Figure 2

- **A–D**: Unchanged.
- **E**: Added individual replicate values.
- **F**: Fixed layout (per Reviewer 1).
- **G**: Updated images; added scale bars and zoom insets.
- **I–J**: New panels.
- Legend: updated (n/stats; I–J added).

Figure 3

- **A–C**: Globally unchanged (color tweaks); macrophages split into **M1-like** and **M2-like** by snRNA-seq.
- **D**: Unchanged.
- **E**: Added individual replicate values.
- **F**: Removed Py230 line for consistency.
- Legend: clarified n and stats.

Figure 4 *(previously Fig. 6; re-ordered for flow)*

- **A**↔**B**: Panels inverted; **A** expanded to include additional MHC-I genes.
- **C**: Unchanged.
- **D**: Added scale bars.
- **E**: Added individual replicate values; restyled graph.
- **F–I**: New panels (PCBP1/IFN → CXCL9/10 → $CD8^+$ T-cell infiltration).
- Legend: updated.

Figure 5 *(previously Fig. 4)*

- **A**: Added individual replicate values.
- **B–C**: Added total protein blots.
- **D**: Moved from former Suppl. Fig. S2A (human cells now shown in main).
- **E–G**: New; replace prior D–E. Mechanistic clarification
- Legend: updated.

Figure 6 *(previously Fig. 5; major re-org, no datasets removed)*

- BAT RNA EMSAs moved to Supplement.
- **B**: New schematic.
- **C**: From former Suppl. Fig. S4D.
- **D**: Retained (relabeled).
- **E**: Re-plotted EMSA quantifications for readability.
- **F**: New data.

- Legend: updated.

Figure 7
- Entirely new figure (new data) + legend/text.

Figure 8
- New summary schematic + legend/text.

Supplementary Fig. S1
- **A**: Updated expression plot with revised annotations.
- **B**: New (addresses Reviewer 2, Q7).
- **C**: New (addresses Reviewer 2, Q9).
- Legend: updated.

Supplementary Fig. S2
- New: flow cytometry gating strategy + legend/text.

Supplementary Fig. S3
- Images updated; added scale bars.

Supplementary Fig. S4
- **A–B**: New.
- **C–F**: As before, now with individual replicate values.
- Legend: updated.

Supplementary Fig. S5
- Reorganization; BAT RNA data from former main Fig. 5 moved here.

Supplementary Fig. S6
- New figure (new data).

Supplementary table 1: oligos/primers added
Supplementary table 2: new addition (replaces previous figure S1B)
Supplementary tables 3: new addition (supports figure 7)
All unedited blots have been added in supplementary data

Reviewer #1 (Remarks to the Author):

Title: PCBP1 promotes anti-tumor immunity by mediating single stranded DNA activation of the cGAS-STING pathway in breast cancer cells.

In this study, the authors demonstrated that PCBP1 loss significantly increases mammary tumor burden, impairs type I IFN and ISG expression, and reduces cytotoxic T cell infiltration in a C57BL/6 MMTV-PyMT MMTV-Cre PCBP1(fl/fl) mouse model. Furthermore, they linked these effects to the activation of the cGAS-STING pathway, which appears to be dependent on PCBP1 expression.

Overall, the study provides sufficient evidence for the importance of PCBP1 in tumor development and its role in the regulation of interferon and ISG expression. Additionally, it establishes a link between PCBP1 and the activation of cGAS in cancer cells. However, it remains unclear whether ssDNA, in the presence of PCBP1, can directly activate cGAS. To strengthen this conclusion, additional experimental evidence is needed. Below are specific comments and recommendations for addressing this issue:

1. Lines 78-82: The statement "RNA and various nucleic acid-binding proteins have been implicated in modulating the cGAS-STING pathway" lacks references. Please provide appropriate citations to support this claim, as well as other statements in this paragraph.
This does not appear in the text anymore since we have made significant changes to the manuscript.

2. Clarification of the Mouse Model: For better understanding, please provide more details on the MMTV-PyMT mouse model, including its relevance to the study.
We added a paragraph to explain the MMTV-PyMT model and relevance for the study of TNBC:
Line 83 "To further investigate the anti-tumorigenic role of PCBP1, we generated a C57BL/6 transgenic immunocompetent mouse model expressing the MMTV-PyMT (polyomavirus middle T antigen) oncogene, which drives mammary tumorigenesis. The MMTV-PyMT model is widely used in breast cancer research as it recapitulates the histological and molecular progression of human breast cancer patients with the development of multifocal adenocarcinoma and metastatic lesions to the lungs. As tumors progress, they lose estrogen and progesterone receptor and often gain androgen receptor expression, making this model suitable for studying triple-negative breast cancer (TNBC), particularly the luminal AR (androgen receptor–positive) TNBC subtype"

3. Figure 2F: The experiment appears to include 12 conditions/samples, yet the IRF7 blot contains 13 bands. Please clarify and correct this discrepancy. Additionally, the pSTAT1 blot seems to have 11 bands—please verify and adjust accordingly.
The layout issue in Figure 2F has been corrected.

4. Figures 3 and 4: The number of experimental replicates is not indicated. Please specify how many independent experiments were performed in the figure legends. In the Materials and Methods section, the authors state that some experiments were conducted twice, but a minimum of three independent replicates is necessary for statistical robustness.
All experiments now have a minimum of three biological replicates or more.

5. Figure 3E-F (PCBP1 KO Validation): The authors present Py8119 PCBP1-/- cells,

generated via CRISPR-Cas9. However, in the Western blot, a PCBP1 band is still visible. Please clarify whether the KO is complete, if residual protein expression remains, or if this represents an incomplete knockout.

The CRISPR-Cas9–mediated knockout of PCBP1 in Py8119 cells indeed resulted in a partial loss of protein expression. We have repeatedly attempted to generate a complete knockout of PCBP1 in multiple cell lines, but given that PCBP1 knockout is embryonically lethal in mice, it appears that full loss of PCBP1 is not tolerated in vitro either. Thus, the residual PCBP1 band observed likely reflects incomplete knockout and/or selective pressure to retain minimal protein expression for cell viability.

Importantly, even this partial reduction of PCBP1 significantly impairs cGAS–STING pathway activation, consistent with the functional relevance of PCBP1 levels. Moreover, this model may be more clinically relevant, as breast cancer patient tumors exhibit partial reductions in PCBP1 mRNA expression (Figure 1A), rather than complete loss. We now mention a partial knock-out in the revised text to avoid confusion.

Line 148: "Notably, PCBP1 knockout in Py8119 cells is only partial (Fig. 3F), as a complete loss may impair cell survival, consistent with the embryonic lethality observed in PCBP1-null mice[43]. Nevertheless, even partial loss was sufficient to significantly reduce ISG expression (Fig. 3E-F)."

6. Figure 4D and 4E (Vehicle Control and cGAMP Production):

• The identity of the vehicle control is not specified. Please clarify what substance was used.

We clarified in the manuscript that the vehicle condition used is lipofectamine 3000 without DNA. Line 853, legend of Figure 5G: "as well as its associated western blot on these same samples for the indicated markers after treatment with vehicle (lipofectamine 3000 only) or 1.5µg of HSV120 ssDNA or 1.5µg of HSV120 dsDNA +/− cGAS inhibitor (TDI-6570) for 20 hours."

• The vehicle appears to induce cGAMP production, which is impaired in KO cells. Why does this occur?

We note that measurable cGAMP is present not only in vehicle-treated cells but also in completely untreated cells. This reflects basal activation of the cGAS–STING pathway, which is expected due to cytosolic DNA fragments arising from micronuclei, mitochondrial leakage, or replication stress. Indeed, we detect low levels of basal cGAMP in several models, including Py8119, EMT6, and HMLE cells.

This basal activity is significantly diminished in PCBP1 KO cells, highlighting PCBP1's role in amplifying cGAS activation. The effect is most apparent under non-saturating conditions, when cytosolic DNA is limiting. Under these circumstances, PCBP1 facilitates more efficient sensing, resulting in a stronger cGAS–STING response. By contrast, when cGAS is saturated with excess DNA, PCBP1 is no longer required, which explains why transfecting large amounts of dsDNA initially masked the effect in our first version of the manuscript. We clarified the mechanisms in this new version. Thus, PCBP1 enhances the sensitivity of cGAS to low levels of cytosolic DNA, but does not alter the maximal response achievable under DNA saturation.

• A negative control is missing: The authors should show cGAMP levels in untreated cells (without vehicle or ssDNA).

We added cGAMP levels in untreated cells (Figure 5F, Figure S4A)

• While there is a clear difference in cGAMP production between WT and KO cells, this difference is also observed with the vehicle treatment, not only with ssDNA stimulation. Please clarify this observation.

In our revised experiments, after optimizing the transfection protocol and adding more biological replicates, we find that ssDNA does not significantly increase cGAMP production beyond the basal/vehicle condition, whereas dsDNA increases cGAMP response. This is consistent with prior reports showing that cGAS is preferentially activated by double-stranded DNA. The basal cGAMP signal detected under untreated or vehicle conditions likely reflects low-level sensing of endogenous cytosolic DNA fragments. Importantly, both this basal activity and the dsDNA-induced response are significantly reduced in PCBP1 KO cells, provided that cytosolic DNA levels remain under sub-saturating conditions. These findings support our conclusion that PCBP1 potentiates cGAS activation. Furthermore, by using the GDDG mutant, we demonstrate that PCBP1's single-stranded DNA–binding activity is essential for enhancing sensing, even of dsDNA molecules containing polyC sequences, as detailed in Figures 5, 6 and 7.

• The difference between ssDNA and vehicle-induced cGAMP production is minor, which raises concerns about whether ssDNA truly activates cGAS-STING in the presence of PCBP1.

Indeed, ssDNA does not activate much cGAS. We have clarified the mechanism (Figure 5, 6, 7, see comments above).

• To strengthen the argument, the authors should measure cytokine production (e.g., IFN-β, ISGs) via ELISA or qPCR to confirm that ssDNA in the presence of PCBP1 can activate the pathway.

We now have figure 7 which clarifies the mechanism: PCBP1 increases cGAS affinity for DNA containing accessible single-stranded polyC sequences. This further explains the downstream activation of STING mediated by PCBP1.

• To provide definitive evidence, the authors should perform experiments using either a cGAS knockdown (KD) or knockout (KO) (or sting). This would confirm whether ssDNA-mediated cGAS activation truly requires PCBP1.

We added a cGAS-inhibitor in Figure 5F which confirms that PCBP1 acts indeed through cGAS. Figure 7 also shows the direct effect of PCBP1 on cGAS.

• Please indicate whether cGAMP was measured in the cell supernatant or cell lysates.

7. Figure 5E (dsDNA Results): The manuscript does not discuss the results shown in Figure 5E (dsDNA condition). Please provide an explanation and interpretation of these findings.

The results are now all discussed (lines 194 to 214).

We clarified that cGAMP was measured in cell lysates, line 850, legend Fig. 5F: "cGAS activity assay measuring 2′3′-cGAMP production in cell lysates by ELISA (Mean ± SEM, two-tailed unpaired t.test, asterisks indicate statistical comparisons between each bar and the immediately preceding condition)."

8. Figure 5F (Biotin-RNA vs. DNA): The authors use biotinylated RNA, yet the central hypothesis focuses on ssDNA-mediated activation of cGAS. Why was RNA used instead of DNA? The authors should repeat this experiment using biotinylated DNA to align with their hypothesis.
We have repeated the pull-down with all the different oligos used in the study and clarified the mechanism (Fig. 6F, 7E, S5)

9. Figure 6E (Legend Formatting): The label "E" should be bold in the legend for consistency.
This has been changed.

10. Possible Explanation for ssDNA Activation of cGAS: It is possible that ssDNA forms secondary structures, allowing it to interact with cGAS. The authors should consider discussing this as a potential explanation in the Discussion section.
We have clarified the mechanism of action (see Figure 5, 6 and 7)

Reviewer #2 (Remarks to the Author):

In this manuscript, Fréreux and colleagues investigate the role of Poly(rC) Binding Protein 1 (PCBP1) in antitumor immunity in breast cancer. Using immunocompetent mammary tumorigenesis mouse models and murine in vitro breast cancer models, they show that PBCP1 depletion curbs baseline expression of interferon (IFN)-stimulated genes (ISGs) within malignant cells, enhances tumor growth and is associated with a broadly immunogenically colder, cytotoxic T cell-depleted tumor microenvironment. Through mechanistic in vitro dissection of PCBP1's mode of ISG activation, the authors propose that PCBP1's function as a poly(C)-rich region-binding protein facilitates single-stranded DNA (ssDNA)-mediated activation of cGAS and the downstream STING–interferon pathway, linking its canonical role to the authors' observed immune phenotypes. The characterization of in vivo PCBP1-/- immunophenotypes is very well-executed and the authors' conclusions are validated using multiple orthogonal approaches, including bulk and single-nuclei transcriptome sequencing, as well as flow cytometric and immunohistochemical analyses.

The dissection of the mechanistic link between PCBP1 and cGAS activation is weaker and more reliant on circumstantial evidence. Different cell lines, including murine breast cancer and human mammary epithelial cells, are profiled through immunoblotting and/or RT-qPCR to assess (STING pathway-associated) type I IFN activation markers in PCBP1 depletion contexts. However, which cell lines are profiled using which querying method and which markers are used to evaluate activation is inconsistent throughout experiments and the reason for said inconsistencies and why certain lines are chosen over others is not provided.
The evidence for the proposed PCBP1-mediated and selective binding of cGAS to ssDNA is reliant predominantly on REMSA / EMSA and cGAMP ELISA read-outs. This link, especially, would benefit from further strengthening through, for instance, additional profiling of PCBP1-dependent ssDNA-driven cGAS activation or pull-down assays with additional nucleic acid species beyond BAT RNA.

Some comments to consider.

Related to figures:

1. Fig. 2A. The y-axis cut-off on the volcano plot implies a p-value cut-off of 0.001, whereas the text mentions a cut-off of 0.0001.

This has been corrected. The cut off was 0.001. Line 104 "Among the 58 most significantly downregulated genes ($p < 0.001$, fold change < –2) in PCBP1-deficient tumors, 34 were annotated as interferon-stimulated genes (ISGs) according to the Interferome database[42] (Fig. 2A)."

2. Fig. 2B. The text mentions 17/20 shown genes are ISGs, though these are not clearly annotated.

These genes are now clearly mentioned in the main text. Line 107 "Of the top 20 most significantly downregulated protein-coding genes, 18 were ISGs, with the exception of *Apol9b* and *Zfp781* (Fig. 2B)."

3. Fig. 2D. Plots are grainy and hard to read.

All the plots should be good quality and easier to read now.

4. Fig. 2E, 3E and similar. Add Y-axis label rather than "(F.C.)" above figures.

This has been changed

5. Fig. 2F. There is no mention of hets in the main text. Densitometric analysis of blots might show a progressive decrease in IFN pathway components across the three genotypes.

We have added a discussion about hets in the main text. Line 96: "Heterozygous PyMT PCBP1+/− tumors displayed an intermediate phenotype, with a partial increase in tumor number (Fig. 1E) and burden (Fig. 1F) compared to PyMT PCBP1+/+ controls, suggesting a dose-dependent effect of PCBP1 on tumor suppression."

6. Fig. 2G. This could benefit from a zoomed in area of interest to highlight pronounced selective downregulation in mammary cancer cells, which the main text highlights.

We have included a zoomed in area for Figure 2G.

7. Fig. 3B. Whilst this representation shows this quite clearly for the two selected ISGs (Isg15, Oas2), it is not an ideal representation of the data, as it's not clear how many cells of the luminal epithelial mammary gland cluster were contributed by each genotype and, hence, what the relative proportion of selected ISG-expressing cells is for each genotype. A heatmap similar to what the authors use in Figure S1A (showing both average expression and % expression) would be more effective and would allow inclusion of more ISGs beyond (Oas2 and Isg15), as well as better assessment of cell cluster selectivity of ISGs.

We agree that a heatmap reporting both mean expression and the proportion of expressing cells is informative. We have included Supplementary Fig. S1B showing ISG expression in each cell type for WT vs KO tumors. We still kept the UMAPs of OAS2 and ISG15 in the main figure (Fig. 3B) because they better illustrate that ISGs are regulated predominantly in mammary epithelial cells, together with the complementary plot in Fig. 3C. This is because the heatmap does not compare cell proportions between cell types. Mammary epithelial cells are highly predominant (~80% of the tumor; Supplementary Table S2) and, therefore, when analyzing ISG expression at the whole-tumor scale, changes appear significant mainly in this epithelial compartment (Fig. 3B–C). However, when the analysis is broken down by cell type (Fig. S1B), ISGs are also regulated in additional, less abundant lineages. Moreover, only the epithelial cells show a consistent downregulation across all listed ISGs, whereas other cell types downregulate only some of them in PCBP1-KO tumors. This pattern is expected given that type I IFNs are secreted within the tumor microenvironment, so their loss can reduce ISG expression across multiple cell types, with the strongest effect observed in the numerically dominant mammary epithelial cells.

8. Fig. 3C. It is not clear what has led to certain genes being assigned one cell type over another. What analysis was performed to enable the highlighting of ISGs as mammary epithelial cell specific? This needs a more detailed explanation in either text or figure legend.

To assign the mammary epithelial cell cluster in our snRNA-seq analysis, we used expression of the PyMT transgene together with EpCAM, a well-established epithelial marker. PyMT encodes the polyoma middle T antigen, an oncogene derived from the mouse polyoma virus. Because it is not encoded in the mouse genome, PyMT is absent from normal mouse tissues; in our model, it is introduced under the MMTV promoter to drive transformation of mammary epithelial cells. This specificity, combined with EpCAM expression (a common epithelial cell marker), allowed us to confidently annotate the PyMT mammary epithelial population and assess ISG expression specifically within this cluster. The expression profiles of PyMT, EpCAM, and other genes used for cell type annotation are shown in Figure S1A.

9. Fig. 3E. Need to demonstrate PCBP1 O/E (for PCBP1-/- V5-PCBP1) by immunoblot alongside PCBP1-/- and PCBP1+/+ samples. Can the increase in ISGs/IFNB1 in V5 be ascribed to O/E of PCBP1 beyond endogenous levels?

Our anti-PCBP1 antibody does not detect the V5-tagged construct, likely due to epitope masking by the tag. We therefore confirmed the expression of V5-PCBP1 by PCR using Forward PCBP1 and Reverse-V5 specific primers on reverse transcribed mRNA from these cells (Figure 5E). The increase in ISGs/IFNB1 in V5-PCBP1 cells is indeed due to overexpression of PCBP1 beyond endogenous levels. We confirmed the overexpression of PCBP1 by qPCR compared to WT Py8119 cells (Fig. S1C). Moreover, the increase in cGAMP production is not observed with the PCBP1 GDDG mutant, indicating specificity of the effect to PCBP1's DNA binding activity (Figure 5F).
We clarified this in the main text, line 144: "We next validated these findings *in vitro*. In Py8119 mesenchymal-like mouse triple negative breast cancer cells, CRISPR-Cas9-mediated knockout of PCBP1 led to marked reduction in *ISG15, IRF7*, and *IFNB1* mRNA levels compared to control cells (Fig. 3E). Overexpression of PCBP1 in knockout cells (Fig. S1C) restored and even enhanced their expression relative to wild-type levels, confirming PCBP1 as a strong inducer of ISG and *IFNB1* transcription."

10. Fig. 4B, C; S2A, B, C. All phospho-protein blots need matched whole-protein blots (e.g. p-STAT1, but no STAT1 in 4B; p-STING but no STING in 4B). Inconsistencies in the order in which blotted proteins are displayed between 4B and 4C affects legibility. Some used markers, such as IRF3, TBK1 and cGAS by themselves are not good indicators of cGAS-STING pathway activation.

Whole protein blots have been added. We have 2'3'cGAMP production assays to confirm that the effect is cGAS mediated and included a new figure (Figure 7) that shows exactly the mechanism of action of PCBP1 on cGAS directly: PCBP1 increases cGAS affinity for nucleic acids containing accessible polyC sequences but does not affect the catalytic activity of cGAS itself.

11. Fig. 1C, 2G, 6D. Scale bars needed on all ICC images.

Scale bars were added to all microscopy images.

Additional comments:

The n numbers on experiments are often not clear – especially where representative images have been used (e.g. immunoblots) or barplots have been used for data presentation (e.g. RT-qPCRs).

We have clarified the n numbers in the legends and changed the bar plots to represent individual values instead.

The authors refer twice in the main text to the use of human breast cancer cells in the study. However, HMLE is as far as I'm aware the only human line used and is a non-cancerous epithelial line.

We corrected this. Indeed, the mechanism seem to be happening also in normal mammary epithelial cells. Because PCBP1 alone has a direct effect on cGAS affinity for nucleic acids (see enzyme kinetics, Figure 7). It is likely that PCBP1 can have an effect on different cell types that express both PCBP1 and cGAS.

Reviewers' comments:

Reviewer #1 (Remarks to the Author):

Comment:
• Page 5: Similar results were obtained in the EMT6 breast cancer cell line using shRNA-mediated PCBP1 silencing, with significant downregulation of IFIT1 and IFNB1 (Fig. 3E). However, in the figure there are not data shown for IFNB in EMT6 cells.
This was a mistake, we modified line 147 and it now mentions: "Similar results were obtained in the EMT6 breast cancer cell line using shRNA-mediated PCBP1 silencing, with significant downregulation of Isg15 and Ifit1 (Fig. 3E)."

Reviewer #2 (Remarks to the Author):

In this revised version of their manuscript, Fréreux and colleagues include significant experimental additions addressing, in particular, reviewer concerns centered around the initially proposed mechanism of PCBP1-mediated recruitment of cGAS to ssDNA. The main approach(es) taken to address reviewer concerns is using various oligonucleotide species (HSV120 ss/dsDNA, BAT RNA, G3/C4-YSD) in in vitro pull-down assays and EMSAs.
These revision experiments significantly clarify the mechanistic part of the manuscript and have resulted in an updating of their previous mechanistic model to a more nuanced one: wherein PCBP1 helps recruitment of cGAS to (ds)DNA comprising poly-cytidine ssDNA tracts (e.g. poly-C overhangs).

In addition to added experimental data, other changes made to the text (points of clarification), figures (replotting to reflect underlying sample sizes; addition of a hypothetical model figure / Fig.8) and figure legends (clarification of sample sizes and stats) have resulted in a substantial improvement of the manuscript.

Remaining comments:

1) Line 110-111: "The downregulation of ISG expression, including ISG15, IRF7, IFIT1, Rsad2, and Bst2, as well as IFNB1, was validated by qPCR in PyMT PCBP1–/– tumors (Fig. 2E)."
The murine naming convention (Isg15, Irf7, …) should apply consistently to all mouse genes.
This has been corrected throughout the manuscript; all mouse gene names are now written according to the appropriate murine nomenclature (e.g., Isg15, Irf7) in the main text and figure legends. Gene symbols were kept in uppercase within the figures for readability but can be modified upon request.

2) Enrichment plots in Fig. 2D and 4B should show statistical outputs (NES, adjusted P/FDRq).
The exact NES and FDR q values have been added in the legends of Fig 2D and 4B.

- **<u>Line 802:</u>** "Enrichment plots of 'Response to Type I Interferon' (NES = -2.76, FDR q-value <0.0001) and 'Type I Interferon Production' (NES = -2.60, FDR q-value <0.0001) gene sets based on GSEA analysis (M5 ontology)"
- **<u>Line 835:</u>** "Enrichment plots of 'Interferon Gamma Response' (NES = -3.06, FDR q-value <0.0001) and 'TNFalpha Signaling via NFkappaB' (NES = -1.82, FDR q-value = 0.0011) gene sets from GSEA analysis with MSigDB Hallmark (H) based on bulk RNA-seq data from PyMT Pcbp1–/– vs PyMT Pcbp1+/+ mammary tumors."

3) Whilst the used sample sizes for each experiment are now much clearer thanks to the authors' replotting of a lot of the data, it is still not clearly stated how many independent experiments the images of immunoblots and EMSAs are representative of.
All experiments were reproduced at least three times independently. Some immunoblots were performed more than three times throughout the study. To clarify this and avoid redundancy across all figure legends, we have added a sentence in the 'Statistical Analysis' section specifying that all EMSAs, immunoblots/pulldown assays were independently repeated at least three times.

- **<u>Line 578:</u>** "Representative experiments were independently repeated at least three times, including all electrophoretic mobility shift assays (EMSAs), immunoblots, and pulldown assays presented in this study."

4) A repetition of a previous point: All phospho-protein blots need matched whole-protein blots and vice-versa for phospho-indicators of cGAS-STING pathway activation (IRF3, TBK1, STING, STAT1).

For instance:
Fig.S4D – No whole-protein STING to go with p-STING.
Fig. S4F – No p-TBK1 to go with TBK1 whole-protein.
Fig. 5B-C – Whole-proteins without activation marks (IRF3) or activation marks (phospho) without whole protein (e.g. p-STAT1).

In some instances (e.g. Fig.5B), slimming down blots by removing such 'unmatched' and inconsistently used markers would improve clarity across figures.

We slimmed down the blots in Fig. 5B–C to make them more consistent. The p-TBK1 and total TBK1 blots that were previously shown in Fig. 5B, as well as the cGAS blot that was previously in Fig. 5C, were moved to Supplementary Figs. S4A and S4B, respectively. These panels were kept in the supplementary data, as p-TBK1 remains an informative marker of impaired pathway activation in Py8119 cells, and showing unchanged cGAS levels confirms that PCBP1 regulates cGAS activation rather than its expression.

Additionally, IRF3 in this context serves as an interferon-stimulated gene (ISG) marker. Although IRF3 is phosphorylated to induce type I IFN expression, type I IFNs in turn upregulate the transcription of ISGs, including IRF3 and IRF7 themselves. Therefore, total IRF3 levels reflect IFN pathway activation in these experiments, similar to ISG15.

Measuring p-IRF3 would be redundant here, as its levels directly depend on total IRF3 abundance.

We also corrected blots in Figs. S4F and S4H to display the corresponding total and phosphorylated markers.

All legends and main figure references have been modified accordingly.

5) Line 229-231: "cGAS was titrated in the presence of a constant amount of PCBP1, the PCBP1/oligo complex disappeared and a super shifted complex appeared, consistent with simultaneous binding of both proteins to the same ssDNA molecule (Fig. 6A left panel).
Should this not say "Fig. 6A right panel"?
Absolutely, we changed this for "Fig. 6A right panel", line 231.

6) Line 236 and other: Should refer to "Fig. S5" not "S4" – ditto for other mentions of 'Fig.S4'.
Indeed. We corrected that (lines 238, 241 and 242).

7) Line 235: Compared to the previous version of the manuscript, this version does not introduce or define 'BAT RNA'. Doing so would help clarify those data.
We added back the introduction of the BAT RNA line 235: "We further tested a single-stranded RNA control, the BAT element (BAT RNA), a 3′-UTR motif containing consecutive cytidine (rCrCrC) repeats previously shown to bind PCBP1 and regulate mRNA translation[33]. The same results were obtained for PCBP1 and cGAS binding using this BAT RNA sequence and its G-mutant counterpart (Fig. S5A–C)."

8) Reference 65 and 66 in the text map to references 66 and 67 in the reference list, respectively. It is worth checking whether this mismatch isn't more pervasive across in-text references.
We fixed any mismatching references.

9) Line 273-277: It is not entirely clear on first pass that the ultimate read-out used for the kinetics assay is cGAMP ELISA-based. It is worth underscoring this in the text.
We clarified this in the main text:

- **Line 275:** "To functionally test this model, we performed cGAS enzyme kinetics measured by a 2′3′-cGAMP ELISA-based assay using C4-YSD, HSV120 dsDNA"

10) Fig.7E and Fig. S6A-C – It is unclear what cell lines / lysates pull-downs were performed on. Please clarify in the legends.
We clarified this in the legends (lines 948, 950, 952, 954).

11) The C4-YSD oligo created by the authors seems to have very low inherent affinity for cGAS (Fig.7B and E). This chimes with results from the original study which derived G3-YSD (doi: 10.1038/ni.3267), showing very little interferon induction upon transfection with a C3-YSD (polyC overhang) variant of the oligo.

Can the authors clarify the use of this oligo species with low cGAS affinity and how it relates to their model?

The C4-YSD oligo was intentionally designed as a mechanistic tool to characterize how PCBP1 modulates cGAS activity *in vitro*. Because cGAS preferentially senses double-stranded DNA or DNA with secondary structures (such as Y-form DNA), whereas PCBP1 binds only single-stranded poly-cytosine tracts, we required a ligand that could both engage cGAS (even if it's with lower affinity) and present accessible single-stranded polyC motifs for PCBP1 binding. In a cellular context, PCBP1 can associate with polyC regions within double-stranded DNA through protein partners that most likely facilitate local unwinding; however, this is not possible in a recombinant *in vitro* setting. We therefore generated the C4-YSD variant, which contains four consecutive cytosines, which is the optimal binding motif length for the KH domains of PCBP1.

Consistent with previous observations that the C3-YSD variant shows weak cGAS activation (doi: 10.1038/ni.3267), the C4-YSD displayed low intrinsic affinity for cGAS but was sufficient to support measurable 2′3′-cGAMP production *in vitro*. Importantly, this setup enabled us to detect a reproducible and significant enhancement of cGAS activity in the presence of PCBP1 under sub-saturating conditions, even with a low-affinity ligand, but this should be applicable with a broader range of polyC DNA such has the HSV120 dsDNA in a real cellular context (when under-sub saturating concentrations).

Were any experiments done to test its functionality as a cGAS agonist?

C4-YSD was used primarily to demonstrate the mechanistic principle rather than its functional potency as a cGAS agonist in cells. We have not performed transfection experiments with C4-YSD, as our goal was to dissect the molecular mechanism of PCBP1-mediated enhancement of cGAS activity.

We believe that further work will be needed to identify the endogenous sources and types of polyC-containing DNA on which PCBP1 may enhance cGAS sensing in a physiological cellular context. We clarified this point in the discussion:

- **Line 352:** "In our study, we used transfected DNA oligos and an engineered C4-YSD construct as defined experimental tools to dissect the molecular mechanism of PCBP1-mediated cGAS activation. However, future work will be needed to identify the endogenous sources and types of polyC-containing DNA on which PCBP1 may enhance cGAS sensing in a physiological cellular context."

Do they suggest PCBP1 mediates binding of cGAS to nucleic acid substrates cGAS would otherwise have low or no affinity to for (increased repertoire), or that it broadly improves affinity to all its substrates?

Our data support a model in which PCBP1 enhances cGAS affinity for all DNA substrates containing accessible polyC motifs, provided these ligands adopt conformations capable of positioning cGAS in its active state (e.g., double-stranded or Y-form DNA rather than purely single-stranded DNA). We show that PCBP1 acts under sub-saturating conditions, when DNA availability is limiting relative to cGAS, thereby increasing the apparent affinity of cGAS for these substrates and promoting higher 2′3′-cGAMP production.

Because high-affinity ligands saturate cGAS at lower concentrations, PCBP1 operates within a narrower effective range, facilitating early activation when DNA is scarce.

In contrast, low-affinity ligands require higher concentrations to saturate cGAS, giving PCBP1 a broader concentration window in which to enhance cGAS sensing and help reach the threshold of 2′3′-cGAMP production necessary for downstream STING activation.

Under physiological conditions, where cGAS is rarely saturated due to the limited and transient nature of cytosolic DNA, PCBP1 is therefore expected to modulate a wide range of cGAS substrates, provided they contain polyC tracts that serve as binding sites for PCBP1.
